# Use of a Smartphone-Based App in an Online Behavioral Neuroscience Lab Course

**DOI:** 10.59390/001c.163102

**Published:** 2026-07-01

**Authors:** Beatrice de Oca

**Affiliations:** 1 Psychology California State University, Channel Islands

**Keywords:** inquiry-based learning, online education, biopsychology, lab activity, behavioral neuroscience

## Abstract

Course-based research can promote the development of scientific skills and improve understanding of the scientific method. However, these types of inquiry-based research experiences can be challenging to implement with online courses. I therefore designed a course-based undergraduate research experience suitable for an asynchronous online lab course in Biopsychology. Students in two online courses in Behavioral Neuroscience proposed and completed a study using a smartphone-based app that measures heart rate variability (HRV). Students developed background context on HRV as an index of parasympathetic nervous system activity. Then, students proposed an experiment to examine the effectiveness of a stress reduction intervention. Students measured HRV and subjective mood and stress before and after the intervention. Students summarized their project outcomes in a presentation. Student feedback regarding their engagement throughout the project was positive. In terms of student learning, students increased their agreement with statements reflecting mastery of one course learning outcome from the start to the end of the course. They also reported that the research project, including use of the HRV measurement app, was on average very important in learning about each of the learning outcomes. This project allows students in asynchronous online biopsychology courses to benefit from inquiry-based research.

Research training in lab courses and extra-curricular research experiences help prepare students for graduate study in Neuroscience, Psychology, Biology and related fields (Crowe & Brakke, 2008; Seymour et al., 2004). Many barriers, including resources and funding, make extracurricular research experience for all students unlikely (see Spell et al., 2014, for a discussion). While many majors, including Psychology, include laboratory courses, these courses may involve standard or “cookbook” experiments rather than original research incorporating inquiry-based learning (Rissing & Cogan, 2009; Spell et al., 2014). However, the standard “cookbook” lab does not promote significant improvements in student confidence in their scientific skills and self-assessed knowledge of research techniques compared to an inquiry-based lab (Rissing & Cogan, 2009). Course-based undergraduate research experiences (CUREs) successfully encourage engagement in science, enhance scientific skills and improve understanding of the process of science (Lloyd et al., 2019). They can also improve scientific efficacy and students’ collaboration and communication skills, as in a CURE project in psychoneuroimmunology that allowed students to develop and test hypotheses concerning psychosocial variables and peripheral inflammation (Mesmer & Gaudier-Diaz, 2022). Even if a course does not incorporate a full CURE experience with genuine data collection, inquiry-based lesson plans may approximate the benefits of CUREs.

The inquiry-based project described here addresses four out of the five core competencies for neuroscience undergraduates outlined by the Society for Neuroscience, including conceptual knowledge, analytical and scientific thinking, rigorous and responsible conduct of research, and communication skills (Society for Neuroscience, 2026).

The American Psychological Association (APA, 2023) lists Scientific Inquiry as one of the five goals of the undergraduate Psychology major. The inquiry-based project presented here attempts to bolster skills in this area. While Psychology majors may improve in their understanding of scientific literacy as they advance through the major, there may not be much improvement in students’ view of Psychology as a science (Holmes & Beins, 2009). In other words, students do not seem to gain an appreciation for understanding how psychologists know what they know through their undergraduate training (Holmes & Beins, 2009). Lab work can help close this gap.

Unfortunately, psychology has relatively few lab courses compared to biology, chemistry, and other STEM programs, and most lab courses in the major focus on research methods and statistics. Lab courses that relate to particular content in psychology occur less often. Physiological psychology or neuroscience labs are found in only 28% of programs and cognitive psychology labs are found in only 24% of programs (Norcross et al., 2016).

An online curriculum poses challenges for laboratory courses. Online students may not be able to access the specialized equipment used for an in-person neuroscience lab (e.g. Wilson, 2024). University instructors who teach online STEM courses have responded to these challenges with CURE projects suitable for implementation in online courses. The COVID-19 pandemic prompted the movement of college courses to an online format during the Spring of 2020, leading many instructors to shift their CURE projects to an online setting. Broussard et al. (2021) reviewed the effectiveness of a variety of CURE projects that shifted from in-person delivery to a mix of synchronous and asynchronous online instruction during this period. Even though courses were not intended for online implementation, the CURE projects have been shown to successfully improve research skills and facilitate student development using a variety of measures. In terms of neuroscience education, instructors have successfully integrated digital brain images in online CURE projects (Delogu et al., 2025; Wickham et al., 2021). In one relatively short fully online project, students collaborated with each other to compare cortical neuron spine density and structural morphology between a mouse line with learning impairments and controls (Wickham et al., 2021). Participation in the lab resulted in improvements in content outcomes and STEM identity scores and led to decreases in negative attitudes about science. Moreover, direct comparison to the CURE database revealed that the online project produced benefits comparable to traditional CUREs. The authors noted that in addition to contributing valid scientific information, this project facilitated dramatically greater inclusion of students who would otherwise experience barriers to research participation (Wickham et al., 2021). In another example, students in an online introductory Behavioral Neuroscience course engaged in hypothesis testing using free open neuroscience resources (Delogu et al., 2025). Throughout the project, students took measurements of brain areas to answer questions related to cocaine use disorder, a variety of brain pathologies and tumor removal. Students analyzed their data and communicated their findings through a poster presentation.

Given the benefits of engaging students in these learning activities, I developed an inquiry-based research project for students enrolled in an asynchronous online laboratory course in Behavioral Neuroscience. The project involved detailed assignments with substantial scaffolding and the use of a smartphone app to measure heart rate variability (HRV). The app allows students to collect course-relevant physiological data without needing equipment or hands-on training. HRV is a measure of the variability of the heart’s inter-beat intervals and reflects the actions of the sympathetic and parasympathetic branches of the autonomic nervous system (Saul, 1990). Additionally, HRV reflects activity by brain regions involved in autonomic regulation, including the prefrontal cortex and anterior cingulate cortex (Thayer & Lane, 2000), making it relevant to students in a course in Behavioral Neuroscience.

## Theoretical background

The Neurovisceral Integration Model (Thayer & Lane, 2000) connects HRV with brain areas that make up the Central Autonomic Network (CAN) including the prefrontal cortex and anterior cingulate cortex. The model proposes that the functioning of CAN areas like the prefrontal cortex can influence HRV, and HRV can be used as an index of prefrontal cortex activity. Greater prefrontal cortex activity would allow for stronger vagal influence on the heart, resulting in greater variability in the heart rate as the vagus nerve slows heart rate during exhalation. The theoretical background of this model aligns well with the content of the course in Behavioral Neuroscience and supports research on topics of interest to many students, especially Psychology majors.

After reading and summarizing research articles providing context, students selected a stress-reduction intervention. Students proposed a research study using this intervention and collected survey and HRV data from themselves to assess the effectiveness of the intervention. While the project’s subjective measures were vulnerable to demand characteristics and biased responses, the HRV measurement provided an objective measure of whether or not the intervention reduced stress.

This article will 1) Describe the use of a phone-based app to measure HRV as part of an inquiry-based project for an online course, 2) Describe assignments that help to assess course learning objectives related to scientific inquiry and critical thinking, and 3) Summarize student engagement, meeting of course learning outcomes, and student feedback regarding their experience with the project.

## MATERIALS AND METHODS

### Participants

All participants were enrolled in an online lecture and lab course in Behavioral Neuroscience offered by California State University Channel Islands. Participants provided informed consent upon volunteering to complete the surveys used to assess the research project assignment. The first assessment of this project occurred during the Fall of 2023 in an asynchronous online upper division Behavioral Neuroscience course with a lecture and lab. Of the 37 students in the course, 33 identified as female. Twenty-four students voluntarily participated in the survey and a total of 22 participants answered all survey questions. The second administration occurred during a five-week summer session in 2025. Of the 23 students enrolled in the course, eight completed both the pre and post project surveys. Five participants identified as female, one identified as both female and non-binary and two identified as male. All participants were juniors or seniors. The California State University Channel Islands Institutional Review Board approved both survey administrations (IO5673 and I05753).

### Project Assessments

The research project was incorporated into the lab during two semesters. Students completed a single project assessment survey in the 2023 administration and a pre and post project survey in 2025.

During the first survey administration in 2023, students indicated their agreement with a statement that they learned a lot from completing the research project in the course, including the proposal, data collection, and the presentation, using a 5-point Likert-type scale (1 = Strongly Disagree; 5 = Strongly Agree). Students also reported agreement with a statement indicating they found data collection using the HRV app to be very engaging (1 = Strongly Disagree; 5 = Strongly Agree). They were also asked what, if anything, they liked about the research project and what they would change about the written assignments that were part of the research project. The survey also prompted students to provide their college classification, gender, ethnicity and age.

The format of the 2025 surveys was changed by addition of the Student Course Engagement Questionnaire modified for online courses (SCEQ-M; Nasir et al., 2020). This is a validated measure of student engagement and replaced the unvalidated questions used in the 2023 survey. The SCEQ-M scale allows for particular aspects of engagement to be assessed, including goal-oriented engagement, self-disciplined engagement and interactive engagement. The SCEQ-M consists of 19 questions that assess various aspects of course engagement and demonstrated an overall reliability of .861 when used in online courses (Nasir et al., 2020).

The 2025 survey also added an assessment of course learning outcomes. Students rated their agreement with statements that reflected three course learning outcomes. The statements were: “I have a thorough understanding of how psychology relates to the natural sciences, including biology, physiology, medicine and neuroscience,” “I know the main theories and research that explain the biological and physiological foundations of behavior,” and “I can explain how brain structures, neurochemistry and brain functioning underlie behavior.” The survey used a 4-point scale (1 = Strongly disagree; 4 = Strongly agree),and was similar to the method developed by Combs et al. (2008). Both pre and post project surveys administered in 2025 included these items. On the post-project survey, students also rated how important the research project, including use of the HRV app, was in helping them meet each outcome using a 5-point scale (1 = not at all important, 2 = slightly important, 3 = moderately important, 4 = very important, 5 = extremely important).

### HRV Data Collection Procedures

**Figure 1.**
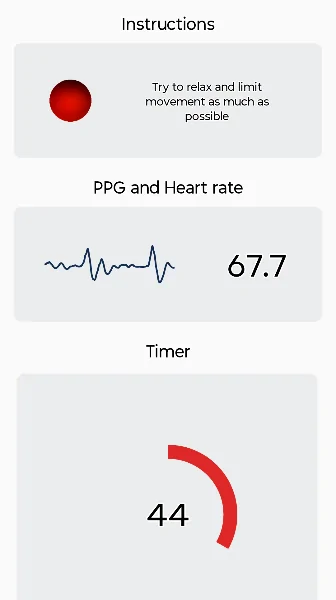
A screenshot of the Camera Heart Rate Variability app.

The Camera HRV app uses a phone’s camera to detect heart activity by measuring illumination from the flash through the person’s finger using photoplethysmography (PPG). The Camera HRV app is available for both iPhone and Android phones. For iPhone, see https://apps.apple.com/us/app/camera-heart-rate-variability/id788460316. For Android, see https://play.google.com/store/apps/details?id=com.asma.camerahrv&hl=en. See Figure 1 for a screenshot of the app as viewed on an Android phone. Students who don’t have access to a smartphone can use any wearable HRV device including an Apple watch or Polar chest strap heart rate monitor. A free phone-based app that measures HRV using the phone’s accelerometer rather than the camera is also available. For iPhone, see https://apps.apple.com/fi/app/kubios-hrv-daily-readiness/id1463040412. For Android, see https://play.google.com/store/apps/details?id=com.kubioshrvapp.

One important question is whether the phone app can reliably measure HRV without a chest strap or an electrocardiogram (ECG). HRV measurement relies on detecting the intervals between R waves (R-R intervals; Johnston et al., 2020). A comparison of R-R data obtained from 29 participants using ECG and PPG using the same app technology as Camera HRV revealed a perfect correlation (r = 1.0) between the signal obtained via ECG and that obtained via the Camera HRV smartphone app (Plews et al., 2017). The Kubios app that uses a phone’s accelerometer has also been found to be highly reliable and valid (Gabani & Murugan, 2024)

### Lesson Content

With the availability of a practical method for obtaining HRV with online students, the next step was developing assignments that would allow students to meaningfully engage in inquiry-based learning.

**Figure 2.**
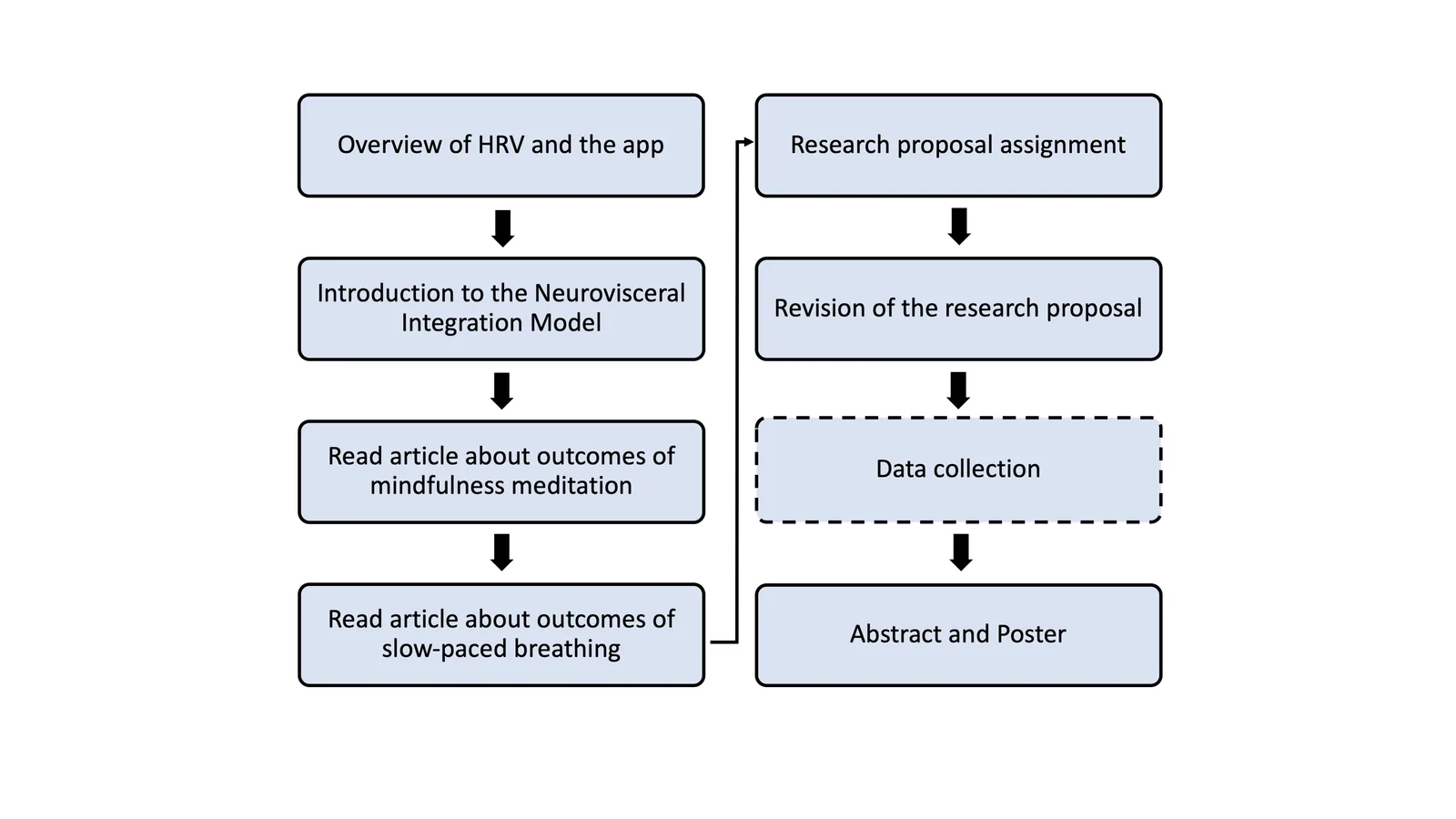
Overview of the research project.

First, I provided a short lecture on the Neurovisceral Integration Model (Thayer & Lane, 2000) describing how particular areas of the brain regulate and receive information from various systems in the body, including the cardiovascular system. To develop relevant background literature, students read two research reports of the effectiveness of a stress reduction method. Using a series of questions to help guide them, they discovered how researchers examined the efficacy of a brief slow breathing activity (Laborde et al., 2022) and an hour-long mindfulness activity (Shearer et al., 2016) on HRV. Both a single slow-paced breathing intervention and a four-week mindfulness meditation intervention significantly elevated HRV and subjective mood states. The slow-paced breathing intervention also significantly improved executive function. With this background information, students prepared a research proposal in which they selected a stress reduction intervention to implement on themselves. See Figure 2 for an overview of the assignments. A full description of the assignments given throughout the semester can be found in Appendix A.

## RESULTS

### Subjective Student Feedback About the Project

As described earlier, subjective student feedback about the project was solicited using the 2023 survey. When asked to indicate agreement with a statement that they “learned a lot from completing the research project in the course”, the mean rating was 4.59 (n = 22, SD = .959), on a 5-point scale ranging from 1 = strongly disagree to 5 = strongly agree. When asked to indicate their agreement with a statement that they “found data collection using the HRV app to be very engaging”, the mean rating was 4.27 (SD = 1.16). When asked what, if anything, they liked about the research project, responses ranged from enjoying the process of learning about HRV, appreciating that the project allowed them to incorporate a stress reduction method into their routine, and engaging in a demonstration of how the project might be done at a larger scale. None of the participants reported not enjoying anything about the project. The responses were categorized as expressing appreciation for learning about slow breathing and mindfulness (13 of 22 responses) and/or as expressing an appreciation for how the project allowed them to learn about the research process (seven of 22 responses). Sample student comments include the following: “I liked how I was able to do it on my own time and it will help me figure out what I can do to help my stress for the next semester,” “What I liked about this project is that the study is relevant and encourages current students to engage in activities that will benefit them in their school careers;” and “I liked getting to see the potential set up of what a research project could look like at larger scale.”

### Student Course Engagement Questionnaire

As noted earlier, student engagement and attainment of individual course learning outcomes was measured with a separate survey during 2025. Students (n=8) reported high levels of engagement, with Goal-Oriented Engagement and Applied Engagement subscales receiving the highest ratings. The Interactive Engagement subscale received the lowest scores. See Table 1 for Correlations, Descriptive Statistics, and Reliability of the SCEQ-M. Applied Engagement refers to the emotional engagement of the student with the subject matter and the relevance of course content to student’s lives. High scores likely reflected the project’s personal relevance since students selected their own stress reduction measure and observed the benefits to their own physiological and subjective well-being. Goal-Oriented Engagement refers to skill development and learning outcomes and goals. As can be seen in Table 1, Goal-Oriented Engagement showed the strongest associations with other dimensions and the total score, Much research has documented that educational outcomes are strongly influenced by the level and type of student engagement (see Nasir et al., 2020 for a summary). Student responses to the SCEQ-M seem to reflect that the project successfully motivated student engagement.

**Table 1.** SCEQ-M correlations, descriptive statistics and reliability

Factor	M	SD	Reliability	Applied Engagement	Goal-Oriented Engagement	Self-Disciplined Engagement	Interactive Engagement
Applied Engagement	4.29	0.72	0.78	1.00			
Goal-Oriented Engagement	4.40	0.50	0.80	0.68	1.00		
Self-Disciplined Engagement	3.96	0.60	0.61	0.64	0.73	1.00	
Interactive Engagement	3.78	1.20	0.96	0.46	0.83	0.48	1.00
Total	4.08	0.64	0.92	0.73	0.96	0.80	0.89

### Course Learning Objectives

Students (n = 8) rated their agreement with statements reflecting that they met the course learning outcomes before and after completion of the project. A paired samples t-test with a Bonferroni correction (p = .0167) revealed significant improvement on the statement that students could explain how brain structures, neurochemistry and brain functioning underlie behavior [t(7) = 3.42, p = .011] from pre-test (M = 2.63, SD = .52) to post-test (M = 3.25, SD = .46). Scores on the learning objective concerning the main theories and research that explain behavior increased somewhat from the pre-test (M = 2.75, SD = .46) to the post-test (M = 3.38, SD = .52), but the increase was not statistically significant [t(7)=2.38, p = .049]. The outcome addressing how psychology relates to the natural sciences did not improve from before (M = 3.5, SD = .54) to after (M = 3.6, SD = .52) the project [t(7) = 1.0, p > .05].

Students also indicated the research project and the app were on average very important in helping them learn about each learning outcome. Students rated the project and app as “very important” in helping them learn about the outcome concerning how Psychology relates to the natural sciences (M = 4.13, SD = .64). They rated the project and app similarly on the outcome related to the theories and research that explain foundations of behavior (M = 4.0, SD = .54) and on the outcome related to how brain structures, neurochemistry and brain function underlie behavior (M = 4.0, SD = .54).

## DISCUSSION

Given that one goal of an undergraduate degree in the sciences is for students to develop skills in scientific inquiry, an inquiry-based research project can be a helpful component of a laboratory course. The project described here allowed students to develop hypotheses related to the effectiveness of a chosen stress reduction method. Overall, the use of a smartphone-based app for measurement of HRV facilitated successful data collection among students in an asynchronous online course. The project led to a high degree of engagement in the course and facilitated growth in one of the three course learning objectives.

The nature of the single-participant study they completed allowed students to choose a stress intervention related to mindfulness or a type of guided breathing like slow-paced breathing. Many students commented that they appreciated the opportunity to learn about and practice something that could help them cope with stress. This seemed to make the research project particularly relevant and engaging to students.

Accordingly, students reported high levels of engagement on the SCEQ-M with an overall average score of 4.0 on a 5 point scale. The Goal-Oriented and Applied Engagement sub-scales had the highest mean ratings of course engagement among students. Unfortunately, no comparison can be made with other courses taught by the same instructor. However, online graduate courses that used the SCEQ-M received ratings in the same range as those found in the current study (Nasir et al., 2020). An earlier in-person survey of 300 undergraduate students enrolled in agricultural education and leadership courses yielded a slightly lower overall engagement score of 3.39 (Marx et al., 2016). The SCEQ also accounted for 30% of the variance in student course grades in one study of freshmen in-person students (Handelsman et al., 2005), suggesting that it is linked with course performance. However, the SCEQ has some limitations. The factor structure of the original SCEQ scale with 23 items has not always been psychometrically supported (Masland et al., 2022). Yet, its ability to measure course-level engagement rather than program-level engagement makes it a useful instrument (Mandernach, 2015)

The project described here was also associated with successful attainment of course learning objectives. Students reported significant improvement on one of the three learning outcomes. Specifically, ratings for the learning outcome regarding how brain structures, neurochemistry and brain functioning underlie behavior improved significantly. To help determine how much the research project helped students meet these learning objectives, students also rated the importance of the research project and use of the HRV app in learning about each outcome. Students indicated the project and app were on average “very important” in helping them learn about each outcome.

A limitation of the survey of student learning outcomes is the inability to measure the project’s contributions towards student learning relative to other assignments. The student feedback reported here is encouraging, but to better isolate the benefits of the project, future research could include responses from students in a similar online course without this type of inquiry-driven research project.

The sample size is another substantial limitation of the assessment of the effectiveness of this activity. Only eight of 23 students from the 2025 course completed the SCEQ-M and the learning outcome survey. Students were asked to complete a survey at the start and end of the class in exchange for a small amount of course extra credit. The class used an accelerated format with a duration of only 5 weeks. The accelerated schedule and the small amount of extra credit may have deterred a higher participation rate among students.

After including this project in several asynchronous online sections, some of the chosen stress interventions appear to be more effective in improving students’ well-being. In general, student projects that used a slow-paced breathing intervention tended to positively influence stress, rumination and HRV, especially if practiced for at least 8-10 minutes each time. The effectiveness of engaging in slow breathing for this duration is consistent with other research. For example, Cheng et al. (2019) found that slow-paced breathing for a duration of 10 minutes successfully elevated HRV. Interventions of 3-5 minutes, walking meditation and YouTube video-guided meditation usually reduced subjective stress but did not elevate HRV. Even if the intervention did not successfully elevate HRV, indicating a lack of improvement in parasympathetic engagement, students often developed a positive impression of the intervention and its effects.

Overall, the research project described in this article provides a way of allowing online students to engage in inquiry-based research. A high degree of scaffolding is provided through the structured assignments, making the project manageable even with a relatively large number of students. After assigning the project to students over multiple semesters, I have not encountered much difficulty with the project or the technology. A minority of students asked questions about the assignments or requested clarification regarding the procedures. The research proposal assignment provides an opportunity to share detailed guidance with students before they start data collection. Students who need to correct aspects of their design or procedures do so through a separate proposal revision assignment.

Student feedback, both informal and through the official course evaluation instrument, has indicated that students generally find the procedures and assignments easy to understand. Given the asynchronous online nature of the course, it is important that all aspects of the project be clear and relatively easy to do without supervision or training. This project meets those requirements while supporting student engagement in inquiry-based research about a topic they find personally relevant.

### Address correspondence to:

Dr. Beatrice de Oca, Psychology, California State University, Channel Islands. Email: beatrice.deoca@csuci.edu

## Supplementary Material

Appendix A Description of assignments

Presentation Template

Video reviewing the Neurovisceral Integration Model

Article 1

Article 2

Revision of research proposal

Research Proposal Assignment

Article 2 questions

Article 1 questions

Survey about the app

Quiz about HRV

Question for Neurovisceral Integration Model

Neurovisceral Integration Model article
