# Supplementary material for: Use of a Smartphone-Based App in an Online Behavioral Neuroscience Lab Course
Source: J Undergrad Neurosci Educ. 2026 Jul 1;24(2):34–43. doi: 10.59390/001c.163102 (PMC13625900; doi:10.59390/001c.163102)
Supplement: Supplementary file 1 [file junejournal_2026_24_2_163102_348897.pdf]

Effects of a Brief Mindfulness Meditation Intervention on Student Stress and Heart Rate Variability

Annie Shearer, Melissa Hunt, Mifta Chowdhury, and Lorena Nicol
University of Pennsylvania

College can be a time of immense stress. Mindfulness meditation has been shown to be an effective stress management technique. A significant limitation of the mindfulness literature, however, is a reliance on inactive control groups. We compared a mindfulness intervention with both an ecologically valid, active control (interacting with a dog during a group study break) and a no-treatment control. Participants (n = 74) were randomly assigned to groups, with the treatment groups completing 4 weekly sessions (duration: 1 hr). By the end of the 4th session, those in the mindfulness group exhibited significantly lower state anxiety compared with those in the other groups, while the dog group was also significantly less anxious than the control group. In addition, both the dog and the mindfulness groups exhibited significantly less dysphoric affect than the control group. All of the participants came in for a posttreatment assessment during which they were given a cognitive stressor challenge. Electrocardiogram data were collected during the cognitive challenge allowing us to assess heart rate variability (HRV)—a measure of the body's ability to modulate the physiological stress response. Participants in the mindfulness group exhibited significantly higher HRV during the cognitive challenge than those in the other 2 groups, signifying a more-adaptive response to stress ($p < .05$). Individuals in the dog group, meanwhile, were no different from control participants. These preliminary findings suggest that brief mindfulness training can help college students manage their stress in response to the ubiquitous academic and cognitive challenges of college life.

Keywords: mindfulness, college, stress, heart rate variability, animal assisted therapy

This document is copyrighted by the American Psychological Association or one of its allied publishers.
This article is intended solely for the personal use of the individual user and is not to be disseminated broadly.

This article was published Online First October 12, 2015.

Annie Shearer, Melissa Hunt, Mifta Chowdhury, and Lorena Nicol, Psychology Department, University of Pennsylvania.

Annie Shearer is now at the Couple and Family Therapy Department, Drexel University. Mifta Chowdhury is now at MDRC, Health and Barriers to Employment, New York City, New York. Lorena Nicol is now at the VA Center of Excellence for Neurorestoration and Neurotechnology (CfNN), Providence VA Medical Center, Providence, Rhode Island.

Correspondence concerning this article should be addressed to Annie Shearer, Center for Family Intervention Science, 3020 Market St, Suite 510, Philadelphia, PA 19104. E-mail: shearera@sas.upenn.edu

International Journal of Stress Management
2016, Vol. 23, No. 2, 232–254
© 2015 American Psychological Association
1072-5245/16/$12.00 http://dx.doi.org/10.1037/a0039814

This document is copyrighted by the American Psychological Association or one of its allied publishers.
This article is intended solely for the personal use of the individual user and is not to be disseminated broadly.

College is stressful (Sax, 1997). Academic pressure coupled with the perception of inadequate time for study, leisure activities, and rest are significant stressors that contribute substantially to subjective stress (tension, anxiety, autonomic arousal) and strain, such as psychological symptoms of anxiety and depression among college students (Misra & McKean, 2000; Kerr, Johnson, Gans, & Krumrine, 2004). Poorly managed stress leads to poor adjustment during college and contributes to poor physical and psychological health. Attempts to reduce anxiety can result in increased use of potentially harmful coping strategies such as copious alcohol use and smoking (Pritchard, Wilson, & Yamnitz, 2007). Brougham and colleagues (2009) have found that both men and women college students tend to rely on emotion-focused coping strategies to deal with the discomfort of stress. They suggested that stress reduction interventions should focus on adaptive emotion-focused strategies.

Mindfulness-based stress reduction is just such an adaptive coping strategy (Bergen-Cico, Possemato, & Cheon, 2013). *Mindfulness* itself is defined as “paying attention in a particular way, on purpose, non-judgmentally, to the present moment” (Kabat-Zinn, 1994, p. 4). It has been shown to be correlated with a number of positive traits, including openness to experience, emotional intelligence, self-esteem, optimism, positive affect, life satisfaction, self-compassion, vitality, self-actualization, autonomy, competence, and relatedness fulfillment (e.g., Baer, Smith, Hopkins, Krietemeyer, & Toney, 2006; Brown & Ryan, 2003). Conversely, low levels of mindfulness have been linked to rumination, neuroticism, depression, anxiety, severity of psychological symptoms, difficulties in emotion regulation, avoidance, self-consciousness, social anxiety, negative affect, and absent-mindedness, to name a few (e.g., Baer et al., 2006; Brown & Ryan, 2003; Hollis-Walker & Colosimo, 2011).

Given the association of mindfulness with a wide-variety of positive outcomes, a central goal of mindfulness meditation is to help practitioners reach this adaptive state (Shapiro, Carlson, Astin, & Freedman, 2006). Indeed, clinicians and researchers alike have begun using mindfulness meditation techniques to enhance mindfulness (and thereby well-being) in both clinical and nonclinical populations. For instance, some have incorporated mindfulness meditation practices into programs for stress reduction or as components of therapy manuals. Perhaps the most well-known program is Mindfulness-Based Stress Reduction (MBSR), initially adapted from Eastern Mindfulness practices to treat chronic pain patients (Kabat-Zinn, Lipworth, & Burney, 1985). In early studies, participants exhibited significant reductions in pain, anxiety, and depression, with benefits maintained at 4-year follow-up (Kabat-Zinn, Lipworth, Burney, & Sellers, 1986). Later, similar reductions in anxiety and depression were reported for patients with anxiety disorders

(Kabat-Zinn, Massion, Kristeller, & Peterson, 1992), with gains maintained at 3-year follow-up (Miller, Fletcher, & Kabat-Zinn, 1995).

Overall, meta-analyses suggest that mindfulness training produces moderate to large effect sizes on measures of mental and physical health, anxiety, depression, and quality of life in both clinical and nonclinical samples (Grossman, Niemann, Schmidt, & Walach, 2004; Hofmann, Sawyer, Witt, & Oh, 2010; Vøllestad, Nielsen, & Nielsen, 2012). In addition, mindfulness training also reduces stress in nonclinical college populations. For example, Oman, Shapiro, Thoresen, Plante, and Flinders (2008) found that meditation training lowered stress and increased forgiveness among college students. Collard, Avny, and Boniwell (2008) found that mindfulness-based cognitive therapy improved both mindfulness and subjective well-being in similar samples. Shapiro, Schwartz, and Bonner (1998) found MBSR training significantly lowered depressive symptoms, anxiety, and psychological distress among medical and premedical students compared with waitlist controls. More recently, Shapiro, Brown, Thoresen, and Plante (2011) found that undergraduates who participated in an MBSR program had significantly higher trait mindfulness, subjective well-being, and empathy than controls, with gains maintained at 12-month follow-up. Even reduced mindfulness programs appear to benefit this population. For instance, Jain and colleagues (2007) found that four sessions (six hours) of mindfulness training significantly reduced distress, rumination, and distractive thoughts and increased positive mood in participants compared with waitlist controls. Their study was also one of the few to compare mindfulness training to an active control group (a multimodal relaxation intervention). They found no significant differences between the two interventions on short term distress or positive states of mind postintervention, suggesting that they controlled adequately for the immediate anxiolytic and group effects of MBSR. However, the mindfulness group showed a greater improvement in distractive and ruminative thoughts/behaviors at post.

This brings up an important limitation of the literature to date - despite a large number of studies and reviews demonstrating the positive effects of meditation, very few have compared mindfulness training with active control treatments (see MacCoon et al., 2012 and Jain et al., 2007 for exceptions). This gap is a common criticism in recent reviews and commentaries (e.g., Baer, 2003; Grossman et al., 2004; Ledesma & Kumano, 2009; Toneatto & Nguyen, 2007). For instance, Williams (2010) called for "trials, large and small, that compare mindfulness training with equally plausible active treatments" (p. 5). While preliminary research is clearly compelling, this gap cannot be ignored if mindfulness meditation is to be considered an empirically sound intervention (Chambless & Ollendick, 2001).

One highly plausible active control treatment is spending time with a therapeutic animal, especially a dog. Many colleges and universities through-

This document is copyrighted by the American Psychological Association or one of its allied publishers.
This article is intended solely for the personal use of the individual user and is not to be disseminated broadly.

This document is copyrighted by the American Psychological Association or one of its allied publishers.
This article is intended solely for the personal use of the individual user and is not to be disseminated broadly.

out the country are introducing programs in which students may interact with a dog (or dogs) at libraries (Aiken, 2014), recreation centers (Falcone, 2014), and during stressful exam periods (Schultheis, 2010). These programs are quite popular on college campuses and have been touted as a way to reduce stress for students. Therefore, we selected this active control treatment for its high degree of ecological validity. Time spent in a social setting with a friendly dog can also control for many aspects of a group MBSR intervention, including activity, attention, social interaction, and short term anxiety reduction.

More formally, Animal-Assisted Therapy or AAT is defined by Kruger, Trachtenberg and Serpell (2004, p. 4) as, "any therapeutic intervention that intentionally includes or incorporates animals as part of the therapeutic process or milieu," and has proven benefits across a large number of heterogeneous populations. For instance, it decreases agitation and loneliness, and increases the initiation of social interaction in the elderly (e.g., Banks & Banks, 2002), enhances self-efficacy and coping abilities among individuals with psychiatric diagnoses (Berget, Ekeberg, & Braastad, 2008), and reduces anxiety in psychiatric patients awaiting electroconvulsive shock therapy (Barker, Pandurangi, & Best, 2003). Fine and Beiler (2008) postulate that animals provide unconditional positive regard, while others have documented concrete physiological changes (e.g., lower cortisol and diastolic blood pressure) that result from interacting with companion animals (e.g., Allen, 2003; Somerville, Kruglikova, Robertson, Hanson, & Maclin, 2008). This suggests that AAT reduces autonomic arousal that simultaneously mirrors and facilitates further reductions in anxiety. Physiologically, interacting with a dog has been shown to lead to significant increases in β-endorphin, oxytocin, prolactin, phenylacetic acid (metabolite of β-phenylethylamine), and dopamine, as well as reductions in cortisol (Odendaal, 2000), all of which are associated with better emotional well-being. Interacting with a dog also significantly reduces the subjective distress of college students engaging in an expressive writing exercise about trauma (Hunt & Chizkov, 2014). Finally, previous research suggests that spending time with a therapy dog produces significant immediate reductions in anxiety and distress in college students, although these benefits disappear after a few weeks (Hunt, Gamarra, Reyes, Shearer, & Romeo, 2012). This suggests that time-limited opportunities to interact with a dog may reduce short-term anxiety but do not produce long-term benefits. Consequently, we chose interacting with a dog in a pleasant, study break group setting as a strong, active control for mindfulness meditation training.

Further research must also determine how long mindfulness programs must be in order to balance maximum effectiveness with efficiency (i.e., the optimal dose). Traditional MBSR programs include 26 hours of structured classes (8 two-and-a-half-hour long classes plus a 6-hr long retreat). This

substantial time commitment may not be feasible for many—in fact, those who face the most stressors may also be the most pressed for time. Moreover, in a recent review, Carmody and Baer (2009) found no significant relationship between number of class hours and effect sizes (*d*) on measures of psychological distress across different studies. This suggests that shorter programs may be just as effective and more realistic.

Finally, assessing the impact of mindfulness training programs can be somewhat challenging. All of the major self-report measures of mindfulness have disadvantages, and may be more or less relevant to specific populations (Bergomi, Tschacher, & Kupper, 2013). As with all self-report measures, they are subject to demand characteristics, and may have limited validity. Physiological measures of emotion regulation, on the other hand, may be a better index of enduring psychological change that results from mindfulness training. Heart rate variability (HRV) is one such measure.

HRV is the degree of fluctuation in the length of intervals between heartbeats. It reflects the relative ratio of parasympathetic and sympathetic nervous system (PNS and SNS) activity, and has been linked to both cardiovascular and mental health (Thayer, Ahs, Fredrikson, Sollers, & Wager, 2012). HRV is a physiological marker of the person's ability to regulate the stress response. HRV reflects the body's ability to respond to environmental challenges, as well as to self-regulate. Although somewhat counterintuitive, higher HRV can be more adaptive because it reflects the body's ability to self-regulate in response to stressful situations. That is, heart rate might increase in immediate response to perceived stressors, but someone with good stress management skills should be able to bring their heart rate back *down* quickly, resulting in greater HRV. Indeed, numerous studies have found an inverse relationship between HRV and perceived stress (e.g., Dishman et al., 2000), anxiety disorders (Licht, de Geus, van Dyck, & Penninx, 2009), and depression (Kemp, Quintana, Felmingham, Mathews, & Jelinek, 2012). In all such studies, higher HRV scores indicate reduced stress and distress and/or better stress management and coping.

Indeed, Burg, Wolf, and Michalak (2012) demonstrated that the ability to mindfully regulate one's attention is associated with higher HRV. Similarly, Mankus and colleagues (2013) examined the relationship between HRV and mindfulness in the context of generalized anxiety, and found that mindfulness was positively correlated with HRV. Moreover, mindfulness meditation training has also been shown to positively impact HRV. Krygier and colleagues (2013) showed that a 10 day, intensive Vipassana meditation retreat led to improvements in both subjective well-being and HRV. Garland, Gaylord, Boettiger, and Howard (2010) found that during alcohol cue exposure, alcohol-dependent adults who participated in a mindfulness training group had higher HRV than those

This document is copyrighted by the American Psychological Association or one of its allied publishers.
This article is intended solely for the personal use of the individual user and is not to be disseminated broadly.

who participated in an alcohol-dependence support group. They interpreted this as the mindfulness group's enhanced ability to utilize cognitive resources in the prefrontal areas of the brain to regulate their stress response in the face of distressing alcohol-related cues. This makes sense given that some of the same areas of the brain that regulate PNS and SNS activity, the central autonomic network, or CAN, also play a role in emotion regulation (Thayer & Lane, 2000). HRV, then, may be a particularly useful way of assessing the positive impact of mindfulness meditation on stress management and emotion regulation, as it is free from self-report biases that traditional pen and paper measures are subject to. Although HRV can hypothetically be biased by applying more voluntary effort or by trying to relax, participants were given *no* specific instructions to relax or practice breathing techniques (regardless of condition). Consequently, HRV was used as one of the primary outcome measures.

Specifically, the current study was designed to test the efficacy of a brief (four session) mindfulness meditation intervention as compared to an active control condition (interacting with a therapy dog) and a no-treatment control. Outcome measures included both self-reported changes in mindfulness, mood, and subjective well-being as well as psychophysiological data (HRV) collected during a stressful cognitive challenge specifically designed to mimic academic stressors frequently experienced by college students.

METHOD

Participants

Participants consisted of 74 undergraduate students recruited from psychology courses at the University of Pennsylvania who consented to participate in the study in exchange for extra credit in a psychology course. In the first phase, 26 students were enrolled in the study, with 24 completing the postgroup assessment. Thirteen were randomized to the mindfulness meditation group and 13 were randomized to the active control ('de-stress with dogs' group). In the second phase of the study, 48 students were enrolled, with 45 completing the postgroup assessment. Fourteen were randomized to the mindfulness meditation group, 12 were randomized to the dog group, and 22 were randomized to an additional no-treatment group. No-treatment controls completed the same baseline battery, weekly mood questionnaires, and postgroup assessment as the treatment groups but they did not participate in any group intervention. The participants were 57% female and 43% male, and 43% Asian, 41% Caucasian, 7% Hispanic, 3% African American, 3% other, 1% Native American, 1% Pacific Islander, and 1% unidentified.

This document is copyrighted by the American Psychological Association or one of its allied publishers.
This article is intended solely for the personal use of the individual user and is not to be disseminated broadly.

This document is copyrighted by the American Psychological Association or one of its allied publishers.
This article is intended solely for the personal use of the individual user and is not to be disseminated broadly.

Design Overview

Participants were randomly assigned, based on gender and order of enrollment, to either one of three conditions: 1) a 4-week mindfulness meditation intervention (i.e., the meditation group) or 2) a 4-week 'de-stress with dogs' group (i.e., the dog group) in which students interacted with a therapy dog and were provided with snacks and 3) a no-treatment control.

In the two treatment conditions, students attended four 1-hr long weekly groups in which they participated in their respective intervention, and then were asked to complete a brief mood questionnaire. The no-treatment group was simply asked to fill out a mood inventory online each week the same day as the group sessions. Prior to the first group session, all participants consented and completed a baseline battery of measures (described below) assessing mood, trait mindfulness, and demographic characteristics. In the two weeks following the last group session, all of the participants were required to come into the lab for a 1-hr long postgroup assessment. Each subject was administered select subtests of the Wechsler Adult Intelligence Scale (WAIS-IV) to provide an ecologically valid cognitive and social performance stressor while electrocardiogram (ECG) data were simultaneously collected. WAIS administrators were blind to condition and maintained a stony-faced, stern demeanor throughout. Following the WAIS, participants completed the same assessment battery they completed at baseline (minus the demographics).

Measures

The mood inventory consisted of the 20-item state anxiety subscale of the Spielberger State–Trait Anxiety Inventory (STAI-S; Spielberger, Gorsuch, & Lushene, 1970) followed by six select dysphoric affect items from the expanded form of the Positive and Negative Affect Schedule (PANAS-X; Watson & Clark, 1994). The STAI has been used in literally hundreds of studies with college students and has an alpha reliability coefficient of 0.92 (Spielberger, Gorsuch, Lushene, Vagg, & Jacobs, 1983). The six selected items from the PANAS included guilty, sad, distressed, ashamed, lonely and angry. Depressive symptoms were assessed using the Beck Depression Inventory (BDI-II; Beck, Steer, & Brown, 1996). The BDI is a 21-item, forced-choice questionnaire that measures depressive symptoms. Items are scored on a 0–3 scale and summed to produce a total score, with higher scores indicating more depressive symptoms. This measure has moderate to good internal consistency and good test–retest reliability and excellent diagnostic sensitivity.

Trait mindfulness was assessed at baseline and at the postgroup assessment via the 39-item Five Facet Mindfulness Questionnaire (FFMQ; Baer et al., 2006). The FFMQ measures five facets of mindfulness: observing, describing, acting with awareness, nonjudging of inner experience, and nonreactivity to inner experience. The five facets were found to have adequate to good alpha reliability coefficients (0.83, 0.91, 0.87, 0.87, and 0.75, respectively; Baer et al., 2006). The measure employs a five-point Likert scale, on which 1 = *never or very rarely true*, 2 = *rarely true*, 3 = *sometimes true*, 4 = *often true*, 5 = *very often or always true*).

HRV

ECG data were collected using a Vernier LabQuest® Mini and analyzed by Logger Lite software. HRV analysis was conducted using Kubios HRV version 2.1 developed by the Biosignal Analysis and Medical Imaging Group at the Department of Applied Physics, University of Eastern Finland, Kuopio, Finland. Three electrodes were attached to the participants' arms (one on right wrist, one at right elbow, one at left elbow) and remained during the entire administration of the WAIS subtests. That is, heart rate data were collected during the cognitive challenge, and not while people were actively meditating.

In the seminal review of HRV analysis, the recommended measure of HRV is the standard deviation of the normal-to-normal (SDNN) intervals (Task Force of the European Society of Cardiology the North American Society of Pacing Electrophysiology, 1996). The SDNN is now widely used among researchers as a measure of HRV. Although frequency domain variables hold promise as measures of sympatho-vagal balance or homeostatic reflexes, they are not well understood and there is no consensus in the field at this time as to how to interpret them (Billman, 2013). One systematic review of normal SDNN values in healthy individuals found a mean value of 50 ms and a median value of 51 ms (range 32 to 93 ms) from data gathered from 27 studies reporting SDNN values from short-term HRV analysis (Nunan, Sandercock, & Brodie, 2010). In addition, Medicore's Heart Rate Variability Analysis System notes that average SDNN above 50 indicates "high normal," which reflects good "ANS's regulat[ory] function and coping ability," with anything below 35 ms as poor with a "risk of developing stress induced disease" (Medicore, n.d., p. 27). In one study, significant differences in SDNN were found between rest conditions and during challenging tasks among normal college students. Specifically, the average HRV value for healthy individuals during a stressful office task was 35.40 ms, and the average during the rest period was 46.73 ms (Taelman, Vandeput, Vlemincx, Spaepen, & Van Huffel, 2011).

This document is copyrighted by the American Psychological Association or one of its allied publishers.
This article is intended solely for the personal use of the individual user and is not to be disseminated broadly.

Artifact Correction and Preprocessing

One obstacle to accurate HRV analysis is the possibility of errors in ECG data collection. Possible artifacts in QRS (which refer to the leftmost, middle, and right most splines that characterize ECG curves, respectively) detection can be divided into two categories: technical and physiological artifacts. Technical artifacts can include missing or additional QRS detections or errors in *interbeat interval* (hereafter referred to as RR) measurement. This can result from an error in the ECG recording software algorithm used to detect RR intervals, or from improperly fastened electrodes or excessive movement on the part of the subject. Possible physiological artifacts include ectopic beats (i.e., ventricular premature beats), common even in healthy subjects, or similar arrhythmic events (Peltola, 2012). Regardless of the source of error, artifact correction procedures are an important part of HRV analysis, without which results cannot be properly interpreted (Nunan et al., 2010).

Some of the most common methods of artifact correction include deletion of RR intervals, interpolation of degree zero, interpolation of degree one (linear interpolation), and cubic spline interpolation (Peltola, 2012). The last of these artifact correction methods is a popular method whereby data points are fitted to a third degree polynomial to produce smooth curves. Still, researchers have yet to arrive at a consensus for the best method of artifact correction when conducting HRV analysis.

In part because our participants were not able to lie prone and still during the WAIS stressor, we had a significant number of technical artifacts in our ECG data. We used the strong Kubios artifact correction option, which uses a threshold of 0.05 s to define abnormal deviation from the local mean RR interval. RR intervals selected by this criterion were corrected by a piecewise cubic spline interpolation method (Tarvainen & Niskanen, 2012). We also subjected the data to the "smoothin priors" correction to account for drift. Unfortunately, we were left with a few data points that still represented physiologically impossible values (e.g., SDNN raw scores of greater than 200). These data were clearly the result of technical artifacts. In these cases, the electrodes had fallen off the subjects while they were moving during the WAIS test. After eliminating these few outliers, the corrected and smoothed data were log transformed to normalize the distribution.

Procedure

The weekend before the first group session, all of the participants were consented electronically and were asked to complete the demographics

This document is copyrighted by the American Psychological Association or one of its allied publishers.
This article is intended solely for the personal use of the individual user and is not to be disseminated broadly.

questionnaire, the mood inventory, and the FFMQ. The group sessions for the dog and the mindfulness conditions were held at the same time during the same nights in separate rooms.

Destress With Dogs

The dog group was held in an open room containing a circle of chairs and a table with healthy snacks such as vegetables and hummus dip. Participants were brought into the room with the therapy dog and were instructed to interact with the dog and each other as they pleased. During several of the group meetings, the facilitator organized group "games" involving all participants in calling the dog and feeding her bits of popcorn. This condition was designed to be an ecologically valid intervention that mimics study breaks involving therapy dogs that are increasingly being implemented on college campuses (e.g., Schultheis, 2010; Wray, 2014). Cell phone use was prohibited.

Mindfulness Intervention

The mindfulness group was held in an open carpeted room down the hall from the dog condition. Participants were provided with yoga mats and blocks. The intervention, derived from the MBSR program created at the University of Massachusetts by John Kabat-Zinn, consisted of the following: breathing exercises, basic yoga including light stretching and balancing exercises, short (5 to 15 min) meditation sessions, and education about the physiology of the stress response. The overarching emphasis was on adopting a nonjudgmental attitude, particularly toward one's beliefs, feelings, or thoughts. After each session, participants in both treatment groups were given a paper-based copy of the mood inventory.

No-Treatment Control

Participants in the no-treatment group were sent links to the same questionnaire via e-mail during the same hour the groups were held. They were asked to complete their questionnaires online prior to the end of the day.

One to 2 weeks later, during their postgroup assessment, each subject was administered four subtests of the Wechsler Adult Intelligence Scale-IV (WAIS; Block Design, Similarities, Matrix Reasoning and Arithmetic; Wechsler, 2008) by an administrator who was unknown to

This document is copyrighted by the American Psychological Association or one of its allied publishers.
This article is intended solely for the personal use of the individual user and is not to be disseminated broadly.

This document is copyrighted by the American Psychological Association or one of its allied publishers.
This article is intended solely for the personal use of the individual user and is not to be disseminated broadly.

them and who was blind to group assignment. The WAIS is a challenging cognitive test that involves no feedback during standardized administration. The test itself can be experienced as a stressor for those who value intellectual capacity, and the administrators maintained a purposefully stern demeanor. Administration of this test was selected as a nondeceptive stressor and was meant to mimic stressful testing conditions that college students might encounter in their day-to-day lives. Throughout the test, ECG data were collected via three electrodes attached to the participants' arms. Following the WAIS, participants completed an assessment battery consisting of the mood inventory and the FFMQ. In the second phase, participants also completed the mood inventory prior to taking the cognitive challenge. On completion, participants were given course credit and debriefed.

RESULTS

There were no group differences at baseline in any of the measures, including depression, anxiety, dysphoric affect, mindfulness, or maladaptive perfectionism (all $F < 0.67$, *ns*). The nonjudging factor of the FFMQ was correlated with distress at baseline in the expected directions, such that people who were nonjudgmental were also less distressed (for depression, $r = -0.60$; for state anxiety, $r = -0.41$; for dysphoric affect, $r = -.54$, all $p < .001$).

Anxiety and Dysphoric Affect During Intervention

In a repeated-measures analysis of variance (ANOVA) looking at change over time in state anxiety, there was a significant effect of group, $F(2, 56) = 8.52$, $p = .001$. Planned comparisons showed that the control group was significantly different than both the dog and the mindfulness group, whereas the dog and mindfulness groups were not significantly different from each other overall. Further comparisons, however, revealed significant separation between the dog and mindfulness groups, with the mindfulness group showing significantly less state anxiety after the third and fourth classes, $t(47) = 1.98$, $p = .05$ and $t(46) = 2.21$ $p < .05$, respectively (see Figure 1).

Similarly, a second repeated-measures ANOVA predicting change over time in dysphoric affect as a function of group was also highly significant, $F(2, 56) = 7.55$, $p = .001$. Again, planned comparisons showed the control group to be significantly more dysphoric than both the

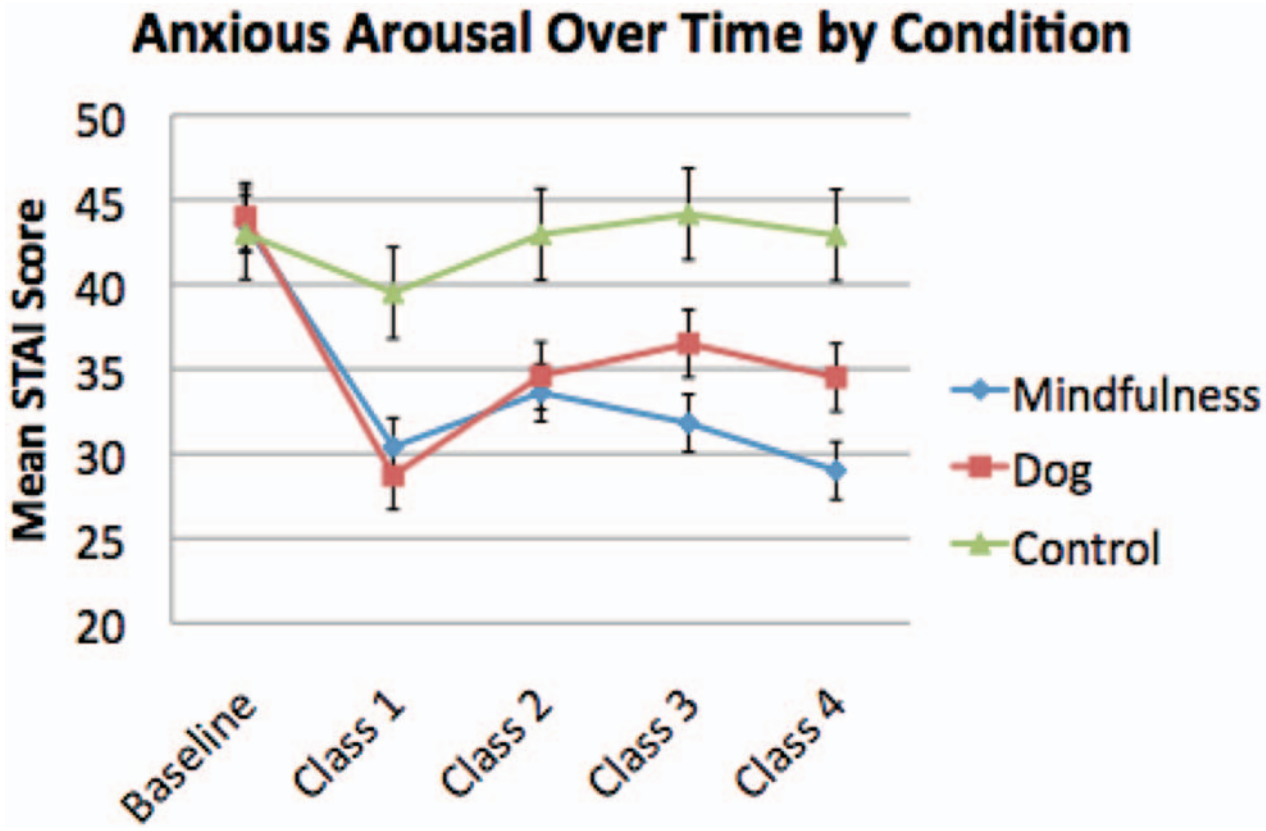

Figure 1. State anxiety over time by condition. See the online article for the color version of this figure.

dog and the mindfulness groups but the dog and mindfulness groups were not significantly different from each other at any time point (see Figure 2).

Contrary to expectations, mindfulness training did not lead to increases in self-reported trait mindfulness as measured by the FFMQ, and the overall ANOVA was not significant, $F(2, 61) = 0.3$, *ns*. It is interesting to note however, individuals in the dog group did show a significant increase in the nonjudging facet of the FFMQ, $t(22) = 2.98$, $p < .01$.

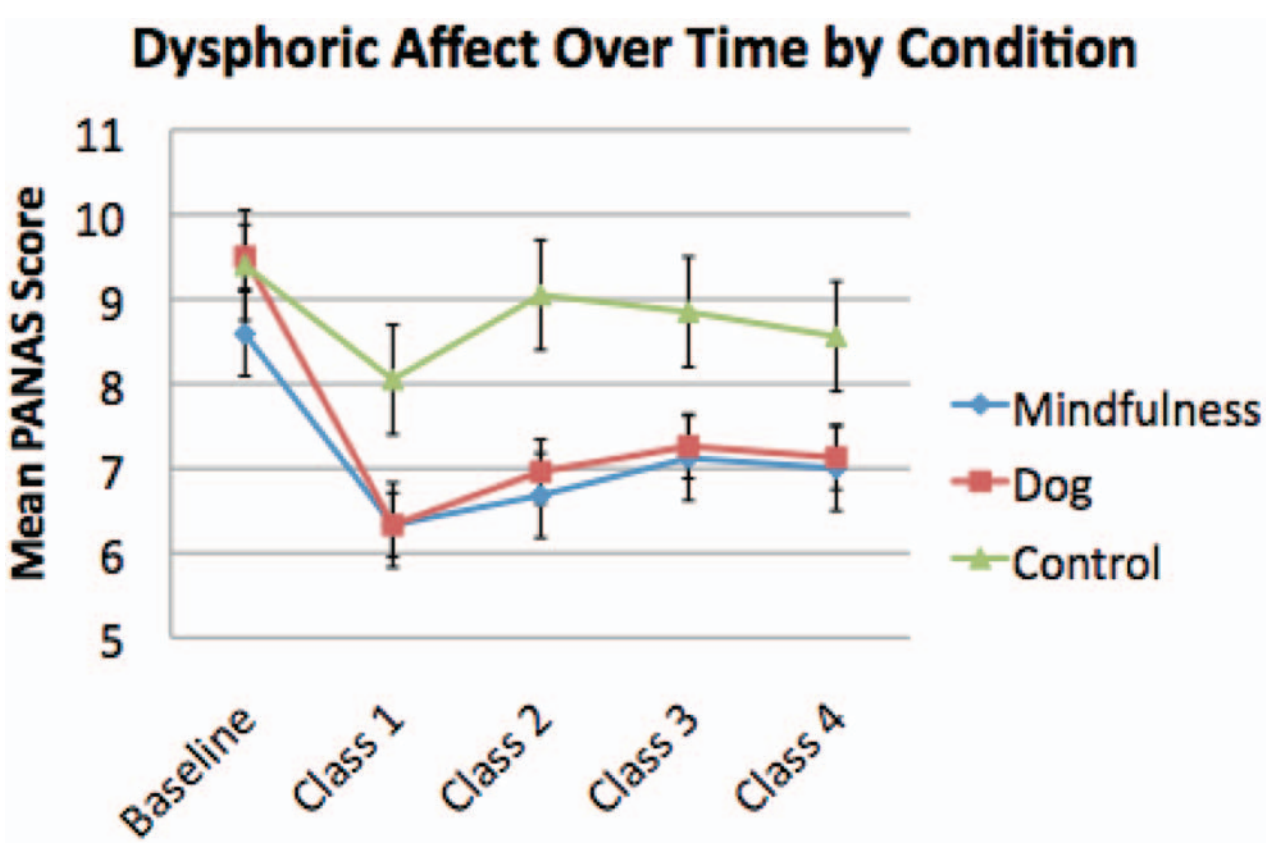

Figure 2. Dysphoric affect over time by condition. See the online article for the color version of this figure.

This document is copyrighted by the American Psychological Association or one of its allied publishers.
This article is intended solely for the personal use of the individual user and is not to be disseminated broadly.

Anxiety and Dysphoric Affect Prechallenge

One to 2 weeks after the intervention, there were nonsignificant trends in the data showing that the mindfulness group had maintained some of the gains relative to the other two groups in self-reported symptoms of anxiety and dysphoric affect. We conducted separate analyses of covariance (ANCOVAs) examining STAI and PANAS-X scores by condition controlling for baseline scores. In the case of STAI scores, the overall ANCOVA was not significant, $F(2, 37) = 2.24$, $p = .12$, but planned comparisons revealed marginally significant differences, with individuals in the mindfulness group being somewhat less anxious immediately prior to the challenge than individuals in the dog and control groups ($p = .06$ and $p = .10$, respectively). In the case of PANAS-X, the overall ANCOVA was also not significant, $F(2, 37) = 2.41$, $p = .10$, but planned comparisons revealed a significant difference between the mindfulness group and the control group, ($p < .05$) and no difference between the mindfulness and the dog groups. There were no significant differences in actual depressive symptoms as measured by the BDI-II (however, $F(2, 37) = 0.71$, *ns*). See Table 1 for means across all groups and time points.

Anxiety and Dysphoric Affect Postchallenge

The impact of group assignment on the ability to regulate mood during the cognitive stressor was mixed. Overall, there was no significant effect of group on self-reported postchallenge anxiety, controlling for prechallenge anxiety, $F(2, 39) = 1.28$, *ns*. It is interesting to note there was a significant effect of group on change in dysphoric affect, with individuals in the mindfulness group actually reporting greater increases in dysphoric affect compared with the other two groups, $F(2, 38) = 3.92$, $p < .05$. Post hoc paired samples *t* tests revealed that individuals in the mindfulness group reported significant increases in both state anxiety and dysphoric affect from pre- to postchallenge, $t(12) = 3.56$, $p < .01$ and $t(11) = 2.68$, $p < .05$, respectively. The dog group reported no change in dysphoric affect and a marginally significant increase in anxiety, $t(10) = 1.87$, $p = .09$. The control group reported a significant increase in dysphoric affect, $t(18) = 2.47$, $p < .05$. Across all of the groups, mean self-report scores of anxiety and dysphoric affect increased from pre- to postchallenge, even if only slightly.

HRV During Challenge

Of greater relevance to our hypotheses, however, we noted a highly significant difference in HRV across the groups with the mindfulness group showing

This document is copyrighted by the American Psychological Association or one of its allied publishers.
This article is intended solely for the personal use of the individual user and is not to be disseminated broadly.

Table 1. Means and Standard Deviations for All Outcome Variables Across Groups and Time

	Active treatment		Active control		Control	
Measure	*N*	*M* (*SD*)	*N*	*M* (*SD*)	*N*	*M* (*SD*)
FFMQ						
Pretreatment	27	125.52$_{a}$ (14.85)	26	121.85$_{a}$ (14.96)	17	125.24$_{a}$ (16.13)
Posttreatment	26	124.5$_{a}$ (16.68)	24	122.42$_{a}$ (15.98)	20	123.25$_{a}$ (12.49)
STAI						
Pretreatment	27	43.59$_{a}$ (10.31)	26	43.92$_{a}$ (10.23)	17	43$_{a}$ (11.26)
Group 1	27	30.37$_{a}$ (6.63)	24	28.67$_{a}$ (5.52)	20	39.50$_{b}$ (10.94)
Group 2	25	33.6$_{a}$ (8.63)	24	34.63$_{a}$ (7.29)	21	42.95$_{b}$ (11.99)
Group 3	26	31.85$_{a}$ (8.54)	23	36.5$_{b}$ (8.07)	20	44.15$_{c}$ (10.28)
Group 4	25	28.96$_{a}$ (7.37)	23	34.52$_{b}$ (10.00)	18	42.89$_{c}$ (10.27)
Posttreatment 1	14	35.64$_{a}$ (9.41)	11	40.82$_{a}$ (8.67)	20	39.95$_{a}$ (11.36)
Posttreatment 2	25	39.40$_{a}$ (10.71)	24	43.92$_{a}$ (12.22)	19	41.74$_{a}$ (12.91)
PANAS-X						
Pretreatment	27	8.59$_{a}$ (2.36)	26	9.50$_{a}$ (3.44)	17	9.41$_{a}$ (3.47)
Group 1	27	6.33$_{a}$ (.88)	24	6.33$_{a}$ (.64)	20	8.05$_{b}$ (2.26)
Group 2	25	6.68$_{a}$ (.85)	24	6.96$_{a}$ (1.37)	21	9.05$_{b}$ (2.94)
Group 3	26	7.12$_{a}$ (1.77)	23	7.26$_{a}$ (1.79)	20	8.85$_{b}$ (2.87)
Group 4	25	7$_{a}$ (2.58)	23	7.13$_{a}$ (1.84)	18	8.56$_{b}$ (2.75)
Posttreatment 1	14	6.64$_{a}$ (.75)	11	7.09$_{a,b}$ (1.45)	20	7.9$_{b}$ (2.25)
Posttreatment 2	24	7.5$_{a}$ (1.93)	24	7.92$_{a}$ (3.35)	19	8.58$_{a}$ (2.84)
BDI-II						
Pretreatment	14	10.36$_{a}$ (10.16)	12	11$_{a}$ (9.25)	17	10.12$_{a}$ (8.28)
Posttreatment	26	7.92$_{a}$ (8.09)	24	9.42$_{a}$ (10.86)	20	9.65$_{a}$ (6.9)
HRV (SDNN)	17	51.12$_{a}$ (14.84)	17	38.88$_{b}$ (16.30)	14	39.04$_{b}$ (13.57)

Note. Standard deviations appear in parentheses below means. Means with differing subscripts are significantly different at the $p < .05$ from other means in that row based on Fisher's LSD post hoc paired comparisons. FFMQ = Five Facet Mindfulness Questionnaire; STAI = State–Trait Anxiety Inventory; PANAS-X = Positive and Negative Affect Schedule (dysphoric affect items); BDI-II = Beck Depression Inventory; HRV = heart rate variability; SDNN = standard deviation of the normal-to-normal intervals.

significantly higher HRV than was shown by the other two groups, $F(2, 45) = 4.55$, $p < .05$ (see Figure 3, which shows raw SDNN scores, rather than log transformed scores, for ease of interpretation). In both cases, the effect size (as measured by Cohen's *d*) was large (mindfulness vs. no treatment control: $d = .85$) or very large (mindfulness vs. dog group: $d = 1.15$).

DISCUSSION

As expected, we found a significant effect on anxiety and dysphoric affect of both the mindfulness training and the active control (i.e., interacting with a therapy dog during a fun social study break) compared with a no-treatment control. Both active interventions resulted in identical short-term reductions in dysphoric affect. Over the course of 4 weeks, the mindfulness group showed a slight advantage in reducing state anxiety compared with

This document is copyrighted by the American Psychological Association or one of its allied publishers.
This article is intended solely for the personal use of the individual user and is not to be disseminated broadly.

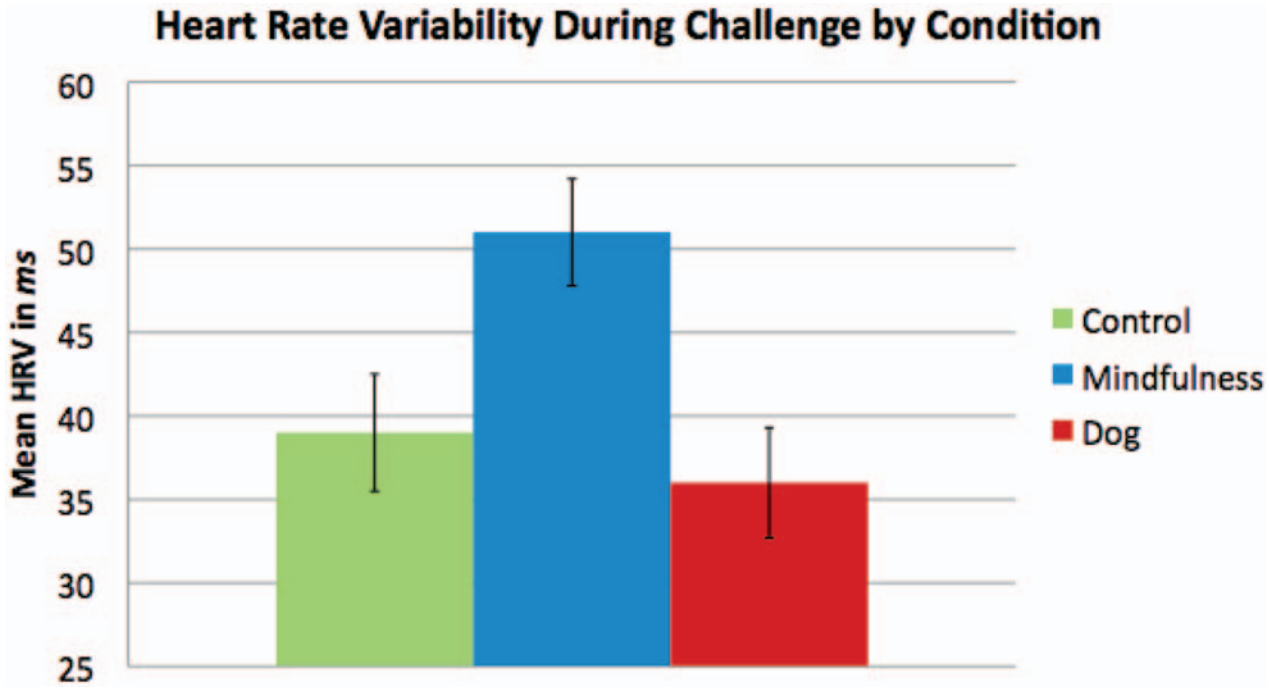

Figure 3. Mean heart rate variability during the cognitive challenge across condition. See the online article for the color version of this figure.

spending time with the dog in a similar group format, but interacting with the dog was also superior to no treatment. This suggests that interacting with a therapy dog in a social environment was a strong, active control for some of the active components of mindfulness training, including activity, attention, social interaction, and short-term anxiety and dysphoria reduction.

At the follow-up, all participants underwent a cognitive challenge stressor. There were few meaningful or significant differences in self-reported mood. Prior to the challenge, individuals in the mindfulness group showed slightly less anxiety and dysphoric affect than did the no treatment group and less anxiety than did the dog group. Postchallenge, all groups reported some increases in both anxiety and dysphoric affect, with the mindfulness group reporting slightly greater increases in dysphoric affect. These findings were all, at best, marginally significant. Even if reliable, however, the finding that individuals in the mindfulness group reported slightly more subjective distress is not necessarily surprising, given that mindfulness training encourages individuals to become attuned with whatever they are feeling or thinking, without trying to avoid or suppress any distressing sensations. Therefore, individuals in the mindfulness group may have simply been more aware of subjective changes they experienced in response to the stressor. Indeed, individuals who score high on measures of dispositional mindfulness also exhibit greater awareness of their own emotions and internal states (Baer et al., 2006; Brown & Ryan, 2003).

Despite the self-reported increases in negative affect however, the mindfulness group showed significantly higher HRV during the challenge. Because HRV is a physiological marker of a person's ability to regulate the stress response and given that the cognitive challenge was specifically designed to mimic the stressors frequently encountered by students at rigorous schools such as the University of Pennsylvania, this finding suggests that

This document is copyrighted by the American Psychological Association or one of its allied publishers.
This article is intended solely for the personal use of the individual user and is not to be disseminated broadly.

This document is copyrighted by the American Psychological Association or one of its allied publishers.
This article is intended solely for the personal use of the individual user and is not to be disseminated broadly.

a mindfulness intervention may help college students cope with academic stressors. Moreover, the positive effects of our intervention were evident despite the reduced the number of class hours compared with standard MBSR programs. This suggests that students, individuals characterized by significant time pressures, can still reap significant benefits from mindfulness practice without devoting exorbitant amounts of time and energy to it (which might paradoxically increase environmental stressors by increasing time pressures).

MBSR style programs are certainly not the only way to achieve stress reduction, or even to achieve measurable improvement in HRV. Other studies have also produced significant changes in HRV using a wide variety of other stress reduction techniques. For instance, one group of researchers found significant increases in HRV among depressed patients using HRV biofeedback (Karavidas et al., 2007). Other researchers found significant increases in normalized high-frequency HRV in a progressive muscle relaxation group compared with those in a control group (Dolbier & Rush, 2012). In addition, a yoga intervention was associated with an increases in SDNN compared with no-treatment control groups (Khattab, Khattab, Ortak, Richardt, & Bonnemeier, 2007). Although there may be multiple pathways to successful stress reduction, the goal of our study was to test whether MBSR in a shortened format might be one such intervention.

Findings for the impact of group assignment on self-reported trait mindfulness were surprising. Scores on these factors, collected via self-report questionnaires, showed only one significant change. Only a single facet of five identified by the FFMQ items (nonjudging) improved in the dog group. This may be an anomaly that will not replicate in future studies. On the other hand, it is possible that interacting with a dog actually did decrease self-judgment over time by delivering healthy doses of unconditional positive regard.

However, it is odd that the mindfulness training did not lead to increases in self-reported mindfulness, despite a significant group effect on HRV. In contrast, Burg and colleagues (2012) found that higher HRV was linked to increased mindfulness. One possible reason for this discrepancy might be deficiencies in available mindfulness measures. Past attempts at operationalizing mindfulness have been problematic (Chiesa, 2013). Some authors have even concluded, of past attempts, "none . . . seems to provide a comprehensive assessment of all aspects of mindfulness in samples from the general population" (Bergomi, Tschacher, & Kupper, 2013, p. 2). These authors closely inspected eight prominent currently available measures of mindfulness and found them each limited, including the FFMQ. Because of this uncertainty regarding available measures, we must interpret our results using the FFMQ with caution.

Limitations

Despite the significant findings of our study, there were some significant limitations. For instance, some of the HRV data were lost because of technical problems, such as failure to record, loose electrodes, or a significant proportion of artifacts in the data. Therefore, the sample of participants with viable HRV data was smaller (77%) than the overall pool of participants who completed the postgroup assessments. A complete sample would have given us greater confidence in our results. However, artifacts were evenly distributed across all groups, and it seems unlikely that trimming artifacts resulted in biased analyses.

It would have been better if we had collected baseline HRV data prior to participants taking the IQ test. This would have given us a measure of reactivity to the stressor and a measure of baseline physiological equilibrium. Unfortunately, we did not collect that data. Future studies by our group will certainly do so.

We were not able to demonstrate significant change in self-reported mindfulness as measured by the FFMQ. It may be that self-compassion would actually have been a better measure of change than the FFMQ. Indeed, research suggests that some of the positive outcomes resulting from mindfulness interventions can be better explained by changes in self-compassion. In particular, one group of researchers found that self-compassion is actually a better predictor of depressive symptoms, anxiety, quality of life, and well-being than is dispositional mindfulness (Van Dam, Sheppard, Forsyth, & Earleywine, 2011).

Self-report measures in general are vulnerable to social desirability and demand characteristics, and it is possible that the significant effects of the active interventions on self-reported anxiety and dysphoria were due to demand. This is one reason we used a highly face valid active control group. The fact that we still got statistically significant separation of self-reported anxious arousal between the MBSR group and the dog group seems to mitigate against the argument that demand characteristics accounted for the effects. Moreover, the fact that we got no significant changes on the FFMQ (which is obviously asking about skills and experiences taught in the MBSR intervention) again suggests that simple demand characteristics cannot account for our positive findings.

In addition, our sample consisted entirely of college students at the University of Pennsylvania. These findings may not be generalizable to other groups or even to other students. Our sample, like the University of Pennsylvania student body, was composed of equal percentages of Asian and Caucasian students, with some representation of Black, Latino/a, and other groups. This is neither nationally representative, nor representative of many

This document is copyrighted by the American Psychological Association or one of its allied publishers.
This article is intended solely for the personal use of the individual user and is not to be disseminated broadly.

This document is copyrighted by the American Psychological Association or one of its allied publishers.
This article is intended solely for the personal use of the individual user and is not to be disseminated broadly.

other schools. Although there were no effects of race in any of our analyses, it is possible that Asian students are particularly open to interventions like mindfulness that have their origins in Eastern cultures. In addition, college students may be more open-minded and liberal in general and thus, more receptive to mindfulness interventions than other groups of people. Future research should examine whether these findings can be extended to other populations.

Our follow-up period was quite short—between 1 and 3 weeks postassessment for all participants. Moreover, we did not track actual life stressors experienced by our participants and did not assess other relevant outcomes. Thus, we cannot draw conclusions about the long-term effects of such a brief intervention. Furthermore, we did not assess the degree to which participants were still practicing the skills long-term. Like any behavioral health intervention, MBSR is most useful if people continue to practice the skills in their normal lives after the formal intervention ends. However, we conceptualized this study as a preliminary investigation of whether a short MBSR style intervention would have any impact compared with an active control. Future studies should certainly include longer follow-up periods and more diverse outcome measures.

Finally, our intervention was based on the standard MBSR protocol, which includes psychoeducation, mindful stretching, and gentle yoga. It is possible that the positive impact of the intervention on HRV was due more to the effects of mild physical exercise than it was to the mindfulness meditation per se. Future studies will need to dismantle the potential contributions of exercise versus a mindfulness meditation component alone.

Implications

Despite these limitations, our study suggests that a short mindfulness training program can be a powerful yet simple way to help college students cope with stress and anxiety. Not only does practicing mindfulness meditation appear to significantly lower anxiety in the moment (even when compared with other effective stress-reducing activities such as spending time with a therapy dog), but it also appears to help students better regulate their stress response when confronted with cognitive challenges similar to those faced in a college environment. Moreover, interventions may not have to be as long as traditional MBSR programs to produce tangible benefits. We found as little as 4 weeks of mindfulness practice produced detectable physiological benefits. For students without a lot of time or money, mindfulness meditation could be a simple, cost-effective stress-management technique that does not require copious amounts of time to practice. Moreover, it is a skill that can

be done anytime, anywhere, in a group or alone, and is not burdened by a lengthy wait list like those potentially encountered at student mental health centers.

It is important to note that this study addressed a significant gap in the literature by using an active, ecologically valid control intervention. Much of previous mindfulness research has relied on waitlist control groups, which severely limited past studies methodologically. We showed that mindfulness significantly reduced subjective stress even when compared with an active, externally valid control intervention that is being widely implemented across college campuses, including at our home institution. We also used a physiological outcome measure administered during an ecologically valid stressor. Physiological measures such as HRV are not subject to demand characteristics and self-report bias and thus provide compelling evidence that an intervention has actually done something. Although it is possible to affect HRV by changing one's breathing intentionally (e.g., secondary to respiratory sinus arrhythmia), the ECG data were acquired during a stressful cognitive challenge, not during a focused breathing exercise. Moreover, it is unlikely that our subjects were simultaneously solving complex, abstract analogies and mental math problems while intentionally manipulating their breathing. It is hard to remain present in the face of a seemingly endless stream of impending deadlines and upcoming exams but perhaps learning how to do so could be the key to successfully navigating such obstacles. To our knowledge, mindfulness meditation is not a course required of any students but it might just be a lesson worth learning.

This document is copyrighted by the American Psychological Association or one of its allied publishers.
This article is intended solely for the personal use of the individual user and is not to be disseminated broadly.

REFERENCES

Aiken, J. (2014). Meet Monty. *Yale Law School Library News*. Retrieved from http://library.law.yale.edu/news/meet-monty

Allen, K. (2003). Are pets a healthy pleasure? The influence of pets on blood pressure. *Current Directions in Psychological Science, 12,* 236–239. http://dx.doi.org/10.1046/j.0963-7214.2003.01269.x

Baer, R. S. (2003). Mindfulness training as a clinical intervention: A conceptual and empirical review. *Clinical Psychology: Science and Practice, 10,* 125–143. http://dx.doi.org/10.1093/clipsy.bpg015

Baer, R. A., Smith, G. T., Hopkins, J., Krietemeyer, J., & Toney, L. (2006). Using self-report assessment methods to explore facets of mindfulness. *Assessment, 13,* 27–45. http://dx.doi.org/10.1177/1073191105283504

Banks, M. R., & Banks, W. A. (2002). The effects of animal-assisted therapy on loneliness in an elderly population in long-term care facilities. *The Journals of Gerontology: Series A: Biological Sciences and Medical Science, 57A,* M428–M432.

Barker, S. B., Pandurangi, A. K., & Best, A. M. (2003). Effects of animal-assisted therapy on patients' anxiety, fear, and depression before ECT. *The Journal of ECT, 19,* 38–44. http://dx.doi.org/10.1097/00124509-200303000-00008

Beck, A. T., Steer, R. A., & Brown, G. K. (1996). *Beck Depression Inventory—II manual*. San Antonio, TX: The Psychological Corporation.

Bergen-Cico, D., Possemato, K., & Cheon, S. (2013). Examining the efficacy of a brief mindfulness-based stress reduction (brief MBSR) program on psychological health. *Journal of American College Health, 61,* 348–360. http://dx.doi.org/10.1080/07448481.2013.813853
Berget, B., Ekeberg, O., & Braastad, B. O. (2008). Animal-assisted therapy with farm animals for persons with psychiatric disorders: Effects on self-efficacy, coping ability and quality of life, a randomized controlled trial. *Clinical Practice and Epidemiology in Mental Health, 4,* 9. http://dx.doi.org/10.1186/1745-0179-4-9
Bergomi, C., Tschacher, W., & Kupper, Z. (2013). The assessment of mindfulness with self-report measures: Existing scales and open issues. *Mindfulness, 4,* 191–202. http://dx.doi.org/10.1007/s12671-012-0110-9
Billman, G. E. (2013). The LF/HF ratio does not accurately measure cardiac sympatho-vagal balance. *Frontiers in Physics, 4,* 1–5.
Brougham, R. R., Zail, C. M., Mendoza, C. M., & Miller, J. R. (2009). Stress, sex differences, and coping strategies among college students. *Current Psychology, 28,* 85–97. http://dx.doi.org/10.1007/s12144-009-9047-0
Brown, K. W., & Ryan, R. M. (2003). The benefits of being present: Mindfulness and its role in psychological well-being. *Journal of Personality and Social Psychology, 84,* 822–848. http://dx.doi.org/10.1037/0022-3514.84.4.822
Burg, J. M., Wolf, O. T., & Michalak, J. (2012). Mindfulness as self-regulated attention: Associations with heart rate variability. *Swiss Journal of Psychology, 71,* 135–139. http://dx.doi.org/10.1024/1421-0185/a000080
Carmody, J., & Baer, R. A. (2009). How long does a mindfulness-based stress reduction program need to be? A review of class contact hours and effect sizes for psychological distress. *Journal of Clinical Psychology, 65,* 627–638. http://dx.doi.org/10.1002/jclp.20555
Chambless, D., & Ollendick, T. (2001). Empirically supported psychological interventions: Controversies and evidence. *Annual Review of Psychology, 52,* 685–716. http://dx.doi.org/10.1146/annurev.psych.52.1.685
Chiesa, A. (2013). The difficulty of defining mindfulness: Current thought and critical issues. *Mindfulness, 4,* 255–268. http://dx.doi.org/10.1007/s12671-012-0123-4
Collard, P., Avny, N., & Boniwell, I. (2008). Teaching mindfulness based cognitive therapy (MBCT) to students: The effects of MBCT on the levels of mindfulness and subjective well-being. *Counselling Psychology Quarterly, 21,* 323–336. http://dx.doi.org/10.1080/09515070802602112
Dishman, R. K., Nakamura, Y., Garcia, M. E., Thompson, R. W., Dunn, A. L., & Blair, S. N. (2000). Heart rate variability, trait anxiety, and perceived stress among physically fit men and women. *International Journal of Psychophysiology, 37,* 121–133. http://dx.doi.org/10.1016/S0167-8760(00)00085-4
Dolbier, C. L., & Rush, T. E. (2012). Efficacy of abbreviated progressive muscle relaxation in a high-stress college sample. *International Journal of Stress Management, 19,* 48–68. http://dx.doi.org/10.1037/a0027326
Falcone, A. (2014, September 6). Drexel becomes first university to host a permanent therapy dog in a recreation center. *Drexel Now*. Retrieved from http://drexel.edu/now/archive/2014/September/Drexel-Therapy-Dog/
Fine, A. H., & Beiler, P. F. (2008). Therapies and animals: Demystifying animal-assisted therapy. In A. L. Strozíer & J. E. Carpenter (Eds.), *Introduction to alternative and complementary therapies. Haworth practical practice in mental health* (pp. 223–246). New York, NY: The Haworth Press/Taylor and Francis Group.
Garland, E. L., Gaylord, S. A., Boettiger, C. A., & Howard, M. O. (2010). Mindfulness training modifies cognitive, affective, and physiological mechanisms implicated in alcohol dependence: Results of a randomized controlled pilot trial. *Journal of Psychoactive Drugs, 42,* 177–192. http://dx.doi.org/10.1080/02791072.2010.10400690
Grossman, P., Niemann, L., Schmidt, S., & Walach, H. (2004). Mindfulness-based stress reduction and health benefits: A meta-analysis. *Journal of Psychosomatic Research, 57,* 35–43. http://dx.doi.org/10.1016/S0022-3999(03)00573-7

This document is copyrighted by the American Psychological Association or one of its allied publishers.
This article is intended solely for the personal use of the individual user and is not to be disseminated broadly.

Hofmann, S. G., Sawyer, A. T., Witt, A. A., & Oh, D. (2010). The effect of mindfulness-based therapy on anxiety and depression: A meta-analytic review. *Journal of Consulting and Clinical Psychology, 78,* 169–183. http://dx.doi.org/10.1037/a0018555

Hollis-Walker, L., & Colosimo, K. (2011). Mindfulness, self-compassion, and happiness in non-meditators: A theoretical and empirical examination. *Personality and Individual Differences, 50,* 222–227. http://dx.doi.org/10.1016/j.paid.2010.09.033

Hunt, M., & Chizkov, R. R. (2014). Are therapy dogs like Xanax? Does animal-assisted therapy impact processes relevant to cognitive–behavioral psychotherapy? *Anthrozoös, 27,* 457–469.

Hunt, M., Gamarra, J., Reyes, A. R., Shearer, A., & Romeo, A. (2012, November). *Pet therapy for managing college stress*. Poster presented at the annual meeting of the Association of Behavioral and Cognitive Therapies, Baltimore, MD.

Jain, S., Shapiro, S. L., Swanick, S., Roesch, S. C., Mills, P. J., Bell, I., & Schwartz, G. E. R. (2007). A randomized controlled trial of mindfulness meditation versus relaxation training: Effects on distress, positive states of mind, rumination, and distraction. *Annals of Behavioral Medicine, 33,* 11–21. http://dx.doi.org/10.1207/s15324796abm3301_2

Kabat-Zinn, J. (1994). *Wherever you go, there you are: Mindfulness meditation in everyday life*. New York, NY: Hyperion Books.

Kabat-Zinn, J., Lipworth, L., & Burney, R. (1985). The clinical use of mindfulness meditation for the self-regulation of chronic pain. *Journal of Behavioral Medicine, 8,* 163–190. http://dx.doi.org/10.1007/BF00845519

Kabat-Zinn, J., Lipworth, L., Burney, R., & Sellers, W. (1986). Four year follow-up of a meditation-based program for the self-regulation of chronic pain: Treatment outcomes and compliance. *The Clinical Journal of Pain, 2,* 159–173. http://dx.doi.org/10.1097/00002508-198602030-00004

Kabat-Zinn, J., Massion, A. O., Kristeller, J., & Peterson, L. G. (1992). Effectiveness of a meditation-based stress reduction program in the treatment of anxiety disorders. *The American Journal of Psychiatry, 149,* 936–943. http://dx.doi.org/10.1176/ajp.149.7.936

Karavidas, M. K., Lehrer, P. M., Vaschillo, E., Vaschillo, B., Marin, H., Buyske, S., . . . Hassett, A. (2007). Preliminary results of an open label study of heart rate variability biofeedback for the treatment of major depression. *Applied Psychophysiology and Biofeedback, 32,* 19–30. http://dx.doi.org/10.1007/s10484-006-9029-z

Kemp, A. H., Quintana, D. S., Felmingham, K. L., Mathews, S., & Jelinek, H. F. (2012). Depression, comorbid anxiety disorders, and heart rate variability in physically healthy, unmedicated patients: Implications for cardiovascular risk. *PLoS ONE, 7,* e30777. http://dx.doi.org/10.1371/journal.pone.0030777

Kerr, S., Johnson, V. K., Gans, S. E., & Krumrine, J. (2004). Predicting adjustment during the transition to college: Alexithymia, perceived stress, and psychological symptoms. *Journal of College Student Development, 45,* 593–611. http://dx.doi.org/10.1353/csd.2004.0068

Khattab, K., Khattab, A. A., Ortak, J., Richardt, G., & Bonnemeier, H. (2007). Iyengar yoga increases cardiac parasympathetic nervous modulation among healthy yoga practitioners. *Evidence-Based Complementary and Alternative Medicine, 4,* 511–517.

Kruger, K. A., Trachtenberg, S. W., & Serpell, J. A. (2004). *Can animals help humans heal? Animal-assisted interventions in adolescent mental health*. Retrieved from http://www2.vet.upenn.edu/research/centers/cias/pdf/CIAS_AAI_white_paper.pdf

Krygier, J. R., Heathers, J. A. J., Shahrestani, S., Abbott, M., Gross, J. J., & Kemp, A. H. (2013). Mindfulness meditation, well-being, and heart rate variability: A preliminary investigation into the impact of intensive vipassana meditation. *International Journal of Psychophysiology, 89,* 305–313. http://dx.doi.org/10.1016/j.ijpsycho.2013.06.017

Ledesma, D., & Kumano, H. (2009). Mindfulness-based stress reduction and cancer: A meta-analysis. *Psycho-Oncology, 18,* 571–579. http://dx.doi.org/10.1002/pon.1400

Licht, C. M. M., de Geus, E. J. C., van Dyck, R., & Penninx, B. W. J. H. (2009). Association between anxiety disorders and heart rate variability in the Netherlands study of depression and anxiety (NESDA). *Psychosomatic Medicine, 71,* 508–518. http://dx.doi.org/10.1097/PSY.0b013e3181a292a6

MacCoon, D. G., Imel, Z. E., Rosenkranz, M. A., Sheftel, J. G., Weng, H. Y., Sullivan, J. C., . . . Lutz, A. (2012). The validation of an active control intervention for mindfulness based

This document is copyrighted by the American Psychological Association or one of its allied publishers.
This article is intended solely for the personal use of the individual user and is not to be disseminated broadly.

This document is copyrighted by the American Psychological Association or one of its allied publishers.
This article is intended solely for the personal use of the individual user and is not to be disseminated broadly.

stress reduction (MBSR). *Behaviour Research and Therapy, 50,* 3–12. http://dx.doi.org/10.1016/j.brat.2011.10.011

Mankus, A. M., Aldao, A., Kerns, C., Mayville, E. W., & Mennin, D. S. (2013). Mindfulness and heart rate variability in individuals with high and low generalized anxiety symptoms. *Behaviour Research and Therapy, 51,* 386–391. http://dx.doi.org/10.1016/j.brat.2013.03.005

Medicore. (n.d.). *SA-3000P clinical manual* (Version 3.0). Retrieved from http://medi-core.com/download/HRV_clinical_manual_ver3.0.pdf

Miller, J. J., Fletcher, K., & Kabat-Zinn, J. (1995). Three-year follow-up and clinical implications of a mindfulness meditation-based stress reduction intervention in the treatment of anxiety disorders. *General Hospital Psychiatry, 17,* 192–200. http://dx.doi.org/10.1016/0163-8343(95)00025-M

Misra, R., & McKean, M. (2000). College students' academic stress and its relation to their anxiety, time management, and leisure satisfaction. *American Journal of Health Studies, 16,* 41–51.

Nunan, D., Sandercock, G. R. H., & Brodie, D. A. (2010). A quantitative systematic review of normal values for short-term heart rate variability in healthy adults. *Pacing and Clinical Electrophysiology, 33,* 1407–1417. http://dx.doi.org/10.1111/j.1540-8159.2010.02841.x

Odendaal, J. S. J. (2000). Animal-assisted therapy: Magic or medicine? *Journal of Psychosomatic Research, 49,* 275–280. http://dx.doi.org/10.1016/S0022-3999(00)00183-5

Oman, D., Shapiro, S. L., Thoresen, C. E., Plante, T., & Flinders, T. (2008). Meditation lowers stress and supports forgiveness among college students: A randomized controlled trial. *Journal of American College Health, 56,* 569–578. http://dx.doi.org/10.3200/JACH.56.5.569-578

Peltola, M. A. (2012). Role of editing of R-R intervals in the analysis of heart rate variability. *Frontiers in Physics, 3,* 1–10.

Pritchard, M. E., Wilson, G. S., & Yamnitz, B. (2007). What predicts adjustment among college students? A longitudinal panel study. *Journal of American College Health, 56,* 15–22. http://dx.doi.org/10.3200/JACH.56.1.15-22

Sax, L. J. (1997). Health trends among college freshmen. *Journal of American College Health, 45,* 252–264. http://dx.doi.org/10.1080/07448481.1997.9936895

Schultheis, E. (2010). *Penn students go to the dogs to take a bite out of study stress*. Retrieved from http://articles.philly.com/2010-05-07/news/24958399_1_benefits-of-pet-therapy-dogs-penn-students

Shapiro, L., Brown, K. W., Thoresen, C., & Plante, T. G. (2011). The moderation of mindfulness-based stress reduction effects by trait mindfulness: Results from a randomized controlled trial. *Journal of Clinical Psychology, 67,* 267–277. http://dx.doi.org/10.1002/jclp.20761

Shapiro, S. L., Carlson, L. E., Astin, J. A., & Freedman, B. (2006). Mechanisms of mindfulness. *Journal of Clinical Psychology, 62,* 373–386. http://dx.doi.org/10.1002/jclp.20237

Shapiro, S. L., Schwartz, G. E., & Bonner, G. (1998). Effects of mindfulness-based stress reduction on medical and premedical students. *Journal of Behavioral Medicine, 21,* 581–599. http://dx.doi.org/10.1023/A:1018700829825

Somerville, J., Kruglikova, Y., Robertson, R. L., Hanson, L. M., & Maclin, O. H. (2008). Physiological responses by college students to a dog and a cat: Implications for pet therapy. *North American Journal of Psychology, 10,* 519–528.

Spielberger, C. D., Gorsuch, R. L., & Lushene, R. E. (1970). *Manual for the state-trait anxiety inventory*. Palo Alto, CA: Consulting Psychologists Press.

Spielberger, C. D., Gorsuch, R. L., Lushene, R., Vagg, P. R., & Jacobs, G. A. (1983). *Manual for the state-trait anxiety inventory*. Palo Alto, CA: Consulting Psychologists Press.

Taelman, J., Vandeput, S., Vlemincx, E., Spaepen, A., & Van Huffel, S. (2011). Instantaneous changes in heart rate regulation due to mental load in simulated office work. *European Journal of Applied Physiology, 111,* 1497–1505. http://dx.doi.org/10.1007/s00421-010-1776-0

Tarvainen, M. P., & Niskanen, J. (2012). *Kubios HRV version 2.1: User's guide*. Kuopio, Finland: Biosignal Analysis and Medical Imaging Group (BSAMIG), Department of Applied Physics, University of Eastern Finland.

Task Force of the European Society of Cardiology the North American Society of Pacing Electrophysiology. (1996). Heart rate variability: Standards of measurement, physiological interpretation and clinical use. *Circulation, 93,* 1043–1065. http://dx.doi.org/10.1161/01.CIR.93.5.1043

Thayer, J. F., Ahs, F., Fredrikson, M., Sollers, J. J., III, & Wager, T. D. (2012). A meta-analysis of heart rate variability and neuroimaging studies: Implications for heart rate variability as a marker of stress and health. *Neuroscience and Biobehavioral Reviews, 36,* 747–756. http://dx.doi.org/10.1016/j.neubiorev.2011.11.009

Thayer, J. F., & Lane, R. D. (2000). A model of neurovisceral integration in emotion regulation and dysregulation. *Journal of Affective Disorders, 61,* 201–216. http://dx.doi.org/10.1016/S0165-0327(00)00338-4

Toneatto, T., & Nguyen, L. (2007). Does mindfulness meditation improve anxiety and mood symptoms? A review of the controlled research. *The Canadian Journal of Psychiatry / La Revue canadienne de psychiatrie, 52,* 260–266.

Van Dam, N. T., Sheppard, S. C., Forsyth, J. P., & Earleywine, M. (2011). Self-compassion is a better predictor than mindfulness of symptom severity and quality of life in mixed anxiety and depression. *Journal of Anxiety Disorders, 25,* 123–130. http://dx.doi.org/10.1016/j.janxdis.2010.08.011

Vøllestad, J., Nielsen, M. B., & Nielsen, G. H. (2012). Mindfulness- and acceptance-based interventions for anxiety disorders: A systematic review and meta-analysis. *British Journal of Clinical Psychology, 51,* 239–260. http://dx.doi.org/10.1111/j.2044-8260.2011.02024.x

Watson, D., & Clark, L. A. (1994). *The PANAS-X: Manual for the positive and negative affect schedule-expanded form*. Iowa City, IA: Department of Psychology, University of Iowa.

Wechsler, D. (2008). *Wechsler Adult Intelligence Scale* (4th ed.). San Antonio, TX: Pearson.

Williams, J. M. G. (2010). Mindfulness and psychological process. *Emotion, 10,* 1–7. http://dx.doi.org/10.1037/a0018360

Wray, C. (2014). *Med school welcomes new therapy dog*. Retrieved from http://yaledailynews.com/blog/2014/01/24/med-school-welcomes-new-therapy-dog/

Received July 2, 2014
Revision received June 5, 2015
Accepted June 15, 2015 ■

This document is copyrighted by the American Psychological Association or one of its allied publishers.
This article is intended solely for the personal use of the individual user and is not to be disseminated broadly.
