# Supplementary material for: Use of a Smartphone-Based App in an Online Behavioral Neuroscience Lab Course
Source: J Undergrad Neurosci Educ. 2026 Jul 1;24(2):34–43. doi: 10.59390/001c.163102 (PMC13625900; doi:10.59390/001c.163102)
Supplement: Supplementary file 2 [file junejournal_2026_24_2_163102_348898.pdf]

Article

The Influence of Slow-Paced Breathing on Executive Function

S. Laborde[1,2], M. S. Allen[3], U. Borges[1,4], T. J. Hosang[5], P. Furley[6], E. Mosley[7], and F. Dosseville[8,9]

[1]Department of Performance Psychology, Institute of Psychology, German Sport University Cologne, Germany
[2]UFR STAPS, EA 4260 CESAMS, University of Caen Normandy, Caen, France
[3]School of Psychology, University of Wollongong, Australia
[4]Department of Health & Social Psychology, Institute of Psychology, German Sport University Cologne, Germany
[5]Experimental Psychology Unit, Helmut Schmidt University/University of the Federal Armed Forces, Hamburg, Germany
[6]Institute for Training and Computer Science in Sport, German Sport University Cologne, Germany
[7]Department of Sport Science and Performance, Solent University, Southampton, UK
[8]UMR-S 1075 COMETE, Caen, France
[9]INSERM, UMR-S 1075 COMETE, University of Caen Normandy, Caen, France

This document is copyrighted by the American Psychological Association or one of its allied publishers.
This article is intended solely for the personal use of the individual user and is not to be disseminated broadly.

Abstract: The aim of this experiment was to test the immediate effects of slow-paced breathing on executive function. Slow-paced breathing is suggested to increase cardiac vagal activity, and the neurovisceral integration model predicts that higher cardiac vagal activity leads to better executive functioning. In total, 78 participants (41 men, 37 women; M_{age} = 23.22 years) took part in two counterbalanced experimental conditions: a 3 × 5 min slow-paced breathing condition and a television viewing control condition. After each condition, heart rate variability was measured and participants performed three executive function tasks: the color-word match Stroop (inhibition), the automated operation span task (working memory), and the modified card sorting task (cognitive flexibility). Results showed that performance on executive function tasks was better after slow-paced breathing compared to control, with higher scores observed for Stroop interference accuracy, automated operation span score, and perseverative errors, but not Stroop interference reaction times. This difference in executive function between experimental conditions was not mediated by cardiac vagal activity. Therefore, findings only partially align with predictions of the neurovisceral integration model. Slow-paced breathing appears a promising technique to improve immediate executive function performance. Further studies are recommended that address possible alternative underlying mechanisms and long-term effects.

Keywords: heart rate variability, performance, neurovisceral integration model, parasympathetic nervous system, vagus nerve

Improving executive functions is a constant endeavor for humans across the lifespan (Diamond & Ling, 2016), from children (Takacs & Kassai, 2019) to older adults (Nguyen et al., 2019). Of interest are both the short-term immediate effects, as well as long-term effects. Based on the physiological mechanisms suggested to underlie slow-paced breathing (Gerritsen & Band, 2018; Lehrer & Gevirtz, 2014; Mather & Thayer, 2018; Noble & Hochman, 2019) and on the predictions outlined in the neurovisceral integration model (Smith et al., 2017; Thayer et al., 2009), the current study aimed to test the immediate effects of a relaxation technique – slow-paced breathing – on the three core executive functions (Diamond, 2013): inhibition, working memory, and cognitive flexibility.

According to the neurovisceral integration model (Smith et al., 2017; Thayer et al., 2009), the effectiveness of the executive functioning of the prefrontal cortex can be indexed via heart rate variability (HRV), the time variation between two successive R peaks (Berntson et al., 1997; Laborde, Mosley, et al., 2017; Malik et al., 1996). More specifically, executive functioning is suggested to be indexed via the HRV parameters reflecting cardiac vagal activity – the activity of the vagus nerve regulating cardiac functioning (Thayer et al., 2009). The vagus nerve is the main nerve of the parasympathetic nervous system, and plays an essential role in self-regulation (Brodal, 2016; Thayer et al., 2009; Thayer & Lane, 2009). In the time-domain, one main HRV parameter reflecting cardiac vagal activity is the root mean square of the successive differences (RMSSD; Berntson et al., 1997; Laborde, Mosley, et al., 2017; Malik et al., 1996). In the frequency-domain, the physiological interpretation of HRV depends on the breathing frequency. When breathing frequency is comprised between 9 and 24 cycles per minute, cardiac vagal activity is reflected in high-frequency HRV. However, when breathing frequency is decreased under 9 cpm, like it is done with slow-paced breathing, the reflection of cardiac vagal activity is then shifted to low-frequency HRV, as it

© 2021 Hogrefe Publishing

https://doi.org/10.1027/0269-8803/a000279

This document is copyrighted by the American Psychological Association or one of its allied publishers.
This article is intended solely for the personal use of the individual user and is not to be disseminated broadly.

has been evidenced by a recent blockade study (Kromenacker et al., 2018). The relationship between cardiac vagal activity and executive functioning originates from the common structures and networks involved in cardiac and cognitive regulation (Thayer et al., 2012). The effectiveness of executive functioning in the prefrontal cortex is supported via the optimal activation of neural networks, underlined with a flow of activity along neural pathways enabling to establish adequate mappings between input, internal states, and outputs needed to perform a given task (Miller & Cohen, 2001), leading to flexible responses to changing environments (Smith et al., 2017; Thayer et al., 2009). The neurovisceral integration model assumes a linear relationship between cardiac vagal activity and executive performance. That is, a higher cardiac vagal activity will be associated with a higher executive performance.

Various methods have been proposed to increase cardiac vagal activity (Laborde, Mosley, & Mertgen, 2018; Laborde, Mosley, & Ueberholz, 2018), among which slow-paced breathing (Gerritsen & Band, 2018; Lehrer & Gevirtz, 2014; Mather & Thayer, 2018; Noble & Hochman, 2019). Slow-paced breathing is a breathing technique where the inhalation and exhalation durations are controlled ("paced"), and where breathing is performed at a slower pace (around 6 cycles per minute) than spontaneous breathing, which is usually between 12 and 20 cycles per minute in adults (Sherwood, 2006). The most common way to realize the breathing pacing is via visual stimuli (e.g., Allen & Friedman, 2012; Laborde, Allen, et al., 2017; Tsai et al., 2015), while auditive or kinesthetic methods (e.g., vibrations) have been used less frequently. Several theoretical perspectives coexist regarding the mechanisms underlying slow-paced breathing, that all mention a common aspect to explain the influence of slow-paced breathing on self-regulation and executive functions, which is the involvement of the vagus nerve (Gerritsen & Band, 2018; Lehrer & Gevirtz, 2014; Mather & Thayer, 2018; Noble & Hochman, 2019). Specifically, slow-paced breathing is suggested to influence vagal afferents via its action on the baroreflex and on pulmonary afferents. These vagal afferents are then suggested to input to the central autonomic network (Gerritsen & Band, 2018; Mather & Thayer, 2018; Noble & Hochman, 2019), which output is cardiac vagal activity, the core of the neurovisceral integration model (Smith et al., 2017; Thayer et al., 2009). Empirically, both immediate (Laborde, Allen, et al., 2017; Lewis et al., 2015; Szulczewski & Rynkiewicz, 2018; Wells et al., 2012; You et al., 2021) and long-term (Laborde, Hosang, et al., 2019) effects of slow-paced breathing on cardiac vagal activity have been documented in previous research. However, less is known about how slow-paced breathing might affect executive functioning.

The three core executive functions – on which higher-order executive functions such as decision making, problem solving and planning are built – are inhibition, working memory, and cognitive flexibility (Diamond, 2013; Miyake & Friedman, 2012; Miyake et al., 2000). Inhibition reflects being able to control attention, behavior, thoughts, and/or emotions to override a strong impulse, and to do instead what is more appropriate according to the context (Diamond, 2013). A classical test for inhibition is the color word Stroop test (Stroop, 1935), where participants are requested to read out the color in which a word is printed while ignoring the meaning of the word. In the congruent condition the color matches the meaning of the word (e.g., the word "blue" expressed in the color blue), while in the incongruent condition the color differs from the meaning of the word (e.g., the word "blue" expressed in the color red). The incongruent condition requires participants to inhibit the prepotent response of reading a word. Both the speed and accuracy of the responses can be measured. However, inhibition is primarily reflected as accuracy (error rate; McDowd et al., 1995), as it captures the ability to temporarily maintain the task goal in a retrievable state (Kane & Engle, 2003).

Previous research has found that Stroop task performance relates to cardiac vagal activity. A negative relationship has been observed between resting cardiac vagal activity and reaction times on incongruent and threat words (Johnsen et al., 2003), whereas a positive relationship has been observed between resting cardiac vagal activity and Stroop accuracy (i.e., Stroop interference score; Albinet et al., 2016). These two findings are in line with the neurovisceral integration model (Thayer et al., 2009). One study observed mixed-findings between resting cardiac vagal activity (assessed with high-frequency HRV) and Stroop accuracy (i.e., Stroop interference score; Subramanya & Telles, 2015). However, the experimental manipulation (meditation) occurring before the resting measurement might have introduced some confounding effects regarding the interpretation of high-frequency HRV, given that it is supposed to reflect cardiac vagal activity only when respiratory frequency is comprised between 9 and 24 cycles per minute (Berntson et al., 1997; Malik et al., 1996). As respiratory frequency was not assessed in the study, it is not possible to draw firm conclusions about cardiac vagal activity. The current experiment will investigate both accuracy and reaction time for the Stroop task controlling for respiratory frequency.

Working memory involves working with information no longer perceptually present (Baddeley & Hitch, 1994). Stated differently, working memory involves holding information in mind and mentally working with it (Diamond, 2013). A classical test to assess working memory capacity is the automated operation span task (AOSPAN; Unsworth

 © 2021 Hogrefe Publishing

This document is copyrighted by the American Psychological Association or one of its allied publishers.
This article is intended solely for the personal use of the individual user and is not to be disseminated broadly.

et al., 2005). The AOSPAN task requires participants to solve mathematics problems while holding a number of unrelated letters in memory. Previous research has found a positive relationship between resting cardiac vagal activity and AOSPAN performance (Laborde et al., 2015), and a negative relationship between task cardiac vagal activity (i.e., cardiac vagal activity measured during the AOSPAN) and AOSPAN performance when the task is realized under high pressure (Mosley et al., 2018), which might reflect the adaptation of cardiac vagal activity to the demands of the situation (Mosley et al., 2018). Positive relationships between resting cardiac vagal activity and other tasks that reflect working memory have also been reported in the literature (Hansen et al., 2003, 2004, 2009; Morandi et al., 2019; Pu et al., 2010; Sebastiani et al., 2019).

Cognitive flexibility builds on inhibition and working memory (Davidson et al., 2006; Diamond, 2013). Cognitive flexibility involves being able to change perspective, in particular, spatially or interpersonally (Diamond, 2013). To change perspective, there is the need to inhibit a previous perspective, and "load" a new perspective into working memory. A classical way to investigate cognitive flexibility is via the Wisconsin card sorting task (WCST; Milner, 1982; Stuss et al., 2000). In this test, each card can be sorted by color, shape, or number. Participants have to deduce the correct sorting criterion on the basis of the feedback they receive, and adapt to the new sorting rule as fast as they receive feedback that the sorting rule has changed. Previous research has shown a positive relationship between resting cardiac vagal activity and performance on the WCST (i.e., negative relationship with decision errors; Albinet et al., 2010; Hovland et al., 2012; Mathewson et al., 2012), and this is similar to what has been found in other tasks of cognitive flexibility (Alba et al., 2019; Colzato et al., 2018).

Although the influence of diverse breathing techniques on cognition has been considered in previous research (Gothe et al., 2013; Shannahoff-Khalsa et al., 1991; Yadav & Mutha, 2016), the influence of slow-paced breathing on executive functioning has received little attention to date. So far, the focus has been on inhibition (Laborde, Lentes, et al., 2019; Prinsloo et al., 2011), working memory (Bonomini et al., 2020; Prinsloo et al., 2011), cognitive flexibility (Bonomini et al., 2020), and decision making (De Couck et al., 2019). Decision making does not belong to the core executive functions but is considered a higher-order executive function that relies on core executive functions (Diamond, 2013). Overall, accuracy in inhibition (Laborde, Lentes, et al., 2019), working memory (Bonomini et al., 2020; Prinsloo et al., 2011), and decision making (De Couck et al., 2019) was found to be improved, while no influence was found on inhibition in Prinsloo et al. (2011). Processing speed remained unchanged (Bonomini et al., 2020; Laborde, Lentes, et al., 2019). Overall, research investigating the effects of slow-paced breathing on the three core executive functions is limited (with available studies characterized by some methodological shortcomings, such as the absence of an appropriate marker for cardiac vagal activity (De Couck et al., 2019; Prinsloo et al., 2011), unclear duration and/or characteristics of slow-paced breathing (Bonomini et al., 2020; De Couck et al., 2019), missing solid theoretical background (De Couck et al., 2019; Prinsloo et al., 2011), or investigated the relationship between slow-paced breathing and executive function after physical exercise (Laborde, Lentes, et al., 2019).

In summary, this experiment tests predictions of the neurovisceral integration model (Thayer et al., 2009) to investigate the immediate effects of slow-paced breathing on executive performance via an increase in cardiac vagal activity (Gerritsen & Band, 2018; Lehrer & Gevirtz, 2014; Mather & Thayer, 2018; Noble & Hochman, 2019). A within-subject design was selected given the large intra-individual variability in HRV (Laborde, Mosley, et al., 2017; Quintana & Heathers, 2014). Regarding the effects of the experimental manipulation, we hypothesized that during the experimental manipulation (DURING), in comparison to the control (CON) condition (where participants will be breathing spontaneously and watching an emotionally-neutral TV documentary), at the physiological level (Hypothesis 1, H1) in the slow-paced breathing (SPB) condition participants will display a higher cardiac vagal activity (operationalized via RMSSD), a lower HR, and a lower respiratory frequency. We assume that similar physiological changes will still be observed after the experimental manipulation (POST). We further hypothesized that executive performance will be higher in the SPB condition in comparison to the CON condition (Hypothesis 2, H2), and that this difference will be mediated via cardiac vagal activity (Hypothesis 3, H3).

Method

Participants

To determine our sample size, we explored effect sizes presented in previous research of slow-paced breathing and executive functioning (De Couck et al., 2019; Prinsloo et al., 2011). We computed an a priori power analysis using the software G*Power 3.0 (Faul et al., 2009). A medium effect size (f = .25), for a repeated-measures MANOVA (within-factors effects), with statistical power set at .80 and an α level of .05, requires a total sample size of N = 66. We recruited a larger sample of 90 participants to allow for potential dropout or technical issues with data collection.

© 2021 Hogrefe Publishing

This document is copyrighted by the American Psychological Association or one of its allied publishers.
This article is intended solely for the personal use of the individual user and is not to be disseminated broadly.

Exclusion criteria were self-reported cardiovascular conditions, and other chronic conditions that could influence breathing or HR patterns, such as asthma, diabetes, and neurological conditions (Laborde, Mosley, et al., 2017). Because of technical issues (excessive noise or artefacts on the ECG signal) 12 participants were excluded and the final sample was comprised of $N = 78$ (41 men, 37 women; M_{age} = 23.22 years; age range = 18–30 years; body mass index (BMI) = 22.40 ± 2.23; waist-to-hip ratio = 0.80 ± 0.05). None of the participants were smokers. This sample size elevated statistical power to .86 with all other parameters held constant. All participants gave written informed consent before participation and were informed that they could withdraw from the study at any time without explanation. The experiment was conducted in line with the Declaration of Helsinki, and the protocol was approved by a human research ethics committee at the German Sport University Cologne (Project identification code 42/2015).

Material and Measures

Cardiac Vagal Activity

An ECG device was used to measure HRV (Faros 180°, Bittium, Kuopio, Finland). The sampling rate was 500 Hz. Two disposable ECG pre-gelled electrodes were used (Ambu L-00-S/25, Ambu GmbH, Bad Nauheim, Germany). The negative electrode was placed in the right infraclavicular fossa (just below the right clavicle) and the positive electrode was placed on the left side of the chest, below the pectoral muscle in the left anterior axillary line. The full ECG recording was inspected visually, and artefacts were corrected manually (Laborde, Mosley, et al., 2017). From ECG recording we extracted RMSSD using the software Kubios (University of Eastern Finland, Kuopio, Finland). RMSSD was chosen to operationalize cardiac vagal activity as it is less affected by respiration (Hill et al., 2009). However, given the current debate regarding whether or not to control for respiratory parameters when assessing HRV (Grossman et al., 1991; Grossman & Kollai, 1993; Laborde, Mosley, et al., 2017; Larsen et al., 2010; Thayer et al., 2011), respiratory frequency was also calculated in order to better understand whether potential changes in RMSSD are related to cardiac vagal activity or are affected by changes in respiratory frequency. Respiratory frequency was computed via the ECG derived respiration algorithm of Kubios (Tarvainen et al., 2014).

Slow-Paced Breathing Exercise

Similar to previous research (Laborde, Allen, et al., 2017), the slow-paced breathing exercise was realized with the help of a video showing a ball moving up and down at the rate of six cycles per minute. The participants had to inhale continuously through the nose while the ball was going up, and exhale continuously with pursed lips when the ball was going down. This was a video capture of the software EZ-Air Plus (Biofeedback Federation of Europe[1]). The video displayed a 3 × 5 min slow-paced breathing exercise, with a 1-min break between each 5 minute slow-paced breathing unit, corresponding to a total of 17 minutes. The 1 minute break between each slow-paced breathing unit was introduced as some participants reported in a pilot study that 15 minutes of non-stop slow-paced breathing was very demanding. Exhalation (5.5 s) lasted slightly longer than inhalation (4.5 s) as prolonged exhalation contributes to larger beat-to-beat heart fluctuations compared to a prolonged inhalation, and therefore induces a higher cardiac vagal activity (Strauss-Blasche et al., 2000; Van Diest et al., 2014).

A familiarization period for slow-paced breathing was created in order for participants to become familiar with the technique. Inhaling via the nose (i.e., nasal breathing) is important because the air is warmer, cleaner and more humid (Lorig, 2011). In addition, nasal airflow was found to provoke respiratory oscillations leading to synchronized electrical activity in the piriform (olfactory) cortex, as well as in limbic-related brain areas, including amygdala and hippocampus. Therefore, inhaling through the nose is suggested to provoke optimal activation of neural networks linked to stimulus processing and behavior (Biskamp et al., 2017; Heck et al., 2019; Maric et al., 2020; Noble et al., 2017; Perl et al., 2019; Tort et al., 2018; Zaccaro et al., 2018; Zelano et al., 2016).

Exhaling takes place via the mouth, which offers less ventilatory resistance than the nasal channel (Lorig, 2011). Moreover, exhalation is realized via pursed-lips, which offers greater control over the flow of air, enabling participants to match it precisely to the exhalation duration, and which helps to reduce respiratory rate (Fagevik Olsen et al., 2015; Mayer et al., 2018; Noble & Hochman, 2019). Participants were then asked to put one hand on their chest and one hand on their stomach and were given the following instructions: "The hand on the chest should not move, only the hand on the belly should move: The belly should get bigger during the inhalation phase, and smaller during the exhalation phase." This instruction reflects an optimal activation of the diaphragm. When the diaphragm contracts and goes down, it increases the volume of the thoracic cavity and creates an area of low pressure that causes air to flow into the lungs to equalize the pressure (Lorig, 2011). During spontaneous breathing exhalation is mostly passive. However, in slow-paced breathing the forced exhalation can involve abdominal muscles which, via their

[1] https://bfe.org/new/try-our-breath-pacer-ez-air-plus/

 © 2021 Hogrefe Publishing

This document is copyrighted by the American Psychological Association or one of its allied publishers.
This article is intended solely for the personal use of the individual user and is not to be disseminated broadly.

contraction, help to push the diaphragm back up to the thorax, and consequently push out additional air (Lorig, 2011; West, 2015; West & Luks, 2016). Finally, the diaphragm movements may also contribute to trigger the neural oscillations observed with slow-paced breathing (Bordoni et al., 2018), which are expected to play a role in emotion regulation and cognitive control (Mather & Thayer, 2018; Tort et al., 2018).

During the familiarization, the breathing frequency is progressively decreased with 2 min units to 10 cycles per minute, 8 cycles per minute, and then 6 cycles per minute, with a 1 minute break between each unit. The slow-paced breathing technique requires the participant to breathe in and breathe out continuously and uniformly when the ball goes up and down respectively. When this instruction is correctly followed, the sine-waves oscillation characteristics of slow-paced breathing can be observed in the R-R tachogram (Lehrer & Gevirtz, 2014). The experimenter verified whether the participant was realizing the slow-paced breathing technique correctly during the familiarization before moving on to the next step of the experiment.

TV Neutral Documentary (Control Condition)

To serve as a control condition, a TV documentary ("*Abenteuer Forschung*" [Research Adventures]) about research discoveries related to space and the universe was shown to participants for the same duration as the slow-paced breathing familiarization exercise. This TV documentary was found to be subjectively emotionally neutral in a pilot study prior to the experiment.

Executive Function Tasks

Inhibition Task

As a measure of inhibition, we used the computerized version of the color word match Stroop task (Stroop, 1935) with verbal responding available in the Inquisit library,[2] and ran it with the Inquisit software (version 5; Millisecond Software, 2016). Words appeared in 28-pt Arial font in the middle of a white screen. Three stimuli were used: colored square (*congruent control* stimuli), colored words displayed with the color corresponding to the word (*congruent* stimuli, e.g., the word "blue" is displayed in blue color). Colored words displayed with a color not corresponding to the word (*incongruent* stimuli, e.g., the word "blue" is displayed in red color). Participants were asked to name the color in which the word was written as fast and as accurately as possible, while ignoring the written meaning of the word. A headset (Sennheiser PC 8, Wedemark, Germany) was placed on their head for stability, with a microphone directly in front of their mouth to record the answers. The familiarization included 20 trials whereas for the main assessment participants completed 84 trials: 4 colors (red, green, blue, black) × 3 color stimulus congruency (congruent, incongruent, control squares) × 7 repetitions. The stimuli remained on the screen until response and latencies were measured from onset of stimuli. The inter-trial interval was 200 ms, and the error feedback (a red cross) was 400 ms.

Working Memory

As a measure of working memory capacity, we used the AOSPAN (Unsworth et al., 2005) which is based on the original operation span task (Turner & Engle, 1989). We used the version of the task programmed within the Inquisit database.[3] In this task participants are required to solve mathematics problems while trying to remember an unrelated set of letters. The task included a total of 15 trials (three trials each with 3, 4, 5, 6, and 7 letters). An example of a three item trial is $(8/2) - 1 = 1$? (correct/incorrect?) → F; is $(6 + 1) + 2 = 8$? (correct/incorrect?) → P; is $(10 + 2) - 5 = 15$? (correct/incorrect?) → Q. After completing the three questions in this example, participants were asked to select the presented letters with a mouse click from an array of 12 potential letters in the order that they were presented (in this case F, then P, then Q). A familiarization to the task is included in the Inquisit version. The primary measure of working memory capacity is the automated operation span score (Unsworth et al., 2005), calculated as the total number of letters recalled across all error-free trials. Full task details can be found in Unsworth et al. (2005).

Cognitive Flexibility

As a measure of cognitive flexibility, we used a shorter and modified version of the Wisconsin card sorting test, the modified card sort test (Nelson, 1976). The computerized version of the Inquisit database[4] was used. This test consists of two decks of 24 cards (so a total of 48) and four stimulus cards. Each card includes different colors and numbers of signs. The signs are: plus sign, star, triangle, or circle. There are one, two, three, or four signs on each card. Signs can be red, green, blue, or yellow. The participant is asked to match each new card appearing on the screen with a stimulus card. Correctly matched cards are arranged in three categories according to color, sign, and number. After the participant performs four consecutive correct matches in one category (e.g.,, the category "color"), the computer switches without

[2] https://www.millisecond.com/download/library/stroop/
[3] https://www.millisecond.com/download/library/ospan/
[4] https://www.millisecond.com/download/library/cardsort/

© 2021 Hogrefe Publishing

This document is copyrighted by the American Psychological Association or one of its allied publishers.
This article is intended solely for the personal use of the individual user and is not to be disseminated broadly.

warning the rule to another category. After each choice, the participant is provided feedback about whether the response was correct or incorrect, but is not provided information regarding the correct matching category. The scoring procedures of the modified card sorting test are the same as the original Wisconsin card sorting test (Caffarra et al., 2004; Nelson, 1976). Participants were instructed to respond as fast and accurately as possible. Perseverative errors, which reflect the number of trials with decision errors when the matching rule has changed, was selected as the main dependent variable as it is considered the closest approximation of cognitive flexibility (Miyake et al., 2000).

Procedure

Participants were recruited via flyers on a campus of a single university and via social networks groups linked to the university. After contacting the experimenter team who first screened for potential exclusion criteria, they received an email containing the details about the experiment. Each participant was required to take part to two testing sessions (lasting around 100 min each; see Figure 1). The order of the experimental conditions was counterbalanced across participants. The two sessions were separated by one week, to keep learning effects to a minimum, and took place at the same time of day, given chronotype can influence HRV (Van Eekelen et al., 2004) and cognitive performance (Folkard, 1990). Prior to the testing sessions, participants were instructed not to drink or eat anything but water during the 2 hr before the experiment, nor to do any strenuous exercise or drink alcohol in the 24 hr before the experiment (Laborde, Mosley, et al., 2017).

At the beginning of the experiment, participants were asked to complete an informed consent form, and at the beginning of each testing session they completed a questionnaire regarding variables potentially influencing HRV (Laborde, Mosley, et al., 2017), in order to control whether the information sent via email has been abided. The participants were then asked to turn off their smartphone. The full experiment was protocolled by the experimenter. The course of events in both conditions was identical, except for the familiarization to slow-paced breathing and the slow-paced breathing exercise in the slow-paced breathing condition, which were paralleled in the control condition with watching the neutral TV documentary. At the beginning of the slow-paced breathing condition, participants received a short introduction video on how to perform the technique correctly, and the correct execution was checked by the experimenter. All participants managed to perform correctly the slow-paced breathing technique.

The experiment then continued either with the slow-paced breathing exercise, or with watching the TV documentary. A HRV resting measure was taken before and after this step. The HRV resting measure lasted 5 minutes, based on the Task Force recommendations (Malik et al., 1996). The participants then performed the three executive cognitive tests – the color word Stroop test, the automated operation span, and the modified card sorting test – in the same body position as during the resting measurement. The order of the tests was randomized across participants. However, for each participant the order was kept the same across experimental sessions. We used a 15″ flat-screen monitor (Dell GmbH, Frankfurt, Germany; 1,280 × 960 pixels at 60 Hz) at a viewing distance of 60 cm for all tests. The HRV resting measure was realized in a sitting position with eyes closed, knees at 90°, hands on the thighs. The participants were asked to move as little as possible during the experiment. At the end of the second testing session, participants were debriefed and thanked. The full experimental procedure is depicted in Figure 1.

Data Analysis

For the HRV data, RMSSD was extracted from the Kubios output. Respiratory frequency (the number of respiratory cycles per minute) was obtained multiplying the EDR (ECG derived respiration) value obtained via the Kubios algorithm by 60 (Tarvainen et al., 2014). All participants were breathing at six cycles per minute during the slow-paced breathing technique. For inhibition, accuracy was operationalized via the number of incorrect answers retrieved for the squares, congruent stimuli, and incongruent stimuli. Regarding reaction times, only the reaction times of the correct answers was analyzed. Reaction times data were subjected to two filters. In the first filter, trials with response times lower than 200 ms and higher than 3,000 ms were excluded in order to control for extreme results (see Putman & Berling, 2011). The second filter then screened for reaction times higher or lower than two standard deviations from the mean. These were also removed (Dresler et al., 2009). Working memory capacity was operationalized as the total number of letters recalled across all error-free trials. Cognitive flexibility was operationalized as the total number of perseverative errors, corresponding to a card matching error when the previous rule had changed (Nelson, 1976).

Data were checked for outliers and normality. A total of 0.96% univariate outliers cases were found and winsorized (± 2.58; Tabachnick & Fidell, 2012). Multivariate outliers were checked using Mahalanobis distance, with none identified. The behavioral data related to the executive functions dependent variables were normally distributed. The physiological data (RMSSD, HR, respiratory frequency) were not normally distributed, thus a log-transformation (log 10) was applied to achieve normal distribution, and this

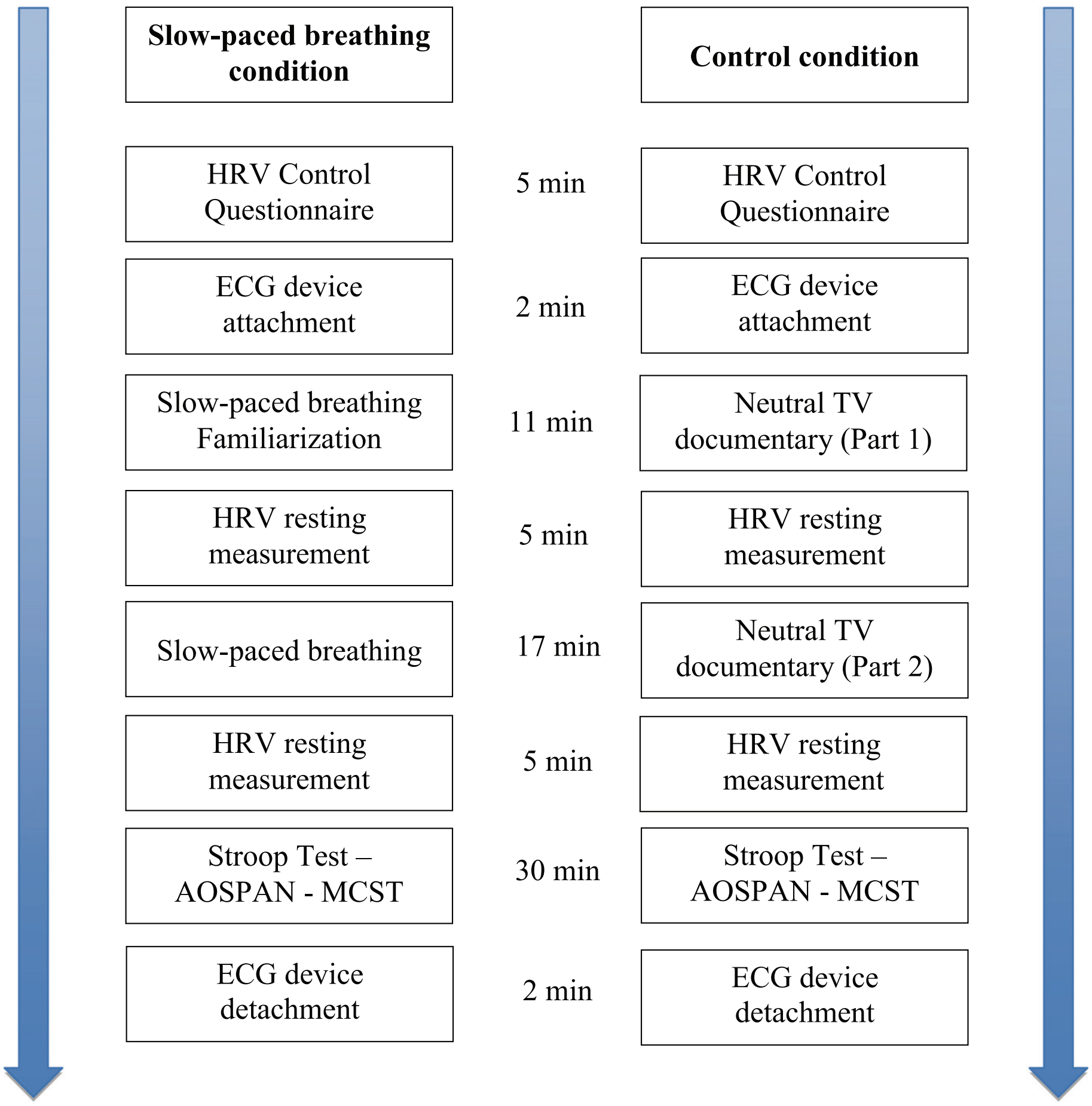

Figure 1. Illustration of the experimental protocol. HRV = Heart Rate Variability; AOSPAN = Automated Operation Span; MCST = Modified Card Sorting Test; ECG = Electrocardiography.

is consistent with previous HRV research (Laborde, Mosley, et al., 2017). For the physiological data, we ran analyses with the log-transformed values.

As a manipulation check (H1), we analyzed the difference between the resting HRV measurement before (PRE) the experimental manipulation (slow-paced breathing vs. TV documentary) and after the experimental manipulation (POST), for log10 HR, log10 RMSSD, and log10 respiratory frequency. For the experimental manipulation check, a repeated-measures MANOVA was conducted, with time as independent variable (PRE, DURING, POST) and with three dependent variables: log10 HR, log10 RMSSD, log10 respiratory frequency. Based on our hypotheses, we focus on the condition × time interaction. For log10 RMSSD, the analysis is first run without covariates and then with covariates included (session order, age, sex, BMI, waist-to-hip ratio), in order to see whether individual difference factors affect results.

Regarding our main hypothesis (H2), we conducted a repeated-measures MANOVA, with 2 conditions (slow-paced breathing vs. control) and four dependent variables: (1) accuracy (error rate to incongruent stimuli) and (2) reaction times (incongruent stimuli-congruent stimuli) for the Stroop interference (operationalization of inhibition), (3) the automated operation span (operationalization of working memory), and (4) the number of perseverative errors (operationalization of cognitive flexibility). Regarding Stroop interference accuracy, account errors made with congruent stimuli were not considered in the calculation given there were none. Significant interactions were followed-up using independent samples *t*-tests with Bonferroni corrected significance levels. Where a significant effect for slow-paced breathing was found on an executive function variable, potential mediation (H3) via log10 RMSSD (for both time points DURING and POST) was tested using PROCESS statistical software (Hayes, 2013). This custom dialog tests the total, direct, and indirect effect of an independent variable on a dependent variable through a proposed mediator and allows inferences regarding indirect effects using percentile bootstrap intervals.

This document is copyrighted by the American Psychological Association or one of its allied publishers.
This article is intended solely for the personal use of the individual user and is not to be disseminated broadly.

Table 1. Descriptive statistics for physiological variables

	Resting measurement before slow-paced breathing exercise/TV documentary (PRE)		Measurement during slow-paced breathing exercise/TV documentary (DURING)		Resting measurement after slow-paced breathing exercise/TV documentary (POST)	
	M	*SD*	*M*	*SD*	*M*	*SD*
Slow-paced breathing condition						
RMSSD (ms)	47.97	28.66	89.92	40.92	53.43	35.46
Heart rate (bpm)	71.00	10.93	66.27	9.77	69.57	11.02
Respiratory frequency (cpm)	10.48	1.97	6.56	0.26	10.89	1.80
RMSSD (log10)	1.61	0.25	1.91	0.20	1.64	0.27
Heart rate (log10)	1.85	0.07	1.82	0.07	1.84	0.07
Respiratory frequency (log10)	1.01	0.08	0.82	0.02	1.08	0.07
Control condition						
RMSSD (ms)	52.51	34.14	42.63	24.34	52.49	33.85
Heart rate (bpm)	70.93	11.63	70.70	11.92	71.29	11.46
Respiratory frequency (cpm)	13.73	2.58	16.57	2.72	13.68	2.32
RMSSD (log10)	1.63	0.07	1.56	0.07	1.63	0.07
Heart rate (log10)	1.84	0.08	1.84	0.08	1.85	0.07
Respiratory frequency (log10)	1.13	0.08	1.21	0.07	1.13	0.08

Note. RMSSD = Root mean square of the successive differences; ms = Milliseconds; bpm = Beats per minute; cpm = Cycles per minute.

This document is copyrighted by the American Psychological Association or one of its allied publishers.
This article is intended solely for the personal use of the individual user and is not to be disseminated broadly.

Results

Slow-Paced Breathing and Physiological Measures

Descriptive statistics for the physiological variables concerning the manipulation check can be seen in Table 1.

A repeated-measures MANOVA was conducted and showed an overall significant interaction effect for condition × time, Wilks' $\lambda = .046$, $F(6, 72) = 249.274$, $p < .001$, $\eta_p^2 = .95$. Univariate ANOVAs with Greenhouse-Geisser corrections were then ran for each physiological variable. A significant condition × time interaction effect was found for log10 HR, $F(1.181, 90.928) = 6.179$, $p < .001$, $\eta_p^2 = .07$. Nine follow-up post hoc *t*-tests (see Table 2A) were conducted with Bonferroni correction ($\alpha = .006$). The following significant differences were found: in the SPB condition, log10 HR was lower DURING in comparison to PRE ($p < .001$, $d = .43$) and POST ($p = .001$, $d = .41$), and lower in POST in comparison to PRE ($p = .001$, $d = .40$). Log10 HR DURING was also lower in the SPB condition in comparison to the CON condition ($p = .004$, $d = .34$).

A significant condition × time interaction effect was found for log10 RMSSD, $F(1.436, 110.550) = 95.689$, $p < .001$, $\eta_p^2 = .55$. Including the covariates (session order, age, sex, BMI, waist-to-hip ratio) did not change the pattern of results. Nine follow-up post hoc *t*-tests (see Table 2B) were conducted with Bonferroni correction ($\alpha = .006$). The following significant differences: In the CON condition, log10 RMSSD was lower DURING in comparison to PRE ($p = .001$, $d = 0.39$) and POST ($p = .001$, $d = .40$). In the SPB condition, log10 RMSSD was higher DURING in comparison to PRE ($p = .001$, $d = 1.06$) and POST ($p = .001$, $d = 0.88$). Only a tendency was found for POST being higher than PRE ($p = .020$, $d = .27$). Finally, log10 RMSSD was higher DURING in the SPB condition in comparison to the CON condition ($p < .001$, $d = 1.28$).

A significant condition × time interaction effect for log10 respiratory frequency was found $F(1.961, 150.959) = 599.516$, $p < .001$, $\eta_p^2 = .89$. Nine follow-up post hoc *t*-tests (see Table 2C) were conducted with Bonferroni correction ($\alpha = .006$). In the CON condition, log10 RF was higher DURING in comparison to PRE ($p < .001$, $d = 1.10$) and POST ($p < .001$, $d = 1.20$). In the SPB condition, log10 RF was lower DURING in comparison to PRE ($p < .001$, $d = 2.54$) and POST ($p < .001$, $d = 3.52$). It was also higher POST than PRE ($p < .001$, $d = 0.80$). When comparing the SPB and CON conditions, log10 RF was always lower in the SPB condition, PRE ($p < .001$, $d = 1.40$), DURING ($p < .001$, $d = 5.37$), and POST ($p < .001$, $d = 0.60$).

Slow-Paced Breathing and Behavioral Measures

Descriptive statistics for behavioral measures are reported in Table 3, and correlation matrices between all study variables are reported in Table 4.

For the MANOVA, a significant overall effect for condition was found, Wilks' $\lambda = .70$, $F(4, 74) = 4.69$, $p = .002$,

 © 2021 Hogrefe Publishing

This document is copyrighted by the American Psychological Association or one of its allied publishers.
This article is intended solely for the personal use of the individual user and is not to be disseminated broadly.

Table 2. Post hoc tests for manipulation check – physiological variables

	t	*p*	Cohen's *d*
(A) Heart rate (bpm)			
CON PRE vs. CON POST	0.993	.324	0.11
CON PRE vs. CON DURING	0.471	.639	0.05
CON DURING vs. CON POST	0.966	.337	0.11
SPB PRE vs. SPB POST	3.828	< .001*	0.43
SPB PRE vs. SPB DURING	3.605	.001*	0.41
SPB DURING vs. SPB POST	3.536	.001*	0.40
CON PRE vs. SPB PRE	0.212	.832	0.02
CON POST vs. SPB POST	1.694	.094	0.19
CON DURING vs. SPB DURING	2.986	.004*	0.34
(B) RMSSD (ms)			
CON PRE vs. CON POST	0.020	.984	0.01
CON PRE vs. CON DURING	3.405	.001*	0.39
CON DURING vs. CON POST	3.498	.001*	0.40
SPB PRE vs. SPB POST	2.366	.020	0.27
SPB PRE vs. SPB DURING	9.391	< .001*	1.06
SPB DURING vs. SPB POST	7.746	< .001*	0.88
CON PRE vs. SPB PRE	0.916	.363	0.10
CON POST vs. SPB POST	0.346	.730	0.04
CON DURING vs. SPB DURING	11.257	< .001*	1.28
(C) Respiratory frequency (cpm)			
CON PRE vs. CON POST	0.108	.914	0.01
CON PRE vs. CON DURING	9.693	< .001*	1.10
CON DURING vs. CON POST	10.574	< .001*	1.20
SPB PRE vs. SPB POST	7.031	< .001*	0.80
SPB PRE vs. SPB DURING	22.426	< .001*	2.54
SPB DURING vs. SPB POST	31.095	< .001*	3.52
CON PRE vs. SPB PRE	12.343	< .001*	1.40
CON POST vs. SPB POST	5.334	< .001*	0.60
CON DURING vs. SPB DURING	47.451	< .001*	5.37

Note. df = 77. RMSSD = Root mean square of the successive differences; ms = Milliseconds; bpm = Beats per minute; cpm = Cycles per minute; PRE = Resting measurement before slow-paced breathing exercise/TV documentary; DURING = Resting measurement during slow-paced breathing exercise/TV documentary; POST = Resting measurement after slow-paced breathing exercise/TV documentary. $*p < .006$ (applying Bonferroni correction).

$\eta_p^2 = .20$. Univariate ANOVAs with Greenhouse-Geisser corrections showed a significant effect for Stroop accuracy, $F(1, 77) = 9.21$, $p = .003$, $\eta_p^2 = .11$. No significant effect was found for Stroop response time, $F(1, 77) = 0.81$, $p = .372$, $\eta_p^2 = .01$. A significant effect for working memory capacity was also found, $F(1, 77) = 5.66$, $p = .020$, $\eta_p^2 = .07$. A significant effect for cognitive flexibility was also found, $F(1, 77) = 5.32$, $p = .024$, $\eta_p^2 = .07$.

Mediation Analysis

To test whether the effects of slow-paced breathing on executive functions was mediated by RMSSD either DURING or POST, the experimental condition, coded as slow-paced breathing (1) or control (2), was entered as the independent variable, the variables operationalizing executive functions (Stroop accuracy, automated operation span score, perseverative errors) were entered successively as dependent variables, and RMSSD was entered as mediator variable, first considering RMSSD DURING and then RMSSD POST. A 10,000 resampling rate was used for the mediation analysis. For RMSSD DURING, the results from the bootstrapped mediation analyses revealed no significant indirect effect for Stroop accuracy (95% CI [−.01, .01]), no significant indirect effect for automated operation span score (95% CI [−.04, .01]) and no significant indirect effect for perseverative errors (95% CI [−.11, .73]). For RMSSD POST, the results from the bootstrapped mediation analyses revealed no significant indirect effect for Stroop accuracy (95% CI [−.07, .05]), no significant indirect effect for automated operation span score (95% CI [−1.60, 2.09]) and no significant indirect effect for perseverative errors (95% CI [−.07, .05]).

Discussion

This experiment tested the effects of slow-paced breathing on immediate executive functioning (inhibition, working memory, cognitive flexibility). Based on current theoretical perspectives on slow-paced breathing (Gerritsen & Band, 2018; Lehrer & Gevirtz, 2014; Mather & Thayer, 2018; Noble & Hochman, 2019), we hypothesized that cardiac vagal activity operationalized via RMSSD would be higher, and HR and respiratory frequency would be lower, during and after the experimental manipulation with slow-paced breathing compared to control (H1). This hypothesis was partially supported. During the experimental manipulation, RMSSD increased in the SPB condition, and was significantly higher in comparison to the CON condition. However, after the experimental manipulation, RMSSD values decreased significantly, while showing a tendency to remain slightly higher than the resting measurement before the experimental manipulation. After the experimental manipulation, no difference between conditions was observed for RMSSD, while respiratory frequency and HR were significantly lower in the SPB condition compared to the CON condition. Based on the neurovisceral integration model (Smith et al., 2017; Thayer et al., 2009), we also hypothesized that cognitive functioning would be better in the experimental group compared to control (H2) and that RMSSD would mediate this difference in executive functioning (H3). These hypotheses were also partially supported. Participants did show better executive functioning after slow-paced breathing compared to control (for Stroop

Table 3. Descriptive statistics for executive functions

	Slow-paced breathing condition		Control condition	
	M	*SD*	*M*	*SD*
Stroop interference reaction time (ms)	143.85	68.02	138.04	72.84
Stroop interference accuracy (number of errors)	0.32	0.47	0.58	0.73
Automated operation span score	42.63	16.02	38.87	16.72
Perseverative errors (MCST)	0.40	0.65	0.68	0.95

Note. MCST = Modified Card Sorting Test.

Table 4. Correlation between main study variables

	Control condition					Slow-paced breathing condition				
	1	2	3	4	5	1	2	3	4	5
1. Stroop interference reaction time (ms)										
2. Stroop interference accuracy (number of errors)	.21					.10				
3. Automated operation span score	−.32**	−.53**				−.16	−.28*			
4. Perseverative errors (MCST)	.07	.25*	−.15			.12	.21	−.34**		
5. RMSSD (resting measurement before performing executive tasks)	−.11	−.30**	.27*	−.20		−.11	−.26*	.42**	−.22	

Note. MCST = Modified Card Sorting Test; RMSSD = Root Mean Square of the Successive Differences. *$p < .05$; **$p < .01$.

interference accuracy, working memory capacity, cognitive flexibility, but not Stroop interference response time), but this change in executive functioning was not mediated by RMSSD.

Regarding the physiological effects of the slow-paced breathing exercise, the effects on RMSSD were found to be the strongest during the experimental manipulation with a significant increase. After the experimental manipulation effects on RMSSD fade out: compared to the CON condition, in the SPB condition HR and respiratory frequency were lower, supporting our predictions, but contrary to what we hypothesized no significant difference was found for RMSSD. Based on several theoretical perspectives (Gerritsen & Band, 2018; Lehrer & Gevirtz, 2014; Mather & Thayer, 2018; Noble & Hochman, 2019), slow-paced breathing at 6 cpm is thought to increase vagal afferences and the longer exhalation phase in comparison to the inhalation phase is supposed to activate the parasympathetic nervous system (Strauss-Blasche et al., 2000; Van Diest et al., 2014). This is based on the coupling of heart beat and respiration with the respiratory sinus arrhythmia, where inhalation is linked to faster HR, and exhalation to slower HR (Angelone & Coulter, 1964; Yasuma & Hayano, 2004). Our findings illustrate that the increase in RMSSD, and hence in cardiac vagal activity, can be seen mostly during slow-paced breathing but not necessarily after, as suggested elsewhere (Bonomini et al., 2020; Kromenacker et al., 2018; You et al., 2021).

Two different patterns were observed for HR and respiratory frequency after the experimental manipulation. HR was found to be lower after the slow-paced breathing exercise than after watching the TV documentary, which would to some extent reflect the relaxation effect associated with slow-paced breathing (Gerritsen & Band, 2018; Lehrer & Gevirtz, 2014; Mather & Thayer, 2018; Noble & Hochman, 2019). A different pattern was observed for respiratory frequency given that it was found to be lower in the slow-paced breathing condition in comparison to the control condition *before* performing the slow-paced breathing technique. This is most likely due to the fact that in the slow-paced breathing condition, participants completed a familiarization exercise that decreased their breathing frequency (see Figure 1) prior to the first resting measurement. This reduced breathing pattern remained to some extent during the first resting measurement. The respiratory frequency then increased in the slow-paced breathing condition (when comparing the measurements before and after performing the slow-paced breathing exercise), that might be due to the participants becoming habituated to the reduced respiratory frequency while performing the technique, and returning faster to their normal respiratory frequency. However the fact that respiratory frequency was still lower in the slow-paced breathing condition than in the control condition (after the slow-paced breathing exercise and before starting the executive functioning tasks) supports the predicted effects of the slow-paced breathing technique at the respiratory level (Gerritsen & Band, 2018; Lehrer & Gevirtz, 2014; Mather & Thayer, 2018; Noble & Hochman, 2019).

Regarding our second hypothesis, executive functioning improved after the slow-paced breathing exercise in comparison to the control condition, for (1) inhibition with less

This document is copyrighted by the American Psychological Association or one of its allied publishers.
This article is intended solely for the personal use of the individual user and is not to be disseminated broadly.

errors for the incongruent stimuli (i.e., better Stroop interference accuracy), for (2) working memory capacity with a higher automated operation span score, and for (3) cognitive flexibility with less perseverative errors. No change was found on Stroop interference reaction time. Nevertheless, regarding Hypothesis 3, the results are not in line with predictions of the neurovisceral integration model (Smith et al., 2017; Thayer et al., 2009), given the improvement in executive functioning was not mediated by RMSSD, either considered during or after the experimental manipulation. However, it is important to note that in both the CON and the SPB condition, resting RMSSD before the executive tasks was correlated positively with performance on the Stroop interference accuracy and on the automated operation span score (while no associations were found with the Stroop interference reaction times and perseverative errors on the WCST), which would be partially in line with the neurovisceral integration model (Smith et al., 2017; Thayer et al., 2009). Taken together, these findings could suggest that other mechanisms might be contributing to the effects of slow-paced breathing on executive functioning. This might include improved functional connectivity in brain networks (Mather & Thayer, 2018; Tort et al., 2018). To explain, the slow oscillations in HR produced by slow-paced breathing are suggested to have the potential to strengthen brain network dynamics, especially in medial prefrontal regulatory regions that are particularly sensitive to physiological oscillations (Mather & Thayer, 2018), and which are responsible for cognitive control and emotion regulation (Thayer et al., 2012). In order to uncover the mechanisms at work, that might be involved in oxygenation, blood flow or electrophysiological signals in brain areas associated with executive functions, future research should investigate the effects of slow-paced breathing using brain imaging techniques such as EEG, *f*NIRS, *f*MRI, or MEG (Hoffmann et al., 2019; Hsu et al., 2020; Zhong et al., 2017).

Our experiment has some notable limitations that need to be considered when interpreting the main findings. First, our control condition involved spontaneous breathing while the participants were watching a neutral TV documentary. Therefore, we cannot rule out the possibility that the video may have introduced other psychological factors that differ from slow-paced breathing, given participants in the control group were not actively focusing on their breathing. Future studies should include a control condition that includes a similar focus on breathing at a different pace, perhaps 12 cycles per minute (Tsai et al., 2015) that would match the typical lower range spontaneous respiratory frequencies (Sherwood, 2006). Second, respiratory frequency was obtained via a dedicated algorithm from Kubios (Tarvainen et al., 2014). However, a more precise assessment of respiratory frequency such as a respiratory belt or a pneumotachograph (Egizio et al., 2011; Quintana & Heathers, 2014) and the assessment of other respiratory related variables (e.g., respiratory depth, gas exchanges) could prove helpful in explaining the effects of slow-paced breathing on executive functioning (Lorig, 2011; Ritz et al., 2002). Third, the initial physical activity level of the participants may have an influence on the results, and future research should consider assessing it using a standardized questionnaire such as the International Physical Activity Questionnaire (Craig et al., 2003). Fourth, some meaningful variations could be introduced to the slow-paced breathing technique practiced in this study. For example, having a break introduced between inhalation and exhalation, or between exhalation and inhalation, could have different physiological consequences (Reyes del Paso et al., 2015; Russell et al., 2017; Skow et al., 2015). Fifth, future studies should also consider the effects of long-term slow-paced breathing interventions (e.g., 4–5 weeks), given this is where most effects are found (Lehrer, 2018). Such long-term slow-paced breathing training plays an important role for maintaining autonomic homeostasis by stimulating the baroreflex functioning, which is then thought to provoke increasing resting cardiac vagal activity (Lehrer, 2003, 2013; Lehrer & Gevirtz, 2014; Vaschillo et al., 2006). Finally, future studies may also consider the specific influence of each respiratory phase (inspiratory phase, inspiratory-to-expiratory transition phase, expiratory phase, expiratory-to-inspiratory transition phase), on cognitive performance (Nakamura et al., 2018; Waselius et al., 2019).

Conclusion

In conclusion, this experiment tested the effects of slow-paced breathing on immediate executive functioning in a sample of young adults. The slow-paced breathing exercise triggered an increase of RMSSD during the experimental manipulation, which fade away after the experimental manipulation was over. Further, after the experimental manipulation a decrease in HR and respiratory frequency could still be observed. An increase in executive functioning was observed for inhibition, working memory, and cognitive flexibility, after the slow-paced breathing exercise compared to control. However, this increase in executive performance was not mediated by RMSSD (considered either during or after the experimental manipulation). Therefore, the influence of slow-paced breathing on executive functioning cannot be explained by an increase in cardiac vagal activity as predicted by the neurovisceral integration model (Smith et al., 2017; Thayer et al., 2009). Further research might want to test (using brain imaging) whether brain network dynamics are involved in the association between

This document is copyrighted by the American Psychological Association or one of its allied publishers.
This article is intended solely for the personal use of the individual user and is not to be disseminated broadly.

slow-paced breathing and executive functioning (Mather & Thayer, 2018). Finally, at the applied level, these findings may have implications for individuals looking for a quick and easy method to alter their executive functions, for example, to better execute cognitively demanding tasks in their jobs.

References

Alba, G., Vila, J., Rey, B., Montoya, P., & Munoz, M. A. (2019). The relationship between heart rate variability and electroencephalography functional connectivity variability is associated with cognitive flexibility. *Frontiers in Human Neuroscience, 13*, Article 64. https://doi.org/10.3389/fnhum.2019.00064

Albinet, C. T., Abou-Dest, A., Andre, N., & Audiffren, M. (2016). Executive functions improvement following a 5-month aquaerobics program in older adults: Role of cardiac vagal control in inhibition performance. *Biological Psychology, 115*, 69–77. https://doi.org/10.1016/j.biopsycho.2016.01.010

Albinet, C. T., Boucard, G., Bouquet, C. A., & Audiffren, M. (2010). Increased heart rate variability and executive performance after aerobic training in the elderly. *European Journal of Applied Physiology, 109*, 617–624. https://doi.org/10.1007/s00421-010-1393-y

Allen, B., & Friedman, B. H. (2012). Positive emotion reduces dyspnea during slow paced breathing. *Psychophysiology, 49*(5), 690–696. https://doi.org/10.1111/j.1469-8986.2011.01344.x

Angelone, A., & Coulter, N. A. Jr. (1964). Respiratory sinus arrhythemia: A frequency dependent phenomenon. *Journal of Applied Physiology, 19*(3), 479–482. https://doi.org/10.1152/jappl.1964.19.3.479

Baddeley, A. D., & Hitch, G. J. (1994). Developments in the concept of working memory. *Neuropsychology, 8*(4), 485–493. https://doi.org/10.1037/0894-4105.8.4.485

Berntson, G. G., Bigger, J. T., Eckberg, D. L., Grossman, P., Kaufmann, P. G., Malik, M., Nagaraja, H. N., Porges, S. W., Saul, J. P., Stone, P. H., & van der Molen, M. W. (1997). Heart rate variability: Origins, methods, and interpretive caveats. *Psychophysiology, 34*(6), 623–648. https://doi.org/10.1111/j.1469-8986.1997.tb02140.x

Biskamp, J., Bartos, M., & Sauer, J. F. (2017). Organization of prefrontal network activity by respiration-related oscillations. *Scientific Reports, 7*, Article 45508. https://doi.org/10.1038/srep45508

Bonomini, M. P., Calvo, M. V., Morcillo, A. D., Segovia, F., Vicente, J. M. F., & Fernandez-Jover, E. (2020). The effect of breath pacing on task switching and working memory. *International Journal of Neural Systems, 30*(6), Article 2050028. https://doi.org/10.1142/S0129065720500288

Bordoni, B., Purgol, S., Bizzarri, A., Modica, M., & Morabito, B. (2018). The influence of breathing on the central nervous system. *Cureus, 10*(6), Article e2724. https://doi.org/10.7759/cureus.2724

Brodal, P. (2016). *The central nervous system – Structure and function* (5th ed.). Oxford University Press.

Caffarra, P., Vezzadini, G., Dieci, F., Zonato, F., & Venneri, A. (2004). Modified card sorting test: Normative data. *Journal of Clinical and Experimental Neuropsychology, 26*(2), 246–250. https://doi.org/10.1076/jcen.26.2.246.28087

Colzato, L. S., Jongkees, B. J., de Wit, M., van der Molen, M. J. W., & Steenbergen, L. (2018). Variable heart rate and a flexible mind: Higher resting-state heart rate variability predicts better task-switching. *Cognitive, Affective, & Behavioral Neuroscience, 18*, 730–738. https://doi.org/10.3758/s13415-018-0600-x

Craig, C. L., Marshall, A. L., Sjostrom, M., Bauman, A. E., Booth, M. L., Ainsworth, B. E., Pratt, M., Ekelund, U., Yngve, A., Sallis, J. F., & Oja, P. (2003). International physical activity questionnaire: 12-country reliability and validity. *Medicine & Science in Sports & Exercise, 35*(8), 1381–1395. https://doi.org/10.1249/01.MSS.0000078924.61453.FB

Davidson, M. C., Amso, D., Anderson, L. C., & Diamond, A. (2006). Development of cognitive control and executive functions from 4 to 13 years: evidence from manipulations of memory, inhibition, and task switching. *Neuropsychologia, 44*(11), 2037–2078. https://doi.org/10.1016/j.neuropsychologia.2006.02.006

De Couck, M., Caers, R., Musch, L., Fliegauf, J., Giangreco, A., & Gidron, Y. (2019). How breathing can help you make better decisions: Two studies on the effects of breathing patterns on heart rate variability and decision-making in business cases. *International Journal of Psychophysiology, 139*, 1–9. https://doi.org/10.1016/j.ijpsycho.2019.02.011

Diamond, A. (2013). Executive functions. *Annual Review of Psychology, 64*, 135–168. https://doi.org/10.1146/annurev-psych-113011-143750

Diamond, A., & Ling, D. S. (2016). Conclusions about interventions, programs, and approaches for improving executive functions that appear justified and those that, despite much hype, do not. *Developmental Cognitive Neuroscience, 18*, 34–48. https://doi.org/10.1016/j.dcn.2015.11.005

Dresler, T., Meriau, K., Heekeren, H. R., & Van Der Meer, E. (2009). Emotional Stroop task: Effect of word arousal and subject anxiety on emotional interference. *Psychological Research, 73*(3), 364–371. https://doi.org/10.1007/s00426-008-0154-6

Egizio, V. B., Eddy, M., Robinson, M., & Jennings, J. R. (2011). Efficient and cost-effective estimation of the influence of respiratory variables on respiratory sinus arrhythmia. *Psychophysiology, 48*(4), 488–494. https://doi.org/10.1111/j.1469-8986.2010.01086.x

Fagevik Olsen, M., Lannefors, L., & Westerdahl, E. (2015). Positive expiratory pressure – Common clinical applications and physiological effects. *Respiratory Medicine, 109*(3), 297–307. https://doi.org/10.1016/j.rmed.2014.11.003

Faul, F., Erdfelder, E., Buchner, A., & Lang, A. G. (2009). Statistical power analyses using G*Power 3.1: Tests for correlation and regression analyses. *Behavior Research Methods, 41*, 1149–1160. https://doi.org/10.3758/BRM.41.4.1149

Folkard, S. (1990). Circadian performance rhythms: Some practical and theoretical implications. *Philosophical Transactions of the Royal Society B: Biological Sciences, 327*(1241), 543–553.

Gerritsen, R. J. S., & Band, G. P. H. (2018). Breath of Life: The respiratory vagal stimulation model of contemplative activity. *Frontiers in Human Neuroscience, 12*, Article 397. https://doi.org/10.3389/fnhum.2018.00397

Gothe, N., Pontifex, M. B., Hillman, C., & McAuley, E. (2013). The acute effects of yoga on executive function. *Journal of Physical Activity and Health, 10*(4), 488–495.

Grossman, P., Karemaker, J., & Wieling, W. (1991). Prediction of tonic parasympathetic cardiac control using respiratory sinus arrhythmia: The need for respiratory control. *Psychophysiology, 28*(2), 201–216. https://doi.org/10.1111/j.1469-8986.1991.tb00412.x

Grossman, P., & Kollai, M. (1993). Respiratory sinus arrhythmia, cardiac vagal tone, and respiration: Within- and between-individual relations. *Psychophysiology, 30*(5), 486–495.

Hansen, A. L., Johnsen, B. H., Sollers, J. J., Stenvik, K., & Thayer, J. F. (2004). Heart rate variability and its relation to prefrontal cognitive function: The effects of training and detraining. *European Journal of Applied Physiology, 93*, 263–272. https://doi.org/10.1007/s00421-004-1208-0

This document is copyrighted by the American Psychological Association or one of its allied publishers.
This article is intended solely for the personal use of the individual user and is not to be disseminated broadly.

This document is copyrighted by the American Psychological Association or one of its allied publishers.
This article is intended solely for the personal use of the individual user and is not to be disseminated broadly.

Hansen, A. L., Johnsen, B. H., & Thayer, J. F. (2003). Vagal influence on working memory and attention. *International Journal of Psychophysiology, 48*, 263–274. https://doi.org/10.1016/S0167-8760(03)00073-4

Hansen, A. L., Johnsen, B. H., & Thayer, J. F. (2009). Relationship between heart rate variability and cognitive function during threat of shock. *Anxiety, Stress, and Coping, 22*(1), 77–89. https://doi.org/10.1080/10615800802272251

Hayes, A. F. (2013). *Introduction to mediation, moderation, and conditional process analysis*. Guilford Press.

Heck, D. H., Kozma, R., & Kay, L. M. (2019). The rhythm of memory: How breathing shapes memory function. *Journal of Neurophysiology, 122*(2), 563–571. https://doi.org/10.1152/jn.00200.2019

Hill, L. K., Siebenbrock, A., Sollers, J. J., & Thayer, J. F. (2009). Are all measures created equal? Heart rate variability and respiration. *Biomedical Sciences Instrumentation, 45*, 71–76.

Hoffmann, S., Jendreizik, L., Ettinger, U., & Laborde, S. (2019). Keeping the pace: the effect of slow-paced breathing on error monitoring. *International Journal of Psychophysiology, 146*, 217–224. https://doi.org/10.1016/j.ijpsycho.2019.10.001

Hovland, A., Pallesen, S., Hammar, Å., Hansen, A. L., Thayer, J. F., Tarvainen, M. P., & Nordhus, I. H. (2012). The relationships among heart rate variability, executive functions, and clinical variables in patients with panic disorder. *International Journal of Psychophysiology, 86*, 269–275. https://doi.org/10.1016/j.ijpsycho.2012.10.004

Hsu, S. M., Tseng, C. H., Hsieh, C. H., & Hsieh, C. W. (2020). Slow-paced inspiration regularizes alpha phase dynamics in the human brain. *Journal of Neurophysiology, 123*(1), 289–299. https://doi.org/10.1152/jn.00624.2019

Johnsen, B. H., Thayer, J. F., Laberg, J. C., Wormnes, B., Raadal, M., Skaret, E., Kvale, G., & Berg, E. (2003). Attentional and physiological characteristics of patients with dental anxiety. *Journal of Anxiety Disorders, 17*(1), 75–87. https://doi.org/10.1016/S0887-6185(02)00178-0

Kane, M. J., & Engle, R. W. (2003). Working-memory capacity and the control of attention: The contributions of goal neglect, response competition, and task set to Stroop interference. *Journal of Experimental Psychology: General, 132*(1), 47–70. https://doi.org/10.1037/0096-3445.132.1.47

Kromenacker, B. W., Sanova, A. A., Marcus, F. I., Allen, J. J. B., & Lane, R. D. (2018). Vagal mediation of low-frequency heart rate variability during slow yogic breathing. *Psychosomatic Medicine, 80*(6), 581–587. https://doi.org/10.1097/psy.0000000000000603

Laborde, S., Allen, M. S., Gohring, N., & Dosseville, F. (2017). The effect of slow-paced breathing on stress management in adolescents with intellectual disability. *Journal of Intellectual Disability Research, 61*(6), 560–567. https://doi.org/10.1111/jir.12350

Laborde, S., Furley, P., & Schempp, C. (2015). The relationship between working memory, reinvestment, and heart rate variability. *Physiology & Behavior, 139*, 430–436. https://doi.org/10.1016/j.physbeh.2014.11.036

Laborde, S., Hosang, T., Mosley, E., & Dosseville, F. (2019). Influence of a 30-day slow paced breathing intervention compared to social media use on subjective sleep quality and cardiac vagal activity. *Journal of Clinical Medicine, 8*(2), 193–205. https://doi.org/10.3390/jcm8020193

Laborde, S., Lentes, T., Hosang, T. J., Borges, U., Mosley, E., & Dosseville, F. (2019). Influence of slow-paced breathing on inhibition after physical exertion. *Frontiers in Psychology, 10*, Article 1923. https://doi.org/10.3389/fpsyg.2019.01923

Laborde, S., Mosley, E., & Mertgen, A. (2018). A unifying conceptual framework of factors associated to cardiac vagal control. *Heliyon, 4*(12), Article e01002. https://doi.org/10.1016/j.heliyon.2018.e01002

Laborde, S., Mosley, E., & Thayer, J. F. (2017). Heart rate variability and cardiac vagal tone in psychophysiological research – Recommendations for experiment planning, data analysis, and data reporting. *Frontiers in Physiology, 8*, Article 213. https://doi.org/10.3389/fpsyg.2017.00213

Laborde, S., Mosley, E., & Ueberholz, L. (2018). Enhancing cardiac vagal activity: Factors of interest for sport psychology. *Progress in Brain Research, 240*, 71–92. https://doi.org/10.1016/bs.pbr.2018.09.002

Larsen, P. D., Tzeng, Y. C., Sin, P. Y., & Galletly, D. C. (2010). Respiratory sinus arrhythmia in conscious humans during spontaneous respiration. *Respiratory Physiology & Neurobiology, 174*(1–2), 111–118. https://doi.org/10.1016/j.resp.2010.04.021

Lehrer, P. M. (2003). Applied psychophysiology: Beyond the boundaries of biofeedback (mending a wall, a brief history of our field, and applications to control of the muscles and cardiorespiratory systems). *Applied Psychophysiology & Biofeedback, 28*(4), 291–304. https://doi.org/10.1023/a:1027330909265

Lehrer, P. M. (2013). How does heart rate variability biofeedback work? Resonance, the baroreflex, and other mechanisms. *Biofeedback, 41*(1), 26–31. https://doi.org/10.5298/1081-5937-41.1.02

Lehrer, P. M. (2018). Heart rate variability biofeedback and other psychophysiological procedures as important elements in psychotherapy. *International Journal of Psychophysiology, 131*, 89–95. https://doi.org/10.1016/j.ijpsycho.2017.09.012

Lehrer, P. M., & Gevirtz, R. (2014). Heart rate variability biofeedback: how and why does it work? *Frontiers in Psychology, 5*, Article 756. https://doi.org/10.3389/fpsyg.2014.00756

Lewis, G. F., Hourani, L., Tueller, S., Kizakevich, P., Bryant, S., Weimer, B., & Strange, L. (2015). Relaxation training assisted by heart rate variability biofeedback: Implication for a military predeployment stress inoculation protocol. *Psychophysiology, 52*(9), 1167–1174. https://doi.org/10.1111/psyp.12455

Lorig, T. (2011). The respiratory system. In J. T. Cacioppo, L. G. Tassinary, & G. G. Berntson (Eds.), *The handbook of psychophysiology* (pp. 231–244). Cambridge University Press.

Malik, M., Bigger, J. T., Camm, A. J., Kleiger, R. E., Malliani, A., Moss, A. J., & Schwartz, P. J. (1996). Heart rate variability. Standards of measurement, physiological interpretation, and clinical use. *European Heart Journal, 17*(3), 354–381. https://doi.org/10.1093/oxfordjournals.eurheartj.a014868

Maric, V., Ramanathan, D., & Mishra, J. (2020). Respiratory regulation and interactions with neuro-cognitive circuitry. *Neuroscience & Biobehavioral Reviews, 112*, 95–106. https://doi.org/10.1016/j.neubiorev.2020.02.001

Mather, M., & Thayer, J. F. (2018). How heart rate variability affects emotion regulation brain networks. *Current Opinion in Behavioral Sciences, 19*, 98–104. https://doi.org/10.1016/j.cobeha.2017.12.017

Mathewson, K. J., Jetha, M. K., Goldberg, J. O., & Schmidt, L. A. (2012). Autonomic regulation predicts performance on Wisconsin Card Sorting Test (WCST) in adults with schizophrenia. *Biological Psychology, 91*, 389–399. https://doi.org/10.1016/j.biopsycho.2012.09.002

Mayer, A. F., Karloh, M., Dos Santos, K., de Araujo, C. L. P., & Gulart, A. A. (2018). Effects of acute use of pursed-lips breathing during exercise in patients with COPD: A systematic review and meta-analysis. *Physiotherapy, 104*(1), 9–17. https://doi.org/10.1016/j.physio.2017.08.007

McDowd, J. M., Oseas-Kreger, D. M., & Filion, D. L. (1995). Inhibitory processes in cognition and aging. In F. N. Dempster & C. J. Brainerd (Eds.), *Interference and inhibition in cognition* (pp. 363–400). Academic Press.

Miller, E. K., & Cohen, J. D. (2001). An integrative theory of prefrontal cortex function. *Annual Review of Neuroscience, 24*(1), 167–202. https://doi.org/10.1146/annurev.neuro.24.1.167

© 2021 Hogrefe Publishing

This document is copyrighted by the American Psychological Association or one of its allied publishers.
This article is intended solely for the personal use of the individual user and is not to be disseminated broadly.

Millisecond Software. (2016). *Inquisit 5* [Computer software]. https://www.millisecond.com

Milner, B. (1982). Some cognitive effects of frontal-lobe lesions in man. *Philosophical Transactions of the Royal Society B: Biological Sciences, 298*(1089), 211–226. https://doi.org/10.1098/rstb.1982.0083

Miyake, A., & Friedman, N. P. (2012). The nature and organization of individual differences in executive functions: Four general conclusions. *Current Directions in Psychological Science, 21*(1), 8–14. https://doi.org/10.1177/0963721411429458

Miyake, A., Friedman, N. P., Emerson, M. J., Witzki, A. H., Howerter, A., & Wager, T. D. (2000). The unity and diversity of executive functions and their contributions to complex "Frontal Lobe" tasks: A latent variable analysis. *Cognitive Psychology, 41*(1), 49–100. https://doi.org/10.1006/cogp.1999.0734

Morandi, G. N., Lin, S. H., Lin, C. W., Yeh, T. L., Chu, C. L., Lee, I. H., Chi, M. H., Chen, K. C., Chen, P. S., & Yang, Y. K. (2019). Heart rate variability is associated with memory in females. *Applied Psychophysiology & Biofeedback, 44*(2), 117–122. https://doi.org/10.1007/s10484-018-9425-1

Mosley, E., Laborde, S., & Kavanagh, E. (2018). Coping related variables, cardiac vagal activity and working memory performance under pressure. *Acta Psychologica, 191*, 179–189. https://doi.org/10.1016/j.actpsy.2018.09.007

Nakamura, N. H., Fukunaga, M., & Oku, Y. (2018). Respiratory modulation of cognitive performance during the retrieval process. *PLoS One, 13*(9), Article e0204021. https://doi.org/10.1371/journal.pone.0204021

Nelson, H. E. (1976). A modified card sorting test sensitive to frontal lobe defects. *Cortex, 12*(4), 313–324.

Nguyen, L., Murphy, K., & Andrews, G. (2019). Immediate and long-term efficacy of executive functions cognitive training in older adults: A systematic review and meta-analysis. *Psychological Bulletin, 145*(7), 698–733. https://doi.org/10.1037/bul0000196

Noble, D. J., Goolsby, W. N., Garraway, S. M., Martin, K. K., & Hochman, S. (2017). Slow breathing can be operantly conditioned in the rat and may reduce sensitivity to experimental stressors. *Frontiers in Physiology, 8*, Article 854. https://doi.org/10.3389/fphys.2017.00854

Noble, D. J., & Hochman, S. (2019). Hypothesis: Pulmonary afferent activity patterns during slow, deep breathing contribute to the neural induction of physiological relaxation. *Frontiers in Physiology, 10*, Article 1176. https://doi.org/10.3389/fphys.2019.01176

Perl, O., Ravia, A., Rubinson, M., Eisen, A., Soroka, T., Mor, N., Secundo, L., & Sobel, N. (2019). Human non-olfactory cognition phase-locked with inhalation. *Nature Human Behaviour, 3*(5), 501–512. https://doi.org/10.1038/s41562-019-0556-z

Prinsloo, G. E., Rauch, H. G., Lambert, M. I., Muench, F., Noakes, T. D., & Derman, W. E. (2011). The effect of short duration heart rate variability (HRV) biofeedback on cognitive performance during laboratory induced cognitive stress. *Applied Cognitive Psychology, 25*, 792–801. https://doi.org/10.1002/acp.1750

Pu, J., Schmeichel, B. J., & Demaree, H. A. (2010). Cardiac vagal control predicts spontaneous regulation of negative emotional expression and subsequent cognitive performance. *Biological Psychology, 84*, 531–540. https://doi.org/10.1016/j.biopsycho.2009.07.006

Putman, P., & Berling, S. (2011). Cortisol acutely reduces selective attention for erotic words in healthy young men. *Psychoneuroendocrinology, 36*(9), 1407–1417. https://doi.org/10.1016/j.psyneuen.2011.03.015

Quintana, D. S., & Heathers, J. A. (2014). Considerations in the assessment of heart rate variability in biobehavioral research. *Frontiers in Physiology, 5*, Article 805. https://doi.org/10.3389/fpsyg.2014.00805

Reyes del Paso, G. A., Ladron, Munoz, de Guevara, C., & Montoro, C. I. (2015). Breath-holding during exhalation as a simple manipulation to reduce pain perception. *Pain Medicine, 16*(9), 1835–1841. https://doi.org/10.1111/pme.12764

Ritz, T., Dahme, B., Dubois, A. B., Folgering, H., Fritz, G. K., Harver, A., Kotses, H., Lehrer, P. M., Ring, C., Steptoe, A., & Van de Woestijne, K. P. (2002). Guidelines for mechanical lung function measurements in psychophysiology. *Psychophysiology, 39*(5), 546–567. https://doi.org/10.1017.S0048577202010715

Russell, M. E. B., Scott, A. B., Boggero, I. A., & Carlson, C. R. (2017). Inclusion of a rest period in diaphragmatic breathing increases high frequency heart rate variability: Implications for behavioral therapy. *Psychophysiology, 54*(3), 358–365. https://doi.org/10.1111/psyp.12791

Sebastiani, L., Di Gruttola, F., Incognito, O., Menardo, E., & Santarcangelo, E. L. (2019). The higher the basal vagal tone the better the motor imagery ability. *Archives Italiennes De Biologie, 157*(1), 3–14. https://doi.org/10.12871/00039829201911

Shannahoff-Khalsa, D. S., Boyle, M. R., & Buebel, M. E. (1991). The effects of unilateral forced nostril breathing on cognition. *International Journal of Neuroscience, 57*(3–4), 239–249. https://doi.org/10.3109/00207459109150697

Sherwood, L. (2006). *Fundamentals of physiology: A human perspective* (3rd ed.). Brooks/Cole.

Skow, R. J., Day, T. A., Fuller, J. E., Bruce, C. D., & Steinback, C. D. (2015). The ins and outs of breath holding: Simple demonstrations of complex respiratory physiology. *Advances in Physiology Education, 39*(3), 223–231. https://doi.org/10.1152/advan.00030.2015

Smith, R., Thayer, J. F., Khalsa, S. S., & Lane, R. D. (2017). The hierarchical basis of neurovisceral integration. *Neuroscience & Biobehavioral Reviews, 75*, 274–296. https://doi.org/10.1016/j.neubiorev.2017.02.003

Strauss-Blasche, G., Moser, M., Voica, M., McLeod, D. R., Klammer, N., & Marktl, W. (2000). Relative timing of inspiration and expiration affects respiratory sinus arrhythmia. *Clinical and Experimental Pharmacology and Physiology, 27*(8), 601–606.

Stroop, J. R. (1935). Studies of interference in serial verbal reactions. *Journal of Experimental Psychology, 18*(6), 643–662. https://doi.org/10.1037/h0054651

Stuss, D. T., Levine, B., Alexander, M. P., Hong, J., Palumbo, C., Hamer, L., Murphy, K. J., & Izukawa, D. (2000). Wisconsin Card Sorting Test performance in patients with focal frontal and posterior brain damage: Effects of lesion location and test structure on separable cognitive processes. *Neuropsychologia, 38*(4), 388–402.

Subramanya, P., & Telles, S. (2015). Performance in the Stroop task and simultaneously recorded heart rate variability before and after meditation, supine rest and no-intervention. *International Journal of Brain and Cognitive Sciences, 4*, 8–14. https://doi.org/10.5923/j.ijbcs.20150401.03

Szulczewski, M. T., & Rynkiewicz, A. (2018). The effects of breathing at a frequency of 0.1 Hz on affective state, the cardiovascular system, and adequacy of ventilation. *Psychophysiology, 55*(12), Article e13221. https://doi.org/10.1111/psyp.13221

Tabachnick, B., & Fidell, L. (2012). *Using multivariate statistics* (6th ed.). Pearson.

Takacs, Z. K., & Kassai, R. (2019). The efficacy of different interventions to foster children's executive function skills: A series of meta-analyses. *Psychological Bulletin, 145*(7), 653–697. https://doi.org/10.1037/bul0000195

Tarvainen, M. P., Niskanen, J. P., Lipponen, J. A., Ranta-Aho, P. O., & Karjalainen, P. A. (2014). Kubios HRV – Heart rate variability analysis software. *Computer Methods Programs Biomedical, 113*(1), 210–220. https://doi.org/10.1016/j.cmpb.2013.07.024

 © 2021 Hogrefe Publishing

Thayer, J. F., Ahs, F., Fredrikson, M., Sollers, J. J., & Wager, T. D. (2012). A meta-analysis of heart rate variability and neuroimaging studies: Implications for heart rate variability as a marker of stress and health. *Neuroscience & Biobehavioral Reviews, 36*, 747–756. https://doi.org/10.1016/j.neubiorev.2011.11.009

Thayer, J. F., Hansen, A. L., Saus-Rose, E., & Johnsen, B. H. (2009). Heart rate variability, prefrontal neural function, and cognitive performance: the neurovisceral integration perspective on self-regulation, adaptation, and health. *Annals of Behavioral Medicine, 37*, 141–153. https://doi.org/10.1007/s12160-009-9101-z

Thayer, J. F., & Lane, R. D. (2009). Claude Bernard and the heart-brain connection: Further elaboration of a model of neurovisceral integration. *Neuroscience & Biobehavioral Reviews, 33*, 81–88. https://doi.org/10.1016/j.neubiorev.2008.08.004

Thayer, J. F., Loerbroks, A., & Sternberg, E. M. (2011). Inflammation and cardiorespiratory control: The role of the vagus nerve. *Respiratory Physiology & Neurobiology, 178*, 387–394. https://doi.org/10.1016/j.resp.2011.05.016

Tort, A. B. L., Brankack, J., & Draguhn, A. (2018). Respiration-entrained brain rhythms are global but often overlooked. *Trends in Neuroscience, 41*(4), 186–197. https://doi.org/10.1016/j.tins.2018.01.007

Tsai, H. J., Kuo, T. B., Lee, G. S., & Yang, C. C. (2015). Efficacy of paced breathing for insomnia: Enhances vagal activity and improves sleep quality. *Psychophysiology, 52*(3), 388–396. https://doi.org/10.1111/psyp.12333

Turner, M. L., & Engle, R. W. (1989). Is working memory capacity task dependent? *Journal of Memory & Language, 28*, 127–154.

Unsworth, N., Heitz, R. P., Schrock, J. C., & Engle, R. W. (2005). An automated version of the operation span task. *Behavior Research Methods, 37*, 498–505. https://doi.org/10.3758/BF03192720

Van Diest, I., Verstappen, K., Aubert, A. E., Widjaja, D., Vansteenwegen, D., & Vlemincx, E. (2014). Inhalation/exhalation ratio modulates the effect of slow breathing on heart rate variability and relaxation. *Applied Psychophysiology & Biofeedback, 39*(3–4), 171–180. https://doi.org/10.1007/s10484-014-9253-x

Van Eekelen, A. P., Houtveen, J. H., & Kerkhof, G. A. (2004). Circadian variation in cardiac autonomic activity: Reactivity measurements to different types of stressors. *Chronobiology International, 21*(1), 107–129. https://doi.org/10.1081/cbi-120027983

Vaschillo, E. G., Vaschillo, B., & Lehrer, P. M. (2006). Characteristics of resonance in heart rate variability stimulated by biofeedback. *Applied Psychophysiology & Biofeedback, 31*(2), 129–142. https://doi.org/10.1007/s10484-006-9009-3

Waselius, T., Wikgren, J., Penttonen, M., & Nokia, M. S. (2019). Breathe out and learn: Expiration-contingent stimulus presentation facilitates associative learning in trace eyeblink conditioning. *Psychophysiology, 56*(9), Article e13387. https://doi.org/10.1111/psyp.13387

Wells, R., Outhred, T., Heathers, J. A., Quintana, D. S., & Kemp, A. H. (2012). Matter over mind: A randomised-controlled trial of single-session biofeedback training on performance anxiety and heart rate variability in musicians. *PLoS One, 7*, Article e46597. https://doi.org/10.1371/journal.pone.0046597

West, J. B. (2015). *Essays on the history of respiratory physiology*. Springer.

West, J. B., & Luks, A. M. (2016). *West's respiratory physiology – The essentials*. Wolters Kluwer.

Yadav, G., & Mutha, P. K. (2016). Deep breathing practice facilitates retention of newly learned motor skills. *Scientific Reports, 6*, Article 37069. https://doi.org/10.1038/srep37069

Yasuma, F., & Hayano, J. (2004). Respiratory sinus arrhythmia: Why does the heartbeat synchronize with respiratory rhythm? *Chest, 125*, 683–690. https://doi.org/10.1378/chest.125.2.683

You, M., Laborde, S., Salvotti, C., Zammit, N., Mosley, E., & Dosseville, F. (2021). Influence of a single slow-paced breathing session on cardiac vagal activity in athletes. *International Journal of Mental Health and Addiction*. Advance online publication. https://doi.org/10.1007/s11469-020-00467-x

Zaccaro, A., Piarulli, A., Laurino, M., Garbella, E., Menicucci, D., Neri, B., & Gemignani, A. (2018). How breath-control can change your life: A systematic review on psycho-physiological correlates of slow breathing. *Frontiers in Human Neuroscience, 12*, Article r353. https://doi.org/10.3389/fnhum.2018.00353

Zelano, C., Jiang, H., Zhou, G., Arora, N., Schuele, S., Rosenow, J., & Gottfried, J. A. (2016). Nasal respiration entrains human limbic oscillations and modulates cognitive function. *Journal of Neuroscience, 36*(49), 12448–12467. https://doi.org/10.1523/jneurosci.2586-16.2016

Zhong, W., Ciatipis, M., Wolfenstetter, T., Jessberger, J., Muller, C., Ponsel, S., Yanovsky, Y., Brankačk, J., Tort, A. B. L., & Draguhn, A. (2017). Selective entrainment of gamma subbands by different slow network oscillations. *Proceedings of the National Academy of Sciences of the United States of America, 114*(17), 4519–4524. https://doi.org/10.1073/pnas.1617249114

History
Received September 4, 2020
Revision received January 13, 2021
Accepted January 15, 2021
Published online March 18, 2021

Sylvain Laborde
Department of Performance Psychology
Institute of Psychology
German Sport University
Am Sportpark Müngersdorf, 6
50937 Cologne
Germany
s.laborde@dshs-koeln.de

This document is copyrighted by the American Psychological Association or one of its allied publishers.
This article is intended solely for the personal use of the individual user and is not to be disseminated broadly.

© 2021 Hogrefe Publishing
