# Supplementary material for: Use of a Smartphone-Based App in an Online Behavioral Neuroscience Lab Course
Source: J Undergrad Neurosci Educ. 2026 Jul 1;24(2):34–43. doi: 10.59390/001c.163102 (PMC13625900; doi:10.59390/001c.163102)
Supplement: Supplementary file 3 [file junejournal_2026_24_2_163102_348909.pdf]

ELSEVIER

Journal of Affective Disorders 61 (2000) 201–216

JOURNAL OF AFFECTIVE DISORDERS

www.elsevier.com/locate/jad

A model of neurovisceral integration in emotion regulation and dysregulation

Julian F. Thayer[a,*], Richard D. Lane[b]

[a]*National Institute of Aging/Gerontology Research Center, 5600 Nathan Shock Drive, Baltimore, MD 21224, USA*
[b]*Department of Psychiatry, University of Arizona, Tuscon, AZ 85724-5002, USA*

Abstract

In the present paper we present the outlines of a model that integrates autonomic, attentional, and affective systems into a functional and structural network that may help to guide us in our understanding of emotion regulation and dysregulation. We will emphasize the relationship between attentional regulation and affective processes and propose a group of underlying physiological systems that serve to integrate these functions in the service of self-regulation and adaptability of the organism. We will attempt to place this network in the context of dynamical systems models which involve feedback and feedforward circuits with special attention to negative feedback mechanisms, inhibitory processes, and their role in response selection. From a systems perspective, inhibitory processes can be viewed as negative feedback circuits that allow for the interruption of ongoing behavior and the re-deployment of resources to other tasks. When these negative feedback mechanisms are compromised, positive feedback loops may develop as a result (of dis-inhibition). From this perspective, the relative sympathetic activation seen in anxiety disorders may represent *dis-inhibition* due to faulty inhibitory mechanisms. © 2000 Elsevier Science B.V. All rights reserved.

Keywords: Neurovisceral integration; Emotion regulation and dysregulation

1. Introduction

“*The secret of success lies not so much in the abundance of thought as in our ability to follow one well chosen subject to the exclusion of others* (p. 21) ... *One of the immediate results we obtain through the practice of right concentration is that our mind grows strong and flexible. It adapts itself with great ease to whatever may be placed before it, even something that is out of its accustomed line*.” (p. 25) Swami Paramananda (1974)

“*Everyone knows what attention is. It is the taking possession of the mind, in clear and vivid form, of one out of what seem several simultaneously possible objects or trains of thought. Focalization, concentration of consciousness are of its essence. It implies withdrawal from some things in order to deal effectively with others, and*

*Corresponding author. Tel.: +1-410-558-8612; fax: +1-410-558-8108.

E-mail address: thayer@lpc.grc.nia.nih.gov (J.F. Thayer).

0165-0327/00/$ – see front matter © 2000 Elsevier Science B.V. All rights reserved.
PII: S0165-0327(00)00338-4

is a condition which has a real opposite in the confused, dazed, scatter-brain state.'' (James, 1997)

The idea that inhibitory processes are important for adaptability and success of both the individual and the species is not new. The above quotations from one of the first Eastern yogic teachers to come to the United States and from one of the pioneers of American psychology were written in the early part of the twentieth century. The first represents a distillation of thousands of years of yoga psychology, the second the prescient writings of one of the most influential thinkers of the twentieth century. More recently, McGeer et al. (1978) note:

''*We can think that inhibition is a sculpturing process. The inhibition, as it were, chisels away at the diffuse and rather amorphous mass of excitatory action and gives a more specific form to neuronal performance at every stage of synaptic relay. This suppressing action of inhibition can be recognized very clearly at higher levels of the brain* . . . '' (p. 133).

A comprehensive model of emotions and disorders of affect must attempt to account for the complex mix of cognitive, affective, behavioral, and physiological concomitants of normal and pathological affective states and dispositions. In the present paper we present the outlines of a model that integrates some of these components into a functional and structural network that may help to guide us in our understanding of emotion regulation and dysregulation. Functionally, this network includes attentional regulation, classical conditioning, affective information processing, and behavioral and physiological flexibility. Structurally, this network includes central nervous system structures, particularly the cingulate cortex, and peripheral endorgans, particularly the cardiovascular system. We will briefly review some of the work that we have done on the functional aspects of emotion regulation and dysregulation tying these functions to specific structures. We will emphasize the relationship between attentional regulation and affective processes and propose a group of underlying physiological systems that serve to integrate these functions in the service of self-regulation and adaptability of the organism. We will attempt to place this network in the context of dynamical systems models which involve feedback and feedforward circuits with special attention to negative feedback mechanisms, inhibitory processes, and their role in response selection. Importantly, we will show that the arousal associated with anxiety represents a dis-inhibition of positive feedback circuits that are normally under tonic inhibitory control.

2. Functional and dysfunctional aspects of emotion

Emotions represent a distillation of an individual's perception of personally relevant interplay with the environment including not only challenges and threats but also their ability to respond to or cope with them (Frijda, 1988). As such, emotions serve as an integrative index of an individual's adjustment to the constantly changing environmental demands they face. Emotions have been characterized as an organismic response to an environmental event that allows for the rapid mobilization of multiple subsystems for action (Levenson, 1988). In this context, emotions are the moment-to-moment output of a continuous sequence of behavior. These behavioral sequences are organized around biologically important functions and these lawful sequences of behavior have been termed 'behavioral systems' (Timberlake, 1994). Put another way, emotions are self-regulatory responses that allow the efficient coordination of the organism for goal-directed behavior. For example, when faced with a potentially dangerous situation, a Defensive Behavior System might be activated. The first stage of the behavioral sequence could involve the experience of anxiety, a shift toward relative sympathetic autonomic activity including increased heart rate, and a selective search for signs of danger. If a source of danger is identified the next stage in the behavioral sequence might involve fear and the mobilization of the organism for fight or flight. As Frijda (1988) noted, specific emotions imply specific eliciting stimuli, specific action tendencies including selective attention to relevant stimuli, and specific reinforcers. When this system works properly it allows for the flexible adaptation of the organism to changing environmental demands. In another sense,

an emotional response represents a selection of an appropriate response and the inhibition of other less appropriate responses from a more or less broad behavioral repertoire.

These behavioral systems recruit a whole host of organism resources in the service of goal-directed behavior and adaptability (cf. Damasio, 1998). From a dynamical systems perspective an organism is a complex set of reverberating circuits or sub-systems working together in a coordinated fashion (a set of loosely coupled bio-oscillators). The individual elements of the organism can be thought of as a high dimensional system with a large number of degrees of freedom. However, in the service of goal-directed behavior and in the context of a behavioral system, these elements organize into coordinated assemblages that can be described by a smaller number of control parameters. This is not unlike the factors of factor analysis, which reveal the latent structure among a set of questionnaire items thereby reducing or mapping the high dimensional item space into a lower dimensional factor space. For example, the coordination of breathing, the direction of blood flow to various muscles and organs, the release of appropriate hormones such as cortisol, the detailed control of specific muscle fibers to aid in grasping or running, the control of the biochemical processes that support these processes, and the higher level information processing that accompanies the appraisal of threat and challenge represent just some of the responses at various levels of system organization, each with its own constraints and options (degrees of freedom) that must be coordinated in a fraction of a second (see Schwartz, 1986, for a detailed chart of various functions associated with an emotional response). This complex system, as it moves through time on numerous time-scales, requires various feedback and feedforward circuits for its efficient functioning. Moreover, the emotional response 'emerges' from the interaction of the various sub-systems with the environmental demands and this response is not orchestrated from a central command center. Thus, it is a distributed system.

These behavioral systems or preferred configurations and trajectories represent modes of relative stability in the constant flow of organism–environment interactions. As such, from a dynamical systems perspective emotions may represent attractors (preferred configurations) in the state-space (behavioral repertoire) of the organism. One consequence of this idea is that the high dimensional system may be guided through the emotional state-space by a smaller number of dimensions of change or control parameters. One of us has recently presented evidence to this effect (Johnsen et al., 1995; Nyklicek et al., 1997; Thayer and Friedman, 1997). Thus, discrete emotions may be viewed as attractors in the state-space defined by a small set of control parameters. A vast amount of research points to the two dimensions of valence and arousal as being the most important in this respect (Osgood et al., 1957; Johnsen et al., 1995; Nyklicek et al., 1997; Ohman et al., 1993; Russell, 1980). These dimensions have been viewed as representing the motivational systems of approach and avoidance (valence), and together with an index of the amount of vigor with which these drives are pursued (arousal) have been suggested as the underpinnings of goal-directed behavior in a wide range of organisms (cf. Schneirla, 1959).

Further, in the dynamical systems perspective, the basin of attraction for a given emotional state defines the set of parameter values that will lead to one emotion as opposed to another. That is, the unique combination of internal and external conditions (parameter values) that lead to the emergence of one behavioral system or sequence of emotions (attractor) over another is termed the basin of attraction. Within the context of the physiological constraints and the learning history of a given organism, the set of parameter values or environmental conditions that lead to one emotion versus another can vary greatly. The map of these parameter values and related attractors defines the behavioral repertoire, emotional topography or state-space of each individual.

We have asserted (Friedman and Thayer, 1998; Thayer and Friedman, 1997) that disorders of affect, including anxiety disorders, may be viewed as a kind of distorted emotional state space in which an individual is unable to shift into an attractor or emotion that is appropriate for a given set of environmental demands. As such the individual is 'stuck' in an attractor or behavioral pattern that is not responsive to the demands placed upon it by the environment (see also Stormark et al., 1998). This is manifested in inflexibility at various levels of system

organization. Put another way, the individual is unable to select the appropriate response or, more often the case, unable to inhibit the inappropriate response. Thus, the response selection mechanism is somehow corrupted.

We have investigated the ability to respond to or ‘read’ the emotional landscape and generate an appropriate response in the context of self-monitoring (Miller and Thayer, 1988, 1989) and levels of emotional awareness (Lane and Schwartz, 1987). This ‘reading’ of the emotional environment requires the selection of certain information and the disregarding of other information from a complex input that includes internal as well as external cues. When this ability to effectively process affective information is compromised for whatever reason, the individual is unable to maneuver efficiently in its environment. Inefficiency in affective information processing leads to affective dysregulation. When this inefficiency becomes severe, various forms of pathology are said to exist such as alexithmia, depression, panic disorder, generalized anxiety disorder, hostile personality, hypertension, and coronary heart disease to name a few.

A related aspect of this self-regulation involves selective attention. The ability to sustain and shift attention is an important component of organism self-regulation and adaptability. The capacity to select meaningful information and disregard irrelevant information from the external and internal environments is critical for the survival of the organism. Information that is particularly meaningful to a given individual will attract that person’s attention and resources.

What makes information meaningful to an individual is its impact on the well being of the organism. Does the information represent something aversive to be avoided or something appetitive to be approached? Thus, the selective attention to information is often of an affective or motivational character (Stormark et al., 1995; Stormark and Hugdahl, 1996). In this context, one can posit that attentional regulation and affective regulation are functionally an integrated system in the service of organism self-regulation and adaptability (see, for example, Heilman, 1997).

A number of researchers (e.g., Porges, 1991, 1992; Richards and Casey, 1992; Thayer et al., 2000) have suggested that autonomic nervous system regulation as manifested in cardiac variability may be related to both attentional regulation and affect. Cardiac vagal tone as reflected in heart rate variability (HRV) in particular has been shown to be related to attentional control and to emotional regulation (Nyklicek et al., 1997; Porges, 1991, 1992; Thayer et al., 2000). These researchers propose that measures of cardiac vagal tone index the efficiency of central–peripheral neural feedback mechanisms. Thus, these measures may serve to quantify the ability to self-regulate, through the organization of physiological resources and appropriate response selection, in the service of goal-directed behavior. Specifically, high vagal tone is associated with the ability to self-regulate and thus to have greater behavioral flexibility and adaptability in a changing environment. Low vagal tone, on the other hand, is associated with poor self-regulation and a lack of behavioral flexibility (Porges, 1992, p. 208). Thus, HRV appears to index vital aspects of self-regulation due to its ability to reflect neural feedback mechanisms of central nervous system (CNS)–autonomic nervous system (ANS) integration (Friedman and Thayer, 1998).

We have presented a functional framework that integrates affective regulation, attentional regulation, and heart rate variability. This functional system serves to describe the behavioral processes associated with goal-directed behavior and adaptability. We next describe the structural system that serves to instantiate this functional system in the physiological ‘wet-ware’ of an organism.

3. The central autonomic network and the anterior executive region

A number of researchers have identified functional units within the CNS that appear to support goal-directed behavior and adaptability. One such functional unit is the central autonomic network (CAN; Benarroch, 1993, 1997). Functionally, this network is an integrated component of an internal regulation system through which the brain controls visceromotor, neuroendocrine, and behavioral responses that are critical for goal-directed behavior and adaptability (Benarroch, 1993). Structurally, the CAN includes the anterior cingulate, insular, and ventromedial prefrontal cortices, the central nucleus of the amygdala, the paraventricular and related nuclei

of the hypothalamus, the periaquaductal gray matter, the parabrachial nucleus, the nucleus of the solitary tract (NTS), the nucleus ambiguus, the ventrolateral medulla, the ventromedial medulla, and the medullary tegmental field. The primary output of the CAN is mediated through the preganglionic sympathetic and parasympathetic neurons. Importantly, these neurons innervate the heart via the stellate ganglia and the vagus nerve. The interplay of these inputs to the sino-atrial node of the heart is the source of the complex variability that characterizes the heart rate time series (Saul, 1990). Thus, the output of the CAN is directly linked to heart rate variability (HRV). In addition, sensory information from the peripheral end organs such as the heart are fed back to the CAN, one important example of which is the baroreceptor reflex. As such, HRV is an index of central–peripheral neural feedback and CNS–ANS integration.

Moreover, the CAN has many features of a nonlinear dynamical system. First, the components of the CAN are reciprocally interconnected. This allows for continuous positive and negative feedback interactions and integration of autonomic responses. Second, the CAN is comprised of a number of parallel, distributed pathways, which allows for multiple avenues to a given response. For a simple example, a given heart rate change of 68 to 80 beats per minute can be achieved by various combinations of sympathetic and parasympathetic input to the sino-atrial node including increased sympathetic activity, decreased parasympathetic activity or any combination of the two, as well as via other pathways such as circulating hormones. Moreover, within the CAN direct and indirect pathways can modulate the output to the preganglionic sympathetic and parasympathetic neurons. Third, the activity of the CAN is state dependent and thus sensitive to initial conditions (see Glass and Mackey, 1988).

The CAN receives and integrates visceral, humoral, and environmental information and coordinates autonomic, endocrine, and behavioral responses to environmental challenges. Importantly, the CAN is under tonic inhibitory control. This is achieved by γ-aminobutyric acid (GABA) interneurons within the NTS. GABA is the main inhibitory neurotransmitter within the CNS. Disruption of this inhibitory pathway may lead to such things as hypertension and sinus tachycardia, and represents a dis-inhibition of sympathoexcitatory circuits within the CAN (Benarroch, 1993, 1997; Masterman and Cummings, 1997; Spyer, 1989).

Other functional units within the CNS subserving executive, social, affective, attentional, and motivated behavior in humans and animals have also been identified (Damasio, 1998; Devinsky et al., 1995; Masterman and Cummings, 1997; Posner and Petersen, 1990; Spyer, 1989). One such functional unit has been termed the anterior executive region (AER; Devinsky et al., 1995). Functionally, the AER and its projections ''assesses the motivational content of internal and external stimuli and regulates context-dependent behaviors.'' (Devinsky et al., 1995, p. 279). The AER and its projections has been termed the 'rostral limbic system' and structurally includes the anterior, insular, and orbitofrontal cortices, the amygdala, the periaquaductal gray, the ventral striatum, and autonomic brainstem motor nuclei. Another such functional unit has been identified by Damasio (1998) as the neural substrate for emotions. Table 1 lists the structures and functions associated with the CAN, the rostral limbic system, and the 'emotion circuit'. The structural overlap is quite substantial.

We propose that the CAN, the AER and its projections, the 'emotion circuit' (Damasio, 1998), and related systems (Masterman and Cummings, 1997; Spyer, 1989) are one and the same functional network identified by different researchers from differing orientations. This network of CNS structures is associated with the processes of response organization and selection, and serves to modulate psychophysiological resources in attention and emotion (Friedman and Thayer, 1998; Thayer and Friedman, 1997).

In the following we will briefly review several experimental investigations that we have conducted to help elucidate the function and structure of this network. Most of this work has been published elsewhere in detail and the interested reader is referred to those publications for an in-depth discussion of the experimental procedures.

4. HRV in emotion and psychopathology

Autonomically mediated cardiovascular variability is critical as an index of neurovisceral integration

Table 1
Structures and functions associated with the CAN, the rostral limbic system, and the 'emotion circuit'[a]

Circuit, source and function	IC	AC	PF	Amy	PG	Other areas
CAN (Benarroch, 1997)						
Cardiovascular control	X	X	X	X	X	NTS, NA
AER (Devinsky et al., 1995)						
Motivated and context-dependent behavior	X	X	X	X	X	Ventral striatum
Damasio (1998)						
Affect	X	X	X	X	X	BG

[a] Key: insular cortex (IC), anterior cingulate (AC), prefrontal cortex (PF), amygdala (Amy), periaquaductal gray (PG), nucleus of the solitary tract (NTS), nucleus ambiguus (NA), basal ganglia (BG).

and organism self-regulatory ability. The interplay of sympathetic and parasympathetic (vagal) outputs of the CAN at the sino-atrial node produces the complex beat-to-beat variability that is characteristic of a healthy, adaptive organism. Vagal influences dominant cardiovascular control and thus the cardiovascular system is under tonic inhibitory control via the vagus nerve (Levy, 1990). There are several lines of research that point to the importance of HRV in attention, emotion and affective disorders. This literature has recently been reviewed by Friedman and Thayer (1998). They concluded that a relative reduction in vagally mediated HRV is consistent with the cardiac symptoms of panic anxiety as well as with the psychological symptoms of poor attentional control, ineffective emotional regulation, and behavioral inflexibility. Recent studies on depression (Thayer et al., 2000) and generalized anxiety disorder (GAD; Thayer et al., 1996) have found similar reductions in HRV. This reduction of vagally mediated cardiovascular control serves to dis-inhibit sympathoexcitatory influences. Due to differences in the temporal kinetics of the autonomic neuroeffectors, sympathetic influences on cardiac control are relatively slow (order of magnitude seconds) compared to vagal influences (order of magnitude milliseconds; see Saul, 1990). Thus, when the fast vagal modulation of cardiac function is decreased, the organism is less able to track the rapid changes in environmental demands and less able to organize an appropriate response. In the remainder of this section, we will use our work on generalized anxiety disorder to illustrate some of the implications of this disruption of the inhibitory pathway for psychopathology. Similar models of vagal inhibition have recently been put forward to describe the relationship between psychological factors and physiological health. Brosschot and Thayer (1998) have related vagal inhibition to hostility and the risk for cardiovascular disease, and Sroka et al. (1997) have suggested vagal inhibition as the link between psychological factors and myocardial ischemia. The interested reader is referred to some of our other work on non-pathological emotion for the role of vagally mediated cardiac activity in various emotional states (Nyklicek et al., 1997), and emotional regulation (Sollers et al., 1997).

Thayer et al. (1996) examined the autonomic characteristics of GAD and its cardinal feature, worry. Autoregressive spectral analysis of heart period variability (HPV) was used to investigate the effects of a 10 min relaxation period and a 10 min worry period in persons with GAD and non-anxious controls. The results provided evidence for two main effects (see Fig. 1). One, persons with GAD had lower vagally-mediated HPV compared to non-anxious controls across all experimental conditions including baseline. Importantly, a second main effect appeared which indicated that worry in both the GAD and non-anxious control group was associated with a reduction in HPV.

This tonic reduction in HRV in GAD and the phasic reduction during worry represents a breakdown of the inhibitory influences that allow for efficient self-regulation including the shifting of attentional focus. Thus an excitatory positive feedback loop is allowed to emerge.

GAD is characterized by excessive, unrealistic

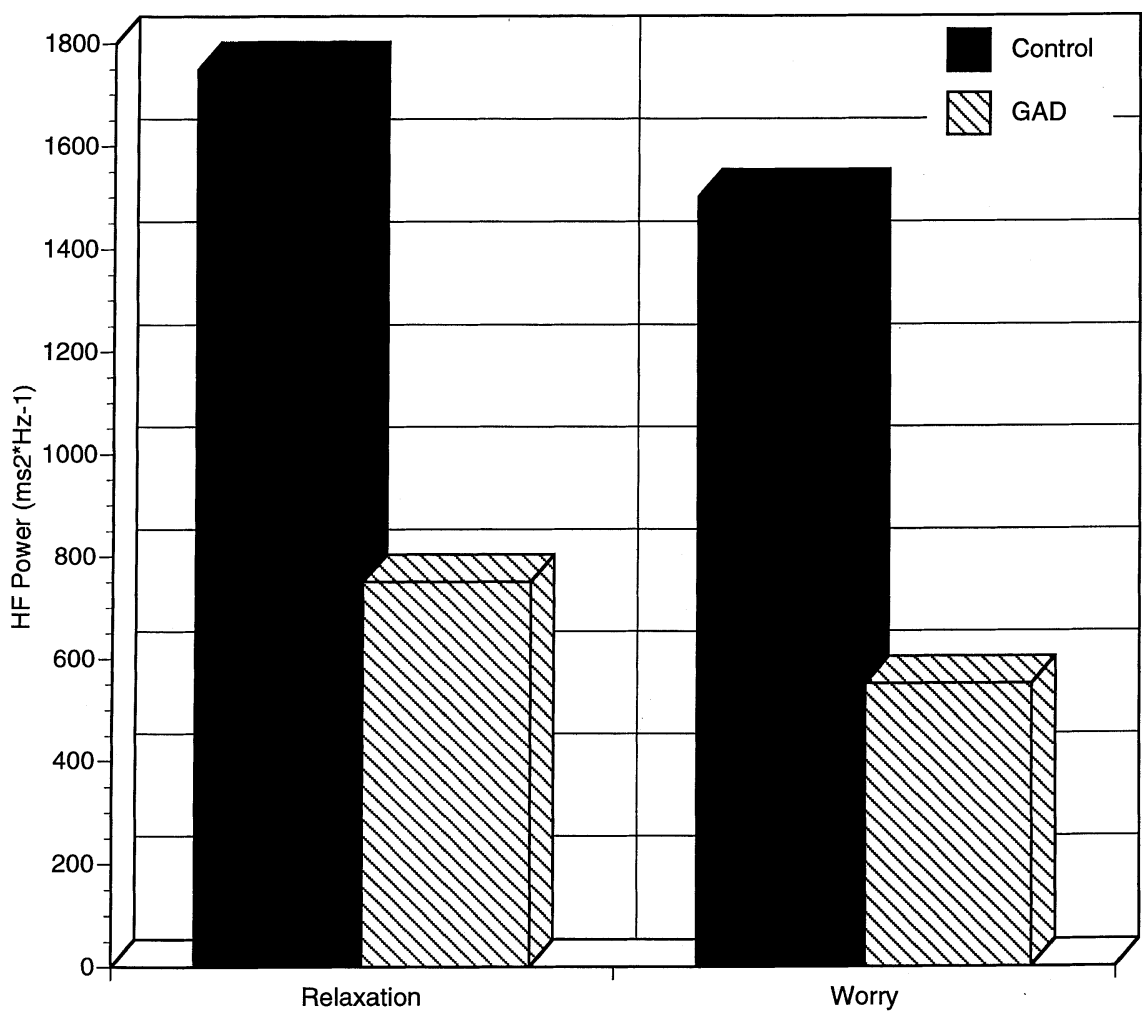

Fig. 1. High frequency spectral power for patients with generalized anxiety disorder (GAD) and non-anxious controls during relaxation and worry periods. Greater values indicate more vagally mediated HRV.

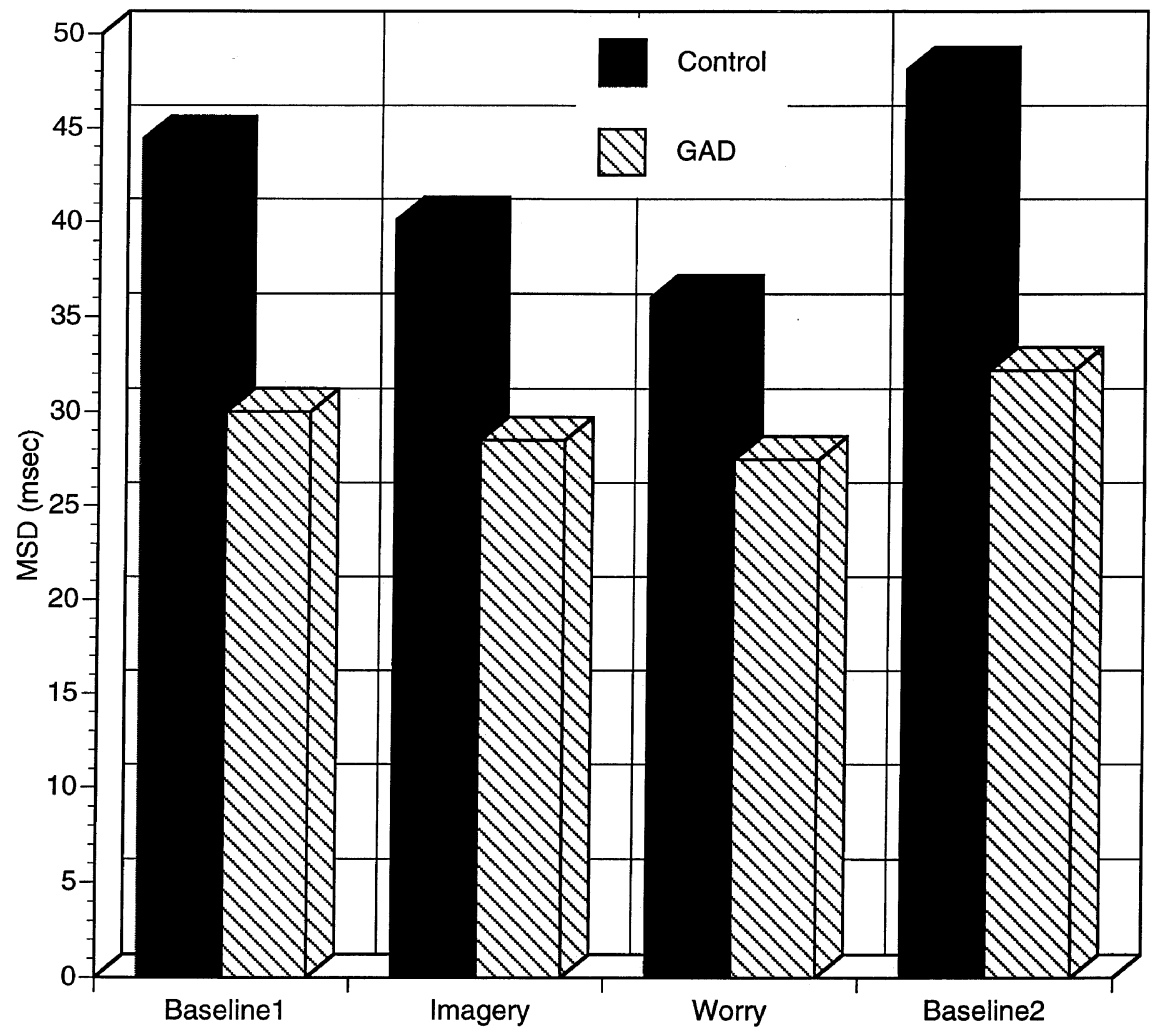

Fig. 2. Mean successive differences of cardiac period for chronic worriers (analogue GADs) and non-anxious controls during initial baseline, imagery, worry, and final baseline. Greater values indicate more vagally mediated HRV.

apprehension. This persistent state is supported by attentional mechanisms such as hypervigilance, scanning, and a pre-attentive bias for threat information (Mathews, 1990). As such, the normally fine-tuned ability to adjust to changing environmental factors becomes a rigid, inflexible response disposition.

Lyonfields et al. (1995) examined vagally mediated HPV in an analogue sample of GADs and a group of non-anxious controls during periods of imagery and worry about an individualized topic of concern. Resting baselines were recorded both at the beginning and at the end of the experimental session. The anxiety subjects showed reduced HPV across all recording periods with very little change from one period to the next (see Fig. 2). This was taken as evidence of a lack of behavioral flexibility and an inability to generate an appropriate response to the changing environmental demands. Whereas the non-anxious controls did show differences among the experimental conditions, the worry condition was associated with the greatest reduction in HPV. This reduction during worry was greater than the reduction during imagery of the same topic.

The ability to rapidly shift and effectively sustain attention in accord with situational demands is a critical component of self-regulation (Porges, 1992). The capacity to attend to salient events and disregard irrelevant ones greatly enhances the viability of an organism. Porges (1992) has proposed a two component model of attention, each component of which is associated with distinct cardiac activity. Specifically, vagally mediated changes in phasic heart rate (HR) are associated with reactive attention, whereas changes in HRV are associated with sustained attention. Reactive attention is thought to reflect a functional 'tuning' of the organism to novel stimuli to allow for the appropriate perception, evaluation, and response selection (Sokolov, 1963). Physiologically, reactive attention is associated with, among other things, cardiac deceleration. On the other hand, sustained attention is associated with vigilance and the suppression of HRV. The appropriate short-term suppression of HRV and the associated focusing of attention are important for effective self-regulation.

Tonic measures of HRV may reflect general responsiveness to changes in the internal and external environments and, as an index of neurovisceral integration, represents a measure of self-regulatory ability. Diminished tonic HRV and the associated reduction of vagally mediated cardiovascular control has been associated with a variety of pathological states and dispositions (see Friedman and Thayer,

1998; Malliani et al., 1991; Stein et al., 1994, for reviews). As a measure of vagally mediated cardiovascular activity, HRV is an index of a negative feedback mechanism that is important for the self-regulation of behavior. Vagal activity has negative cardiac chronotropic and dromotropic effects that serve to produce efficient cardiovascular functioning through the restraint of cardiac rate and electrical conduction speed. This restraint or inhibition is necessary for cardiac stability, responsiveness, and flexibility (Levy, 1990; Verrier, 1987).

The defensive attentional style that characterizes GAD is ultimately detrimental to functioning because it impairs the ability to flexibly respond to changing demands. It serves to reduce the range of behavioral options by limiting the ability of the organism to generate appropriate responses via a compromised ability to inhibit inappropriate responses. This impairment is evident even at a pre-attentive stage of information processing.

We have recently completed an experiment in which we attempted to provide support for this characterization of organism self-regulation through attentional control, affective information processing, and cardiac functioning. Thayer et al. (2000) examined phasic cardiac responses in an S1–S2 paradigm in persons with GAD and non-anxious controls. The S1–S2 paradigm has been extensively used to examine the relationship between phasic HR changes and attention. Briefly, this paradigm involves the presentation of a series of paired stimulus trials in which an initial cue stimulus (S1) is followed after a fixed inter-stimulus interval (ISI) by a second stimulus (S2). The second stimulus is usually imperative in some way. Classical conditioning is a type of S1–S2 procedure.

An extensive literature supports characteristic HR changes that accompany cognitive shifts over the course of the ISI and in response to the S2. A robust tri-phasic HR response has been described during the ISI (Bohlin and Kjelberg, 1979; Somsen et al., 1983). An initial HR deceleration following the S1 (D1) is followed by a HR acceleration (A1) over the next several cardiac beats. Finally, just prior to the S2 a second HR deceleration occurs (D2). Each of these phasic cardiac changes has been associated with specific cognitive activities. The D1 has generally been interpreted as an orienting response which 'tunes' the organism to novel stimuli (Sokolov, 1963). The A1 has been associated with a number of functions including stimulus elaboration, the signal value of S1 and the response requirements of S2. The D2 component is the most robust and has been associated with the anticipation of S2. This component has been shown to be enhanced (greater deceleration) prior to an aversive S2 (Somsen et al., 1983).

The stimulus value of the S2 determines the cardiac response subsequent to the S2. For a neutral or appetitive stimulus, a HR deceleration is elicited. Importantly, for an aversive stimulus HR acceleration is elicited. This is thought to be associated with a defensive reaction (DR) which serves to buffer the impact of the aversive stimulus on the organism (Hare and Blevings, 1975). Jennings (1986) has suggested that this HR acceleration may represent motivated inattention and a cognitive avoidance of threatening information. Of particular note for our model, these phasic cardiac changes found in the S1–S2 paradigm have been shown to be vagally mediated (Porges, 1992; Somsen et al., 1983).

Porges (1992) has noted that cardiac vagal tone is positively associated with the ability to self-regulate via the modulation of attention. Given our previous findings of reduced vagal activity in GAD (Lyonfields et al., 1995; Thayer et al., 1996) and the reports of dysfunctional attention in GAD (Mathews, 1990), we reasoned that non-anxious controls would have better attentional regulation relative to persons with GAD. This would manifest itself by individuals with GAD vigilantly monitoring their environment for threat and failing to disengage from unimportant events. Thus, persons with GAD would fail to habituate to novel neutral stimuli whereas non-anxious controls would show habituation.

Porges (1992) has also suggested that the magnitude of cardiac responses is positively related to vagal tone. Therefore, relative to persons with GAD, non-anxious controls would show initially larger orienting responses (OR), even though they would show rapid habituation over time. This is consistent not only with the reduced vagal tone found in GADs but also with the restricted range of autonomic responses found in GADs (Hoehn-Saric and McLeod, 1988). Thus, across individuals, resting vagal tone should be positively associated with the

magnitude of the OR. Given that the OR is important for appropriate perception, evaluation and response selection (Sokolov, 1963), a robust OR is an indication of a healthy and adaptive engagement of the organism with its environment.

The direction of phasic cardiac responses is also of importance. Cardiac decelerations, as noted above, are associated with the OR and with anticipation of important events (Berg and Donohue, 1992). Cardiac accelerations are associated with the DR, stimulus elaboration, or motivated inattention (Jennings, 1986). Therefore, we also predicted that persons with GAD would show defensive reactions to threat words and that this response would not habituate. Non-anxious controls, on the other hand, would show an OR in early trials but would eventually show habituation. In addition, GADs would develop a conditioned anticipatory HR deceleration to threat words. This prediction is based on the findings of an attentional bias toward threat cues in GAD, an associative-learning basis for the generalization of threatening information that characterizes GAD, and the phasic HR deceleration found in anticipation of aversive stimuli found in non-anxious individuals (Somsen et al., 1983). The verbal-linguistic processing that is characteristic of worry and GAD supports this last supposition. Thus, verbal stimuli are perceived as aversive to these individuals.

The results were consistent with our predictions and were indicative of attentional dysregulation in the GAD group as indicated by vagally mediated phasic HR changes. First, relative to non-anxious controls, persons with GAD showed smaller cardiac ORs, impaired habituation to neutral words, and the development of a conditioned anticipatory HR deceleration to threat words. A natural response to potential threat is the deployment of attention to scan the environment for relevant cues and anticipate and evaluate the risk. Early detection of threat-related cues allows an organism to either prepare an effective escape or modulate the impact of the danger. In GAD, the threat is imagined, and immediate behavioral responses are precluded. Rather, protracted scanning and hypervigilance are maintained to guard against a perpetually threatening environment. To the individual with GAD, the world is perceived as a very dangerous place. Moreover, the inability to inhibit the allocation of attention to innocuous stimuli leads to a positive feedback loop that in essence spirals out of control. This leads to a very broad basin of attraction for the 'worry' attractor. That is, worry becomes the preferred response to an ever-widening range of situations.

Persons with GAD also showed HR acceleration to threat words and this response did not habituate. Persons with chronic anticipatory anxiety have been shown to respond to perceived threat with HR acceleration that is characteristic of the defensive response (Graham and Clifton, 1966; Hare and Blevings, 1975; Sokolov, 1963). The sustained attention that is associated with vigilance is accompanied by a phasic decrease in HR variability (Porges, 1992). However, this garnering of attentional resources constrains the ability to respond flexibly to environmental challenges. The finding that persons with GAD respond to threat-related words with HR accelerations fits the above characterization and extends our previous findings of reduced HR variability in GAD. This response may represent an attempt to shield against the impact of threat, as is suggested for the DR elicited by electric shock in non-anxious individuals (Somsen et al., 1983), or as is observed in phobic reactions to relevant specific stimuli (Hare and Blevings, 1975). An alternative interpretation is that the HR acceleration is indicative of motivated inattention (Jennings, 1986), and thus may be a form of cognitive avoidance of threat by persons with GAD. Stormark and colleagues (Stormark et al., 1995; Stormark and Hugdahl, 1996) have recently demonstrated the finding of an attentional bias *toward* threat information with a subsequent attempt to *avoid* processing of such information. He concludes that the same emotionally relevant stimulus can promote both initial attentional orienting and subsequent avoidance given the necessary appetitive and aversive motivational processes. Thus, ''emotional modulation of attention involves . . . both the engagement and disengagement components of attentional orienting.'' (Stormark, 1996, p. 23).

The failure to habituate to harmless stimuli engenders a rigid response disposition based upon the defensive reaction. Again, this cardiac acceleration is the opposite of the cardiac deceleration associated with the detection and evaluation of novel stimuli that allows for appropriate response selection. Indeed, evidence exists that anxiety and stress can

decrease the number of alternative options that are explored thus further reducing the chance that disconfirming evidence from the environment will be detected and processed (Keinan et al., 1987). This positive feedback loop serves, in part, to maintain disorders of affect in the face of the large amounts of disconfirming data the individual is apt to encounter in their daily living. However, the perceived threatening nature of the environment may lead to a restriction of behavior such that the individual is exposed to less and less novel, disconfirming information. This again is a positive feedback mechanism that perpetuates the existing, dysfunctional state. The prototypical example of such restricted behavior is seen in agoraphobia where the individual can no longer leave their own home.

These mechanisms could operate at a precognitive or preconscious level. This is consistent with classic work on perceptual defense (see Mackinnon and Dukes, 1962, for a review) and the psychophysiology of attention (e.g., Graham and Clifton, 1966; Sokolov, 1963), as well as more contemporary notions of pre-attentive discrimination and selective processing of threat in anxiety (Mathews, 1990). Furthermore, this rapid mobilization of resources for action is consistent with the dynamical systems characterization of emotion as an emergent response or attractor driven by motivational factors (see Globus and Arpaia, 1994; similar ideas have been expressed outside of the dynamical systems framework by Lang et al., 1992).

Our data provide further support that these processes are supported by cardiac activity. The CAN, and related functional networks, such as the rostral limbic system, provide the mechanism by which cardiac, affective, attentional, and behavioral responses are coordinated in the service of goal-directed behavior and adaptability. Moreover, the characteristics of this network are consistent with our view that emphasizes the importance of negative feedback circuits and inhibitory processes. The CAN and related frontal-subcortical circuits (Masterman and Cummings, 1997; Spyer, 1989) are reciprocally interconnected functional units composed of parallel pathways. These functional units are neurochemically complex with glutamate and GABA being the main excitatory and inhibitory neurotransmitters, respectively (Benarroch, 1997). For example, Masterman and Cummings (1997) identified several frontal-subcortical circuits and note that these circuits are modulated via two opposing parallel pathways connecting the striatum to output nuclei in the basal ganglia (see Fig. 3). They describe these pathways as follows. Excitatory, glutamateric projections from the frontal cortex to specific areas within the striatum form the front-end of both pathways. These pathways then diverge via striatal output neurons in the caudate, putamen, and ventral striatum into a direct pathway and an indirect pathway. The direct pathway is formed by inhibitory GABA fibers in the striatum to neurons in the internal segment of the globus pallidus and the substantia nigra pars reticulata. From here, GABAergic efferents are sent to discrete areas in the thalamus, which relay information back to the same discrete areas of the frontal cortex. The indirect pathway is formed by GABAergic outputs to the globus pallidus externa, GABAergic fibers to the subthalamic nucleus, and glutamateric projections to the internal segment of the globus pallidus and the substantia nigra pars reticulata. These areas then project to specific thalamic targets that again relay information back to the circuits' origins in the frontal cortex.

Importantly, the two striatal efferent projections have opposing influences on the output neurons from the internal segment of the globus pallidus and the substantia nigra pars reticulata which in turn have opposing influences on the thalamo-cortical feedback circuits. The direct pathway excites the thalamus

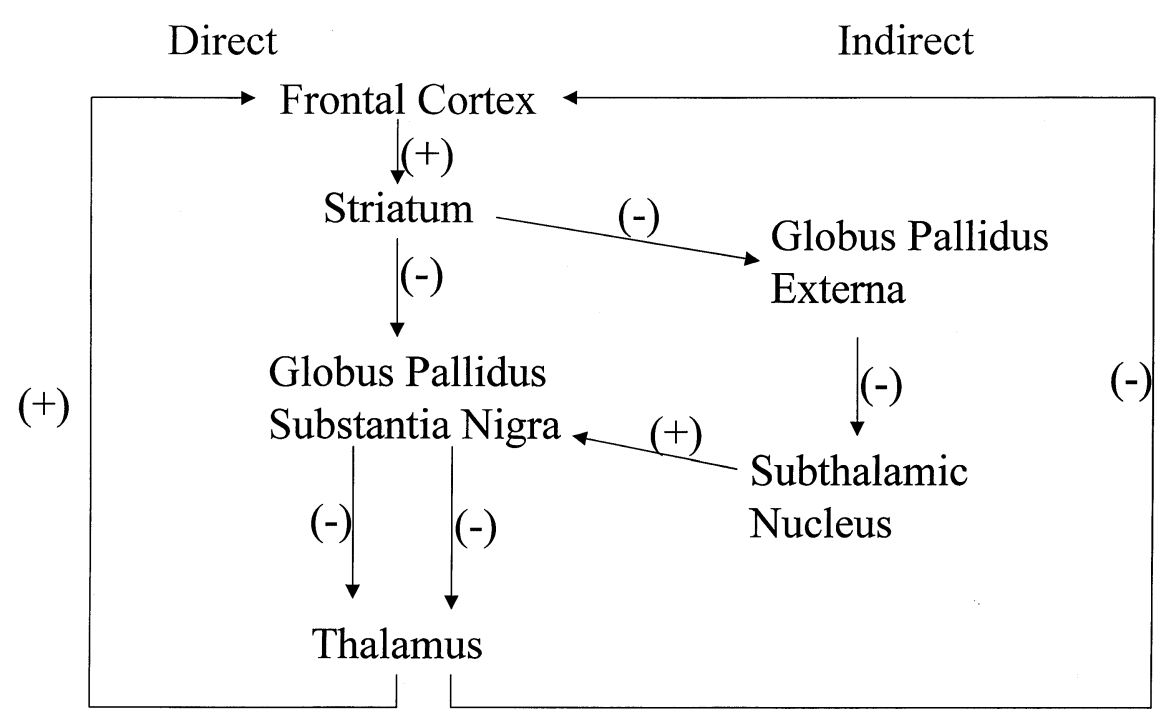

Fig. 3. Schematic diagram of the direct and indirect pathways associated with frontal-subcortical circuits identified by Masterman and Cummings (1997). Excitatory (+) and inhibitory (−) paths are identified. See text for details.

whereas the indirect pathway inhibits the thalamus. A tonic GABA-mediated inhibition of the thalamus is produced by output neurons from the basal ganglia. The direct pathway serves to disinhibit (excite) the thalamus via inhibitory striatal efferents to the internal segment of the globus pallidus and the substantia nigra pars reticulata. The indirect pathway serves to inhibit the thalamus via disinhibition of the tonically inhibited subthalamic nucleus that sends excitatory output to the internal segment of the globus pallidus and the substantia nigra pars reticulata. Reduced variability in attention, affect, cardiac, and motor behavior has been linked to disruption of these feedback circuits (Masterman and Cummings, 1997; Spyer, 1989).

5. Neural imaging of emotion and attention

Advances in our ability to observe the brain in action have shed much light on the neural substrates of behavior. In a recent comprehensive review of both the animal and human literature, Devinsky et al. (1995) propose a system of interconnected areas, termed the anterior executive region, that ''assesses the motivational content of internal and external stimuli and regulates context-dependent behaviours'' (p. 279). The anterior cingulate has been singled out of this larger system of structures that includes the amygdala, the periaquaductal gray, ventral striatum, orbitofrontal and anterior insular cortices. They conclude that the ''anterior cingulate cortex appears to play a crucial role in initiation, motivation, and goal-directed behaviours.'' (p. 279). These conclusions are based upon the role of the anterior cingulate in 'executive behavior' and its projections into motor areas associated with visceral control. Affect, cognition, pain, social interactions, response selection, and autonomic activity are some of the functions associated with the anterior cingulate cortex. Together, they help to guide the organism through its environment in relative safety. Numerous studies have indicated that damage to the anterior cingulate is associated with a wide variety of dysfunctions including altered attention, flattened affect, autonomic dysfunctions such as tachycardia, inappropriate social behavior, and poor associative learning. Clinical syndromes or disorders associated with damage to or dysfunction of the anterior cingulate include akinetic mutism, certain forms of epilepsy, Tourette syndrome, schizophrenia, depression, anxiety, obsessive-compulsive disorder, and aggression.

That the cingulate cortex is central to emotion is not new. At least as early as 1937, Papez (1937) proposed that the cingulate cortex was an integral part of a neural network or circuit that formed the neuroanatomical basis of emotional experience. However, most recent work has focused on the role of the cingulate cortex in information processing (Cabeza and Nyberg, 1997).

We propose that the cingulate cortex, specifically the anterior cingulate, serves as a point of integration for visceral, attentional, and affective information that is critical for self-regulation and adaptability. This area appears to be associated with the conscious allocation of attention, intrinsic to which is greater inhibition of irrelevant information. Moreover, we feel that the evidence supports a central role for the anterior cingulate in response selection due to its function as a source of negative feedback, via vagal pathways, to the cardiovascular system. Recent neuroimaging and neuroanatomical studies have suggested that the anterior cingulate is comprised of three interactive regions. The rostral and ventral regions are associated with affective, motivated, and autonomic behavior, whereas the dorsal region is associated with response selection as well as pain. Mayberg (1997) has proposed that the rostral cingulate is particularly important for the integration of visceral, attentional, and affective information. As such, this area may play a critical role in the coordination of the complex mix of cognitive, affective, behavioral, and physiological concomitants of normal affective states and dispositions. In the following, we will briefly overview the literature on the role of the anterior executive region, particularly the anterior cingulate cortex, in emotion and attention. Then we will review some of our work on the emotional, attentional, and autonomic correlates of activity of the CAN and the anterior executive region as revealed by positron emission tomography (PET) studies.

Reiman (1997) reviewed six studies in which PET was used to investigate normal and pathological emotions. A number of the areas within the anterior executive region appeared to be associated with

affective behavior. Of particular note for our studies of anxiety disorders, the anterior cingulate was involved with both normal and pathological forms of anxiety. Specifically, Reiman states that the anterior cingulate ''may participate in the conscious experience of emotion, the attentional or behavioral response to an anxiety-provoking situation, *the inhibition* of excessive emotion, or the process of monitoring the individual's emotional state in order to make a personally relevant decision'' (italics added, p. 9). In addition, it has been reported that the anterior cingulate is involved in the processing of both positive (Dolan et al., 1996) and negative (Morris et al., 1998) facial expressions of emotion.

Viewing emotional pictures has become a very fruitful technique in the experimental investigation of emotion. Lane et al. (1998a) used this technique to examine cortical activation during selective attending to subjective emotional responses. Lane et al. (1998a) had 10 healthy male participants focus their attention on the emotional valence of a subset of pictures from the International Affective Picture System (Lang et al., 1995) or focus on the setting (indoors, outdoors, or neither) of a subset of the same pictures. The former condition was thought to be associated with an internal focus of attention, whereas the latter condition was thought to be associated with an external focus of attention. Twelve PET derived measures of cerebral blood flow were obtained from each participant. Six were recorded during the internal focus condition and six were recorded during the external focus condition. The internal and external focus conditions were counterbalanced across subjects. Results indicated that during the external focus condition blood flow increased bilaterally in the parieto-occipital cortex (Brodmann's area (BA) 19/37 and BA 39). Importantly, during the internal focus condition blood flow significantly increased maximally in the anterior cingulate (BA 32) with secondary activations in the medial prefrontal cortex (BA 9), right temporal pole (BA 38) extending into the frontal operculum and insula, and of lesser magnitude in the ventral cingulate. These results support the early work of Papez that identified the anterior cingulate as central to the 'experience' of emotion. Moreover, they are consistent with the review of Reiman (1997) implicating the anterior cingulate in the conscious experience of emotion.

The regulation of attention has also been linked to the anterior cingulate. The anterior executive region, particularly the anterior cingulate, has been implicated in an anterior attentional system associated with attentional shifting and response selection (Posner and Petersen, 1990). Cabeza and Nyberg (1997) recently reviewed the PET studies on cognition in normal subjects. They report that several studies investigating attention have found activation in the anterior cingulate during tasks that involve competition between processing alternatives and inhibition of competing responses. Thus, the anterior cingulate seems to be involved in response selection and self-regulation via modulation of competing system inputs and outputs. This response selection and self-regulation may extend to social interactions. Devinsky et al. (1995) note in their review that damage to the anterior cingulate has been associated with a host of social affective impairments including blunted affect, impulsivity, disinhibition, and poor social judgement (p. 291).

Lane and Schwartz (1987) put forward a novel idea concerning a cognitive-developmental model of social affective behavior. Their model of 'levels of emotional awareness' treats the ability to make socially appropriate judgements as an individual difference in the capacity to experience emotion in a complex and differentiated manner. This ability involves the selection of relevant social information and the inhibition of socially inappropriate cues and responses. Lane et al. (1998b) recently investigated the neural correlates of levels of emotional awareness using PET. They correlated scores on the Levels of Emotional Awareness Scale (LEAS; Lane et al., 1990) with cerebral blood flow during film- and recall-induced emotions in a group of 12 healthy women. The results suggested that the anterior cingulate (BA 24) was associated with the accurate detection of interoceptive and exteroceptive emotional signals. These findings implicate the anterior cingulate in the response selection processes necessary for the experiential processing and response generation related to social affective information.

Whereas the anterior cingulate has been highlighted here, it is clear that the anterior cingulate is just one area in the larger CAN and rostral limbic system responsible for the central underpinnings of goal-directed behavior and adaptability. Vagally mediated cardiac activity has been linked to attentional

regulation and affective behavior. We (Lane and Thayer, unpublished data) have recently investigated the neural correlates of heart rate variability. An index of vagally mediated heart rate variability was correlated with cerebral blood flow. The single highest correlation between heart rate variability and cerebral blood flow occurred in the insular cortex. A secondary association was found between heart rate variability and a subcortical region that included the thalamus. The association of these two areas with heart rate variability is consistent with the neural feedback circuits that comprise the CAN and the associated rostral limbic system. The primary output of the CAN is modulated via the preganglionic sympathetic and parasympathetic neurons. These same neurons via input to the sino-atrial node are responsible for the complex variability that characterizes a healthy and adaptive cardiac time series. Thus, neural imaging data supports a direct association between activity of the CAN and heart rate variability.

6. Summary

We have presented evidence that autonomic control of the heart as measured by HRV is related to attentional regulation, affective information processing, physiological flexibility, and cerebral blood flow. A relative deficit in vagally mediated HRV was found in individuals diagnosed with GAD. Importantly, HRV was also decreased in both non-anxious controls and patients during worry. Anxiety disorders have been linked to excess cardiovascular morbidity and mortality (Hayward, 1995). Traditionally, anxiety has been associated with increased arousal and sympathetic nervous system activity. However, we have suggested that the relative increase in arousal observed in various anxiety disorders is due to a deficit in the inhibitory activity of the parasympathetic nervous system (Friedman and Thayer, 1998). Thus, the relative increase in arousal is a dis-inhibition of sympathoexcitatory mechanisms of cardiac control.

In addition, the decrease in vagally mediated HRV during worry highlights the importance of verbal-linguistic information processing. Kubzansky et al. (1997) have recently shown that chronic worry is related to an increased risk of coronary heart disease. Thus, the perseverative mode of thinking that is characteristic of worry that can lead to increased anxiety is also associated with an increased risk for cardiovascular disease. We have also demonstrated that individuals that worry a significant portion of the day show less physiological flexibility and adaptability to changing task demands.

The poor orienting to novel stimuli, poor habituation to innocuous words, conditioned anticipatory HR deceleration to threat words, and the HR acceleration to the presentation of threat words represents a dysfunction of attentional and affective information processing that does not allow for the appropriate response to environmental demands. This inability to disengage the threat detection or Defensive Behavior system serves to perpetuate arousal and anxiety even when no real threat exists. Thus, individuals with low HRV are less able to detect and experience 'safety' when it is in fact present. We (Sollers et al., 1997) have recently shown that individuals with low HRV experience a blunting of subjective emotional reactions. Thus, low HRV is associated with poor affective information processing.

Importantly, we have shown elsewhere that following intervention with cognitive-behavioral therapy, a small sample of GAD patients made significant changes in the direction of increasing vagal cardiac control, with concomitant elevations in HR variability and reductions in anxiety, symptamotology, and HR (Friedman et al., 1993). We are continuing to follow these individuals, as well as additional patients, and now have 2-year follow-up data on a larger sample with similar findings.

We have also presented evidence that links HRV to the CAN and related neural circuits. Whereas these preliminary results are promising and consistent with our model, additional studies are needed to further specify the nature of this relationship. Additional psychophysiological and neuroimaging studies using other attentional and cognitive tasks, different aspects of affective information processing and expression, and further investigations of gender differences in HRV, cognition, and affect are currently underway.

We have shown that the neural circuits identified by other researchers to underlie autonomic regulation (Benarroch, 1997), attentional regulation (Devinsky et al., 1995), and affective regulation (Damasio,

1998) show substantial structural overlap. These various circuits most likely reflect a single structural unit that subserves numerous functions associated with goal-directed behavior and adaptability. As such, it forms the basis for an integrative self-regulation system (Benarroch, 1997).

7. Conclusions

Autonomic regulation, attentional regulation, and affective regulation allow an organism to meet the challenges of an ever-changing environment. However, the importance of inhibitory processes in this self-regulatory behavior has not yet made its way into the dominant thinking in this area. From a systems perspective, inhibitory processes can be viewed as negative feedback circuits that allow for the interruption of ongoing behavior and the re-deployment of resources to other tasks. When these negative feedback mechanisms are compromised, positive feedback loops may develop as a result of dis-inhibition. These positive feedback loops promote perseveration and continued activation of systems thereby limiting their availability for other processes. These positive feedback loops can have disastrous consequences. For example, at the level of the synapse, McGeer et al. (1978, p. 134) note that ''. . . substances which interfere with inhibitory synaptic action would cause unfettered excitatory action of neuron onto neuron and so lead to convulsions.''

Healthy systems involve both positive and negative feedback circuits (Glass and Mackey, 1988; Goldberger, 1992). The cognitive and affective perseveration seen in GAD as exemplified by worry can be seen as a positive feedback circuit for which the related negative inhibitory circuit is dysfunctional. Appropriate response selection involves the active inhibition of the allocation of resources to irrelevant information and processes. That this inhibitory circuit may be indexed by cardiac vagal tone has several implications. First, vagal influences on the cardiovascular system represent negative chronotropic and dromotropic mechanisms that are associated with system flexibility, responsivity, and stability (Levy, 1990; Porges, 1992). Second, as an index of central–peripheral neural feedback mechanisms, cardiac vagal tone represents a psychophysiological resource that the organism can bring to bear, as it were, on an environmental challenge (Friedman and Thayer, 1998). Third, framing the diverse self-regulatory functions and dysfunctions observed in terms of vagal processes as opposed to sympathetic processes may be a more parsimonious representation of the data (Friedman and Thayer, 1998; Thayer and Friedman, 1997). From this perspective, the relative sympathetic activation seen in anxiety disorders may represent *dis-inhibition* due to faulty inhibitory mechanisms.

In sum, we have presented a model of neurovisceral integration in emotion regulation and dysregulation that stresses inhibitory processes. We have placed this model in a systems framework involving the interplay among positive and negative feedback circuits that allows for the parsimonious explication of affective behavior. These systems are active in the service of goal-directed and motivated behavior. As such, they might provide the underpinnings of a comprehensive model of the cognitive, affective, behavioral, and physiological concomitants of normal and pathological emotional states and dispositions.

Acknowledgements

We would like to thank Bruce Friedman, Tom Borkovec, Jos Brosschot, Gary Schwartz, Peter Lang, and Jack Maser for their valuable contributions.

References

Benarroch, E.E., 1993. The central autonomic network: functional organization, dysfunction, and perspective. Mayo Clin. Proc. 68, 988–1001.

Benarroch, E.E., 1997. The central autonomic network. In: Low, P.A. (Ed.), Clinical Autonomic Disorders, 2nd Edition. Lippincott-Raven, Philadelphia, pp. 17–23.

Berg, W.K., Donohue, R.L., 1992. Anticipatory processes in infants: cardiac components. In: Campbell, B.A., Hayne, H., Richardson, R. (Eds.), Attention and Information Processing in Infants and Adults. Erlbaum, Hillside, NJ, pp. 61–80.

Bohlin, G., Kjelberg, A., 1979. Orienting activity in two-stimulus paradigms as reflected in heart rate. In: Kimmel, H.D., van Olst, E.H., Orlebeke, J.F. (Eds.), The Orienting Reflex in

Humans. Lawrence Erlbaum Associates, Hillsdale, NJ, pp. 169–197.

Brosschot, J.F., Thayer, J.F., 1998. Anger inhibition, cardiovascular recovery, and vagal function: a model of the link between hostility and cardiovascular disease. Ann. Behav. Med. 20, 1–8.

Cabeza, R., Nyberg, L., 1997. Imaging cognition: an empirical review of PET studies with normal subjects. J. Cogn. Neurosci. 9, 1–26.

Damasio, A.R., 1998. Emotion in the perspective of an integrated nervous system. Brain Res. Rev. 26, 83–86.

Devinsky, O., Morrell, M.J., Vogt, B.A., 1995. Contributions of anterior cingulate cortex to behavior. Brain 118, 279–306.

Dolan, R.J., Fletcher, P., Morris, J., Kapur, N., Deakin, J.F., Frith, C.D., 1996. Neural activation during covert processing of positive facial expressions. Neuroimage 4, 194–200.

Friedman, B.H., Thayer, J.F., 1998. Autonomic balance revisited: panic anxiety and heart rate variability. J. Psychosom. Res. 44, 133–151.

Friedman, B.H., Thayer, J.F., Borkovec, T.D., 1993. Heart rate variability in generalized anxiety disorder [abstract]. Psychophysiology 30, S28.

Frijda, N., 1988. The laws of emotion. Am. Psychol. 43, 349–358.

Glass, L., Mackey, M.C., 1988. From Clocks To Chaos. Princeton University Press, Princeton, NJ.

Globus, G.G., Arpaia, J.P., 1994. Psychiatry and the new dynamics. Biol. Psychiatry 35, 352–364.

Goldberger, A.L., 1992. Applications of chaos to physiology and medicine. In: Kim, J.H., Stringer, J. (Eds.), Applied Chaos. Wiley, New York, pp. 321–331.

Graham, F.K., Clifton, R.K., 1966. Heart-rate change as a component of the orienting response. Psychol. Bull. 65, 305–320.

Hare, R.D., Blevings, G., 1975. Conditioned orienting and defensive responses. Psychophysiology 12, 89–97.

Hayward, C., 1995. Psychiatric illness and cardiovascular disease risk. Epidemiol. Rev. 17, 129–138.

Heilman, K.M., 1997. The neurobiology of emotional experience. J. Neuropsychiatry Clin. Neurosci. 9, 439–448.

Hoehn-Saric, R., McLeod, D.R., 1988. The peripheral sympathetic nervous system: its role in pathological anxiety. Psychiatr. Clin. North Am. 11, 375–386.

James, W., 1997. In: Gazzaniga, M.S. (Ed.), Conversations in the Cognitive Neurosciences. MIT Press, Cambridge, MA.

Jennings, J.R., 1986. Bodily changes during attention. In: Coles, M.G.H., Donchin, E., Porges, S.W. (Eds.), Psychophysiology: Systems, Processes, and Applications. Guilford Press, New York, pp. 268–289.

Johnsen, B.H., Thayer, J.F., Hugdahl, K., 1995. Affective judgment of the Ekman faces: a dimensional approach. J. Psychophysiol. 9, 193–202.

Keinan, G., Friedland, N., Ben-Porath, Y., 1987. Decision making under stress: scanning of alternatives under physical threat. Acta Psychol. 64, 219–228.

Kubzansky, L.D., Kawachi, I., Spiro, A., Weiss, S.T., Vokonas, P.S., Sparrow, D., 1997. Is worrying bad for your heart? A prospective study of worry and coronary heart disease in the Normative Aging Study. Circulation 95, 818–824.

Lane, R.D., Fink, G.R., Chau, P.M., Dolan, R.J., 1998a. Neural activation during selective attention to subjective emotional responses. Neuroreport 8, 3969–3972.

Lane, R.D., Reiman, E.M., Axelrod, B., Yun, L.-S., Holmes, A., Schwartz, G.E., 1998b. Neural correlates of levels of emotional awareness: evidence of an interaction between emotion and attention in the anterior cingulate cortex. J. Cogn. Neurosci. 10 (4), 525–535.

Lane, R.D., Schwartz, G.E., 1987. Levels of emotional awareness: a cognitive-developmental theory and its application to psychopathology. Am. J. Psychiatry 144, 133–143.

Lane, R.D., Quinlan, D., Schwartz, G.E., Walker, P., Zeitlin, S., 1990. The levels of emotional awareness scale: a cognitive-developmental measure of emotion. J. Pers. Assess. 55, 124–134.

Lang, P.J., Bradley, M.M., Cuthbert, B.N., 1992. A motivational analyses of emotion: reflex–cortex connections. Psychol. Sci. 3, 44–49.

Lang, P.J., Bradley, M.M., Cuthbert, B.N., 1995. International Affective Picture System (IAPS): Technical Manual and Affective Ratings. University of Florida, Center for Research in Psychophysiology, Gainsville.

Levenson, R.W., 1988. Emotion and the autonomic nervous system: a prospectus for research on autonomic specificity. In: Wagner, H.L. (Ed.), Social Psychophysiology and Emotion: Theory and Clinical Applications. Wiley, Chichester, pp. 17–42.

Levy, M.N., 1990. Autonomic interactions in cardiac control. Ann. New York Acad. Sci. 601, 209–221.

Lyonfields, J., Borkovec, T.D., Thayer, J.F., 1995. Vagal tone in generalized anxiety disorder and the effects of aversive imagery and worrisome thinking. Behav. Ther. 26, 457–466.

Mackinnon, D.W., Dukes, W., 1962. Repression. In: Postman, L. (Ed.), Psychology in the Making: Histories of Selected Research Problems. Alfred A. Knopf, New York, pp. 662–744.

Malliani, A., Pagani, M., Lombardi, F., Cerutti, S., 1991. Cardiovascular neural regulation explored in the frequency domain. Circulation 84, 482–492.

Masterman, D.L., Cummings, J.L., 1997. Frontal-subcortical circuits: the anatomical basis of executive, social and motivated behaviors. J. Psychopharmacol. 11, 107–114.

Mathews, A., 1990. Why worry? The cognitive function of anxiety. Behav. Res. Ther. 28, 455–468.

Mayberg, H.S., 1997. Limbic-cortical dysregulation: a proposed model of depression. J. Neuropsychiatry Clin. Neurosci. 9, 471–481.

McGeer, P.L., Eccles, J.C., McGeer, E.G., 1978. Molecular Neurobiology of the Mammalian Brain. Plenum Press, New York.

Miller, M.L., Thayer, J.F., 1988. On the nature of self-monitoring: relations to adjustment and identity. Pers. Soc. Psychol. Bull. 14, 544–553.

Miller, M.L., Thayer, J.F., 1989. On the existence of discrete classes in personality: is self-monitoring the correct joint to carve? J. Pers. Soc. Psychol. 57, 143–155.

Morris, J.S., Friston, K.J., Buchel, C., Frith, C.D., Young, A.W., Calder, A.J., Dolan, R.J., 1998. A neuromodulatory role for the

amygdala in processing emotional facial expressions. Brain 121, 47–57.

Nyklicek, I., Thayer, J.F., van Doornen, L.J.P., 1997. Cardiorespiratory differentiation of musically-induced emotions. J. Psychophysiol. 11, 780–790.

Osgood, C., Suci, G., Tannenbaum, P., 1957. The Measurement of Meaning. University of Illinois, Urbana, IL.

Öhman, A., Esteves, F., Flykt, A., Soares, J.F., 1993. Gateways to consciousness: emotion, attention, and electrodermal activity. In: Roy, J.-C., Boucsein, W., Fowles, D.C., Gruzelier, J.H. (Eds.), Progress in Electrodermal Research. Plenum Press, New York.

Papez, J.W., 1937. A proposed mechanism of emotion. Arch. Neurol. Psychiatry 38, 725–734.

Paramananda, S., 1974. Concentration and Meditation, 8th Edition. Vedanta Press, Hollywood, CA.

Porges, S.W., 1991. Vagal tone: an autonomic mediator of affect. In: Barber, J., Dodge, K.A. (Eds.), The Development of Emotion Regulation and Dysregulation. Cambridge University Press, Cambridge, pp. 111–128.

Porges, S.W., 1992. Autonomic regulation and attention. In: Campbell, B.A., Hayne, H., Richardson, R. (Eds.), Attention and Information Processing in Infants and Adults. Erlbaum, Hillside, NJ, pp. 201–223.

Posner, M.I., Petersen, S., 1990. The attentional system of the human brain. Annu. Rev. Neurosci. 13, 25–42.

Reiman, E.M., 1997. The application of positron emission tomography to the study of normal and pathological emotion. J. Clin. Psychiatry 58 (Suppl.), 4–12.

Richards, J.R., Casey, B.J., 1992. Development of sustained visual attention in the human infant. In: Campbell, B.A., Hayne, H., Richardson, R. (Eds.), Attention and Information Processing in Infants and Adults. Erlbaum, Hillside, NJ, pp. 30–60.

Russell, J.A., 1980. A circumflex model of affect. J. Pers. Soc. Psychol. 39, 1161–1178.

Saul, J.P., 1990. Beat-to-beat variations of heart rate reflect modulation of cardiac autonomic outflow. News Physiol. Sci. 5, 32–37.

Schneirla, T.C., 1959. An Evolutionary and Developmental Theory of Biphasic Processes Underlying Approach and Withdrawal. Nebraska Symposium On Motivation, Vol. 1959. University of Nebraska Press, Lincoln, NE, 142 pp.

Schwartz, G.E., 1986. Emotion and psychophysiological organization: a systems approach. In: Coles, M.G.H., Donchin, E., Porges, S.W. (Eds.), Psychophysiology: Systems, Processes, and Applications. Guilford Press, New York, pp. 354–377.

Sokolov, E.N., 1963. Perception and the Orienting Response. MacMillan, New York.

Sollers, J.J., Mueller, C.A., Thayer, J.F., 1997. Emotional responses, heart period variability, and physical activity. Psychosom. Med. 59, 93, abstract.

Somsen, R.J.M., van der Molen, M.W., Orlebeke, J.F., 1983. Phasic heart rate changes in reaction time, shock avoidance, and unavoidable shock tasks: are hypothetical generalizations about different S1–S2 tasks justified? Psychophysiology 20, 88–94.

Spyer, K.M., 1989. Neural mechanisms involved in cardiovascular control during affective behavior. Trends Neurosci. 12, 506–513.

Sroka, K., Peimann, C.J., Seevers, H., 1997. Heart rate variability in myocardial ischemia during daily life. J. Electrocardiol. 30, 45–56.

Stein, P.K., Bosner, M.S., Kleiger, R.E., Conger, B.M., 1994. Heart rate variability: a measure of cardiac autonomic tone. Am. Heart J. 127, 1376–1381.

Stormark, K.M., 1996. Emotional modulation of selective attention: experimental and clinical evidence. Unpublished doctoral dissertation, University of Bergen, Norway.

Stormark, K.M., Hugdahl, K., 1996. Peripheral cuing of covert spatial attention before and after emotional conditioning of the cue. Int. J. Neurosci. 86, 225–240.

Stormark, K.M., Laberg, J.C., Nordby, H., Hugdahl, K., 1998. Heart rate responses indicate locked-in attention in alcoholics prior to drinking. Addict. Behav. 23, 251–255.

Stormark, K.M., Nordby, H., Hugdahl, K., 1995. Attentional shifts to emotionally charged cues: behavioral and ERP data. Cognition Emotion 9, 507–523.

Thayer, J.F., Friedman, B.H., 1997. The heart of anxiety: a dynamical systems approach. In: Vingerhoets, A. (Ed.), The (Non)Expression of Emotions in Health and Disease. Springer, Amsterdam.

Thayer, J.F., Friedman, B.H., Borkovec, T.D., 1996. Autonomic characteristics of generalized anxiety disorder and worry. Biol. Psychiatry 39, 255–266.

Thayer, J.F., Friedman, B.H., Borkovec, T.D., Johnsen, B.H., Molina, S., 2000. Phasic heart period to cued threat and non-threat stimuli in generalized anxiety disorder 37 (3), 361–368.

Timberlake, W., 1994. Behavior systems, associationism, and Pavlovian conditioning. Psychonomic Bull. Rev. 1, 405–420.

Verrier, R.L., 1987. Mechanisms of behaviorally induced arrhythmias. Circulation 76 (Suppl. I), I48–I56.
