# Supplementary material for: Use of a Smartphone-Based App in an Online Behavioral Neuroscience Lab Course
Source: J Undergrad Neurosci Educ. 2026 Jul 1;24(2):34–43. doi: 10.59390/001c.163102 (PMC13625900; doi:10.59390/001c.163102)
Supplement: Supplementary file 4 [file junejournal_2026_24_2_163102_348894.docx]

**Appendix A**

**Research Project Assignments**

The research project included a variety of assignments meant to help students develop the theoretical background of their study and gain a basic understanding of research methods for determining the outcomes of a stress intervention method (Figure 2). Below are descriptions of the different assignments used throughout the semester. All assignments are also available at: https://doi.org/10.6084/m9.figshare.30025636.

*Assignment 1*. The objectives were for students to 1) identify what heart rate variability is and 2) install and successfully use the app. Students watched a 3-minute video (<https://www.youtube.com/watch?v=dqGN8aOus_U>) that defines heart rate variability and took a short quiz on the main ideas. Next, students were given a link (to the "Camera HRV" app in the Apple Store ( <https://apps.apple.com/us/app/camera-heart-rate-variability/id788460316>) and the Android-compatible Play Store (https://play.google.com/store/apps/details?id=com.asma.camerahrv&hl=en). Depending on the operating system, the app is available for between $4.99 and $6.99. Students completed a survey about the installation and use of the app, allowing me to contact those who experience difficulty.

*Assignment 2*. The objective of assignment 2 was for students to identify the main ideas of the Neurovisceral Integration Model. Students first watched a 21-minute video (created by the author) that summarizes the model. To help students focus on the aspects of the model that are most important for their successful completion of the project, they filled in missing information on 10 statements concerning the model.

*Assignment 3*. Students read an article about a study comparing the effects of a mindfulness meditation intervention with a dog-assisted therapy intervention and a no-intervention control group (Shearer et al., 2016). The objectives of Assignment 3 were for students to 1) identify the effects of mindfulness meditation on student anxiety, mood, and HRV; and 2) identify the procedures and control conditions used in this longitudinal study. Students submitted responses to a series of questions pertaining to the background methods, results and conclusions of the study.

*Assignment 4*. Students read an article that examines the effects of slow-paced breathing on mood, executive function and heart rate variability (Laborde et al., 2022). The objectives were for students to 1) identify what slow-paced breathing is; 2) explain why it may improve mood, decrease anxiety, improve executive function and increase HRV; 3) identify the effects of slow-paced breathing in the study; and 4) identify the procedures used. As in Assignment 3, students responded to a series of questions pertaining to the background, method, results and conclusions of the study.

*Assignment 5*. Students completed the research proposal assignment indicating their chosen intervention. The goal of this assignment was for students to connect the Neurovisceral Integration Model with specific predictions regarding their chosen stress intervention and to practice the skills involved in scientific inquiry in the context of a course in neuroscience. Options included a particular type of meditation, slow-paced breathing, or another breathing intervention. Students were asked to propose that they practice the intervention for at least 10 minutes in accordance with previous research (e.g., Cheng et al., 2019). Students were prompted to describe the intervention in detail and explained their rationale for selecting it. Students were required to use HRV as one measure and chose from subjective stress level, rumination, or state anxiety as an added outcome. Current stress level was measured using a single question with a 9-point scale ranging from “no stress” to “extreme stress”. Rumination was assessed using three state ruminative thoughts questions from Kocsel et al. (2019). State anxiety was measured using the Spielberger State-Trait Anxiety Inventory (Speilberger, 1983). Students also described their research protocol, including the time of day they would implement their intervention. The instructor provided detailed feedback and suggestions on the research proposal.

*Assignment 6*. In Assignment 6, students revised the proposal using instructor feedback.

*Assignment 7*. Students completed baseline measurements of their mood and HRV on three different days throughout the week. Measurements had to be taken at the prescribed time. First, baseline HRV was measured, and students answered all questions regarding their stress, rumination, and/or state anxiety. Then, students sat quietly at the same time as their intervention. HRV and mood were assessed again afterward. Differences from baseline scores were calculated.

*Assignment 8*. Similar to the baseline procedure, students began with a baseline assessment of HRV and stress, rumination, and/or state anxiety. Subsequently, students engaged in their selected intervention for at least 10 minutes on 3 days of the week. HRV and stress, rumination, and/or state anxiety were assessed again afterward.

*Assignment 9*. Students prepared a presentation summarizing their project, and a 150-word abstract. The presentation includes key information from the background literature developed previously in the course. Students described the intervention they selected, the rationale for that selection, and a method section of their study, based on their proposal. Their results were presented in a table. Students compared data from the three baseline days with the intervention days and described any improvement on the measures, including HRV. Since results were based only on their own data, students did not analyze the data statistically.
