# Supplementary material for: Use of a Smartphone-Based App in an Online Behavioral Neuroscience Lab Course
Source: J Undergrad Neurosci Educ. 2026 Jul 1;24(2):34–43. doi: 10.59390/001c.163102 (PMC13625900; doi:10.59390/001c.163102)
Supplement: Supplementary file 5 [file junejournal_2026_24_2_163102_348896.docx]

Video reviewing the Neurovisceral Integration Model

<https://csuci-my.sharepoint.com/:v:/g/personal/beatrice_deoca_csuci_edu/IQAmDNMUqxV1X_WUXFs6ACdpASqLjIo7f8UiDKiLhy3Zypo?e=xuc9HQ>
