# Supplementary material for: Use of a Smartphone-Based App in an Online Behavioral Neuroscience Lab Course
Source: J Undergrad Neurosci Educ. 2026 Jul 1;24(2):34–43. doi: 10.59390/001c.163102 (PMC13625900; doi:10.59390/001c.163102)
Supplement: Supplementary file 6 [file junejournal_2026_24_2_163102_348899.docx]

**Research Proposal Hypothesis and Methodology Revision**

**Please edit the last part of your proposal containing the Hypothesis Statement and Methods 1-4 using the feedback provided in the research proposal assignment. I would have left you detailed feedback on your submitted file or in the submission comments. If you are unable to view the feedback, please let me know.**

**Please highlight all the new text added to these sections of the proposal as you addressed the suggestions or comments I left on the previous assignment.**

Hypothesis Statement

Develop a hypothesis regarding the treatment (intervention) you selected. Specifically, describe how you expect your intervention (breathing or mindfulness meditation) would affect the two dependent variables (stress and heart rate variability).

Method 1

Summarize who the participant is. In other words, provide information that would be included if this was a case study. You can provide your age (feel free to provide a range rather than your exact age, if you prefer), college student status (full time, part time, junior, senior), etc.

Method 2

What is the independent variable (intervention) in your study? Describe your intervention and provide any relevant links to websites if you will be using a guided meditation or breathing activity found online.

Method 3

State the dependent variables. Summarize how you plan on measuring stress and HRV immediately after each session.

Method 4

Describe when you plan on completing the intervention. Also note how often you will practice the intervention per week. The minimum number of times is 3 times per week on separate days.
