# Supplementary material for: Use of a Smartphone-Based App in an Online Behavioral Neuroscience Lab Course
Source: J Undergrad Neurosci Educ. 2026 Jul 1;24(2):34–43. doi: 10.59390/001c.163102 (PMC13625900; doi:10.59390/001c.163102)
Supplement: Supplementary file 7 [file junejournal_2026_24_2_163102_348900.docx]

Name:

Background 1

8 points

In 2-3 sentences, describe the effects of stress on physical and mental health. A good source is the introduction of the article titled, "Effects of a Brief Mindfulness Meditation Intervention on Student Stress and Heart Rate Variability" . This is Article #1.

Background 2

10 points

In 3-5 sentences, define what heart rate variability is in your own words and describe how stress affects a person’s physiology including heart rate variability. You can use Article #1 and Article #2 as sources for this. You can also use the Neurovisceral Integration Model article by Thayer and Lane (2000). This is the article I summarized in the lab for Chapter 1.

Background 3

10 points

In 1-2 paragraphs, summarize the Neurovisceral Integration Model, including the Central Autonomic Network (CAN) and the way that the CAN may influence the body through the sympathetic and parasympathetic nervous systems. Be sure to describe how the model links heart rate variability with better emotion regulation and executive function. The Neurovisceral Integration Model fact sheet assignment is an excellent source for this information.

Background 4

2 points

State the type of intervention you will use in your study. Choose a type of slow-paced breathing activity or a specific meditation activity. Why did you choose this intervention for yourself?

Background 5

10 points

In this section, please summarize in 2-3 paragraphs the relevant study (either Article #1 or Article #2) that uses the same intervention (breathing or mindfulness meditation). In your summary, please describe the procedures used and the outcomes. This will provide some background on what is already known about the effectiveness of the intervention you've selected.

Background 6

5 points

Explain how the intervention you chose would help the physiological and psychological stress reaction, according to the Neurovisceral Integration Model.

Hypothesis Statement

10 points

Write a hypothesis statement regarding the intervention you selected. Specifically, describe how you expect your intervention (breathing or mindfulness meditation) would affect the two dependent variables (stress and heart rate variability).

Conclusion 1

5 points

What could be learned about the effectiveness of the intervention after having completed the study?

Conclusion 2

8 points

What could be learned about how the Central Autonomic Network functions due to your study?

Conclusion 3

5 points

How could you use this information to better cope with stress?

Method 1

5 points

Summarize who the participant is. In other words, provide information that would be included if this was a case study. You can provide your age (feel free to provide a range rather than your exact age, if you prefer), college student status (full time, part time, junior, senior), etc.

Method 2

8 points

What is the independent variable (intervention) in your study? Describe your intervention and provide any relevant links to websites if you will be using a guided meditation or breathing activity found online.

Method 3

5 points

State the dependent variables. Summarize how you plan on measuring stress and HRV immediately after each session.

Method 4

5 points

Describe the time of day you will practice the intervention and the number of times per week you will practice. The minimum number of times is 3 times per week on separate days.

References

4 points

Please cite your sources in the text and you have a separate References page at the end in APA style.
