# Supplementary material for: Use of a Smartphone-Based App in an Online Behavioral Neuroscience Lab Course
Source: J Undergrad Neurosci Educ. 2026 Jul 1;24(2):34–43. doi: 10.59390/001c.163102 (PMC13625900; doi:10.59390/001c.163102)
Supplement: Supplementary file 8 [file junejournal_2026_24_2_163102_348902.docx]

Article #2

**The Influence of Slow-Paced Breathing on Executive Function**

Laborde, S., Allen, M. S., Borges, U., Hosang, T. J., Furley, P., Mosley, E., & Dosseville, F. (2022). The influence of slow-paced breathing on executive function. *Journal of Psychophysiology*, *36*(1), 13–27. <https://doi-org.ezproxy.csuci.edu/10.1027/0269-8803/a000279>

**Introduction**

1. What does the Neurovisceral Integration Model say about how slow paced breathing should affect cardiac vagal activity and how vagal activity should affect executive performance?
2. What is slow paced breathing?
3. What are the 3 core executive functions and how is each measured?
4. Does accuracy on the Stroop task reflect inhibition?
5. Is better accuracy on the Stroop task associated with lower or higher resting heart rate variability?
6. Is better performance on the AOSPAN task associated with lower or higher resting heart rate variability?
7. Is better performance on the Wisconsin Card Sorting Task associated with lower or higher resting heart rate variability?
8. What was the purpose of the study?
9. What were the hypotheses? These are nicely summarized in paragraph 1 of the Discussion.

**Method**

Participants

1. Number of participants in the final sample.

Design Overview/Procedure

2. Describe the treatment conditions (Slow Paced Breathing and Control).
3. How were the participants trained in slow breathing?
4. Describe each of the 2 sessions the participants completed.

Measure

1. How long was resting heart rate variability measured?
2. What does RMSSD measure?
3. List the 3 measures of executive function that were administered.

**Results**

Skip this section. You will learn about the results in the discussion section.

**Discussion**

1. Did slow-paced breathing improve executive function?
2. Did slow paced breathing influence heart rate variability during the slow breathing?
3. Did slow paced breathing influence heart rate variability after the slow breathing stopped?
4. What are the implications of these findings for college students hoping to perform well on exams or other assignments?
