# Supplementary material for: Use of a Smartphone-Based App in an Online Behavioral Neuroscience Lab Course
Source: J Undergrad Neurosci Educ. 2026 Jul 1;24(2):34–43. doi: 10.59390/001c.163102 (PMC13625900; doi:10.59390/001c.163102)
Supplement: Supplementary file 9 [file junejournal_2026_24_2_163102_348904.docx]

Jigsaw Technique

**Effects of a Brief Mindfulness Meditation Intervention on Student Stress**

**and Heart Rate Variability**

Shearer, A., Hunt, M., Chowdhury, M., & Nicol, L. (2016). Effects of a brief mindfulness meditation intervention on student stress and heart rate variability. *International Journal of Stress Management*, *23*(2), 232–254. https://doi-org.ezproxy.csuci.edu/10.1037/a0039814

**Introduction**

1. Identify what “emotion-based” coping is and what the authors say about it. Here is some general information about emotion based coping (also known as emotion-focused coping) <https://www.simplypsychology.org/stress-management.html>
2. What is MSBR and what types of traits is it correlated with?
3. What have previous studies found about MSBR’s effects on pain, anxiety and depression?
4. What is an active control group and why would it be better than a no-treatment control?
5. What type of active control was used in the study? What are some benefits of this type of animal-assisted therapy?
6. Why are physiological measures a good idea? In your answer, define what “demand characteristics” are. Here is a definition and example - <https://www.verywellmind.com/what-is-a-demand-characteristic-2795098>
7. What is HRV and what is it associated with?
8. How is HRV linked to emotion regulation?
9. What was the purpose of the study?

**Method**

Participants

1. Number of participants in each group. Who were the participants?

Design Overview/Procedure

2. Describe the treatment conditions (MSBR and dog therapy).
3. Describe the self-report measures and when they were taken.
4. What was the challenge activity and when did it occur? Why was this activity chosen as a challenge?
5. What was measured after the challenge activity?

Measure

6. List the surveys/questionnaires used and what each one measured.
7. When was HRV collected?

Procedure

8. Describe the De-stress with dogs procedure.
9. Describe the mindfulness intervention.
10. Describe how the no-treatment control was done.
11. When was the challenge activity (WAIS) done?
12. What measures were taken during or after the challenge?

**Results**

Skip this section. You will review read about the study in the discussion section.

**Discussion**

1. What did the authors say about the effects of the dog therapy in the active control group compared to the effects of mindfulness training during the 4 weeks of the sessions?
2. Summarize the HRV scores in the 3 groups during the challenge.
3. What was different about the amount of time spent in mindfulness training in this study versus the usual MSBR training?
4. Are the HRV results consistent with what other researchers have found?
5. List the limitations of the study.
6. What are the implications of these findings for college students hoping to cope with academic stressors?
7. In implications, the authors describe some of the strengths of their study, including the use of an active control and an objective, physiological measure like HRV. Summarize what they said about these.
