# Supplementary material for: Use of a Smartphone-Based App in an Online Behavioral Neuroscience Lab Course
Source: J Undergrad Neurosci Educ. 2026 Jul 1;24(2):34–43. doi: 10.59390/001c.163102 (PMC13625900; doi:10.59390/001c.163102)
Supplement: Supplementary file 10 [file junejournal_2026_24_2_163102_348905.docx]

HRV Survey. This survey allows the instructor to monitor the successful purchase and use of the app by students.

Question 1

Were you able to download the app to your phone?

No

Yes

Question 2

Does the app seem to work on your phone?

No

Yes

Question 3

Were you able to get a good quality measure of your heart rate variability using the app?

No

Yes

Question 4

If you were not able to successfully use the app to sample your pulse and heart rate variability, please explain what happened. I will try to help you to the best of my ability.
