# Supplementary material for: Use of a Smartphone-Based App in an Online Behavioral Neuroscience Lab Course
Source: J Undergrad Neurosci Educ. 2026 Jul 1;24(2):34–43. doi: 10.59390/001c.163102 (PMC13625900; doi:10.59390/001c.163102)
Supplement: Supplementary file 11 [file junejournal_2026_24_2_163102_348906.docx]

Heart rate variability quiz. This quiz assesses basic understanding of heart rate variability.

Question 1

When a person's heart is beating rapidly, the interval between beats will be shorter.

True (correct)

False

Question 2

Heart rate is measured using

beats per minute (BPM) (correct)

rMSSD

Question 3

Heart rate variability is the same as heart rate.

True

False (correct)

Question 4

As we inhale, our heart tends to beat more rapidly.

True (correct)

False

Question 5

As we exhale, our heart tends to beat more slowly.

True (correct)

False

Question 6

Heart rate variability (HRV) is measured using:

beats per minute (BPM)

rMSSD (correct)

Question 7

rMSSD is based on

the intervals of time between heart beats (correct)

the size of the electrical signal created by the heart.
