# Supplementary material for: Use of a Smartphone-Based App in an Online Behavioral Neuroscience Lab Course
Source: J Undergrad Neurosci Educ. 2026 Jul 1;24(2):34–43. doi: 10.59390/001c.163102 (PMC13625900; doi:10.59390/001c.163102)
Supplement: Supplementary file 12 [file junejournal_2026_24_2_163102_348908.docx]

1. Emotion emerges from hormones, blood pressure, ___________, respiration, muscle tension and ____________ processing.
2. Individuals who suffer from depression and anxiety may have a hard time inhibiting what? _____________________________________________
3. What is “vagal tone”?__________________________________________
4. Heart rate ________ is used as an indicator of vagal tone.
5. True or false: The sympathetic nervous system slows down the heart rate.
6. High vagal tone is associated with ____________ emotion regulation and behavioral flexibility.
7. The prefrontal cortex, amygdala and hypothalamus are brain structures included in the __________ Autonomic Network.
8. True or false: The CAN controls the activity of the heart by controlling the sympathetic and parasympathetic nervous systems.
9. What is known about heart rate variability in those with depression?___________________________________________________
10. What is the benefit of negative feedback in terms of anxiety?_______________________________________________________
